# BioTester: an AI-driven automated testing framework for identifying potential quality risks in bioinformatics software

**DOI:** 10.1093/bib/bbag452

**Published:** 2026-09-16

**Authors:** Xin Lian, Jiayin Wang, Xiaoyan Zhu, Sizhe Dang, Tianxiang Xu

**Affiliations:** School of Computer Science and Technology, Xi'an Jiaotong University, Xi'an 710049, Shaanxi, China; School of Computer Science and Technology, Xi'an Jiaotong University, Xi'an 710049, Shaanxi, China; School of Computer Science and Technology, Xi'an Jiaotong University, Xi'an 710049, Shaanxi, China; School of Computer Science and Technology, Xi'an Jiaotong University, Xi'an 710049, Shaanxi, China; School of Computer Science and Technology, Xi'an Jiaotong University, Xi'an 710049, Shaanxi, China

**Keywords:** bioinformatics software, automated software testing, large language model, test oracle generation, retrieval-augmented generation, biomedical software reliability

## Abstract

Bioinformatics software plays a critical role in clinical applications such as cancer screening and genetic disease diagnosis, where comprehensive quality management is essential for ensuring the accuracy and reliability of downstream analysis. However, current validation practices rely heavily on manually designed simulation experiments, which are labor-intensive and limited in their ability to systematically identify potential quality risks under certain scenarios. In this study, we first construct a benchmark by simulating subtle implementation-level defects in bioinformatics programs. We then propose BioTester, an oracle-based automated testing framework that integrates software testing techniques to support more comprehensive quality assessment of bioinformatics software. BioTester integrates retrieval-augmented LLMs with a differential testing strategy to address the long-standing oracle problem in bioinformatics software testing, demonstrating superior defect-detection performance over existing methods on the constructed benchmark. Finally, applying BioTester to real-world bioinformatics software demonstrates its practical effectiveness and highlights the value of automated testing as a generalizable complement to existing validation practices for improving software reliability in biomedical applications.

## Introduction

With the rapid development of genomics and computational biology, bioinformatics software has become indispensable for analyzing the rapidly growing volume of biological data. These tools play a central role in a wide range of clinical and biomedical applications, including early tumor screening [1–4], newborn genetic disorder detection [5–8], and patient stratification for personalized treatment [9–11]. In such scenarios, analytical results generated by bioinformatics pipelines can inform downstream diagnostic and therapeutic decisions. Consequently, the reliability and quality of bioinformatics software are of critical importance for both biological research and clinical practice. However, the rapid expansion of bioinformatics software has not always been accompanied by standardized software engineering practices. Many tools are developed primarily to address specific biological problems, and software testing with comprehensive documentation is not consistently adopted [12, 13]. These development characteristics, together with the heterogeneity of bioinformatics projects, raise concerns about latent quality risks that may affect analytical reliability [14–16].

Testing bioinformatics software to ensure their quality is challenging because its complex statistical models and algorithms make expected outputs difficult to specify, resulting in the well-known oracle problem [15, 17, 18]. To mitigate this limitation, bioinformatics researchers typically either design specialized benchmark test suites and manually crafted test cases for validating bioinformatics systems [19, 20], or rely on property-based testing strategies, such as assessing whether program behaviors satisfy necessary relational properties across multiple executions under systematically transformed inputs [21]. However, extracting such domain-specific relations and designing tool-specific validation rules typically require substantial expert knowledge and manual effort, which significantly limits the scalability and practicality of this approach. Furthermore, empirical studies have revealed that some intrinsic relational properties do not consistently hold in widely used bioinformatics tools, including sequence alignment [17, 22], variant calling [23], and protein function prediction software [24]. These violations suggest the existence of latent implementation defects that may propagate through downstream analytical pipelines. Unfortunately, existing testing approaches are often unable to localize the root causes of such issues or provide actionable guidance for defect correction. Consequently, there remains a critical need for automated testing methodologies that can systematically detect potential defects in bioinformatics software while reducing the reliance on extensive domain expertise and manual intervention.

In recent years, rapid advances in natural language processing techniques have stimulated growing interest in applying large language models (LLMs) to automated test case generation at unit level [25–28]. By synthesizing test inputs and expected behaviors, these approaches aim to precisely localize defective functions and enhance the intelligence of software quality assurance processes. However, directly applying existing LLM-based automated unit testing methods to bioinformatics software substantially limits their effectiveness in reliable oracle generation. In particular, two fundamental challenges arise. First, oracle inference requires accurate recovery of functional specifications. Bioinformatics functions often encapsulate intricate domain-specific logic grounded in implicit biological knowledge. Yet code documentation in bioinformatics software is frequently concise, incomplete, or even inconsistent with the underlying implementation [16], offering limited reliable semantic guidance. Second, due to the noise-tolerant nature of Transformer-based architectures [29], LLMs may exhibit limited sensitivity to subtle logical defects in source code [26]. In practice, silent semantic deviations are common in programming, such as incorrect argument passing and improper boundary-case handling. For instance, invoking a function with reversed argument order (e.g. calling **pow(2,3)** instead of **pow(3,2)**) yields syntactically valid yet semantically incorrect results without triggering runtime errors. When such imperfect implementations are treated as reliable semantic references, LLM-driven oracle construction risks a ”garbage-in, garbage-out” effect, propagating embedded errors into predicted expected outputs and undermining the trustworthiness of generated tests. These limitations indicate that naive LLM-driven test generation is insufficient for bioinformatics software. Instead, domain-adapted reasoning and behavior-based oracle inference mechanisms are required.

To address these challenges and bridge the gap in intelligent quality assurance for general bioinformatics software, we present BioTester, a novel LLM-based automated testing framework tailored to resolve the long-standing oracle problem in this domain. BioTester integrates domain-aware semantic recovery with behavior-driven oracle inference. It combines a fine-tuned LLM, a code knowledge graph, and software-specific retrieval to infer functional intent from complex code dependencies and implicit biological logic. Based on the recovered semantics, BioTester generates reference implementations and applies differential testing against the original code, enabling unit-level consistency checking and localization of high-risk discrepancies. Beyond controlled experiments on benchmark datasets, we further evaluate BioTester through case studies on real-world open-source bioinformatics tools. These case studies characterize representative detected discrepancies and examine their potential effects on downstream analytical performance. These findings not only validate the effectiveness of the proposed framework but also highlight the practical value of BioTester as a scalable solution for reliability assurance in real-world bioinformatics software.

## Materials and methods

### Construction of a bioinformatics software defect benchmark

To support quality improvement in bioinformatics software, we followed the software selection procedure used in previous studies to collect widely used bioinformatics projects from GitHub [16]. These projects were used as the target software for applying BioTester in real-world settings. Besides, we constructed a bioinformatics software defect benchmark based on these real projects to provide a quantitative basis for evaluating the defect-detection capability of different testing methods. The filtering process is summarized below.

- **Repository collection.** We systematically collected candidate repositories from GitHub that were created between 1 January 2010 and 1 October 2025. To improve domain coverage, the search process incorporated representative bioinformatics keywords spanning multiple analytical areas, including genomics, transcriptomics, single-cell analysis, sequence analysis, structural variation analysis, and functional genomics. Keywords were matched against repository names, descriptions, topics, and README files, yielding a broad collection of candidate bioinformatics software projects.
- **Language and source-code filtering.** Repositories without publicly available source code were excluded. Given the widespread adoption of Python in modern bioinformatics software development [16], we restricted our study to Python-based repositories. Projects lacking executable Python source files or containing only auxiliary scripts were removed.
- **Manual curation and quality control.** The remaining repositories were manually reviewed to ensure that the software itself constituted the primary scientific contribution. Repositories without associated peer-reviewed publications were excluded, as were projects linked only to non-peer-reviewed materials (e.g. technical reports, course projects, or theses). Duplicate repositories, deprecated projects, and software wrappers with minimal original implementation were also removed.
- **Impact and adoption filtering.** To prioritize bioinformatics software with demonstrated practical relevance and community adoption, we further filtered repositories based on software adoption and scientific impact. For software published >3 years prior to repository collection, we required the corresponding GitHub repository to have at least 10 stars and the associated publication to have accumulated at least 20 citations. This criterion helped exclude minimally adopted projects while retaining software with demonstrated real-world influence in biomedical research workflows.
- **Domain-aware benchmark selection.** From the curated repository pool, we selected 33 representative bioinformatics tools covering diverse analytical domains. The final selection additionally considered software maturity, community adoption, testing feasibility, and computational practicality, enabling comprehensive yet scalable evaluation across heterogeneous bioinformatics workflows.

Table S1 in the supplementary materials summarizes the bioinformatics software included in our quality assessment, covering a broad spectrum of clinically relevant computational tasks. The selected software spans major areas: sequence and genome analysis, genome variant analysis, evolutionary bioinformatics, functional genomics and systems biology, and protein and structural bioinformatics, whose application ranges from disease screening to precision medicine. Sequence analysis, including tasks such as read alignment and genome assembly, provides the fundamental data processing required for numerous genomic analysis and is widely used in applications such as pathogen identification and clinical genome reconstruction [30–32]. Variant detection focuses on identifying genomic alterations from sequencing data and plays a critical role in clinical scenarios including noninvasive prenatal testing, early cancer screening, and genetic disease diagnosis [1, 4, 33]. Functional inference involves higher-level analysis such as protein sequence interpretation and single-cell transcriptomic analysis, which are essential for understanding gene functions, characterizing cellular heterogeneity, and guiding precision medicine strategies [34–36]. The tools are actively maintained, with latest updates ranging from 2016 to 2025, and show substantial community adoption, with GitHub stars reaching up to 14.6k. Many of these tools have been published in high-impact journals, including Nature, Nature Communications, PLOS Computational Biology, Nucleic Acids Research, Bioinformatics, and Briefings in Bioinformatics, with citation counts of up to 43 672, underscoring their broad scientific relevance and clinical influence.

Mutation testing is a widely used strategy for evaluating defect-detection capability by injecting small syntactic changes into a program to simulate potential implementation-level faults [37, 38]. Each modified version, called a mutant, is expected to introduce a subtle behavioral deviation from the original program. If a testing method can detect the behavioral difference between the original implementation and the mutant, the mutant is regarded as killed; otherwise, it remains undetected. Following this principle, we constructed a mutation-based defect benchmark from real bioinformatics software. To balance the efficiency of large-scale controlled experiments and the diversity of bioinformatics software, we manually curated independently executable function units with controllable execution time and stable, reproducible behavior from collected bioinformatics tools. The candidate functions were additionally reviewed through manual code inspection and behavioral validation, and no obvious semantic defects were identified, allowing them to serve as reliable reference implementations for mutation testing. For each function unit, we generated first-order mutants using five mutation operators, as shown in Table 1: arithmetic operator mutation, Boolean logic mutation, comparison relation mutation, constant mutation, and function-call argument mutation. For each function unit, we attempted to generate five mutants, one for each mutation operator. When a specific operator could not be applied because no valid mutation locations existed in the function, the operator was skipped. Finally, the benchmark used in our experiments contains 790 units with 2441 mutants from 18 bioinformatics software projects.

**Table 1:** Design of mutation operators

Mutation type	Description	Example
OperatorReplacement	Replaces arithmetic operators (e.g. + *→* −, * *→* /)	a + b *→* a − b
BooleanReplacement	Flips Boolean logic (e.g. True *→* False, and *→* or)	if a and b *→* if a or b
CompareReplacement	Modifies comparison relations (e.g. < *→* <=, == *→* !=)	x < y *→* x <= y
ConstantReplacement	Alters constant values (including integers, floats, and strings)	range(10) *→* range(11)
FunctionCallArgReplacement	Replaces, removes, or reorders function call arguments	pow(2, 3) *→* pow(3, 2)

### Proposed methodology

**Figure 1:**
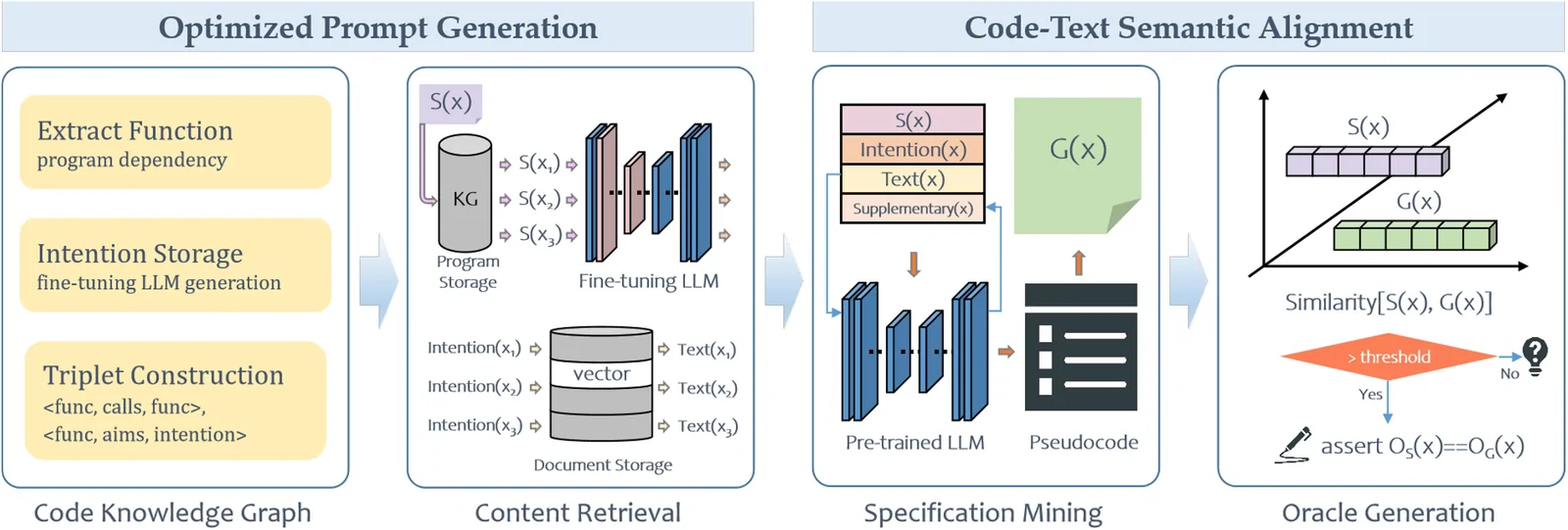
The automated testing framework of BioTester.

To improve the quality assurance for general bioinformatics software, we present a novel LLM-based automated test case generation framework, BioTester, to enable fine-grained defect detection at the unit level. As illustrated in Fig. 1, BioTester consists of four major components designed to support two key processes: optimized prompt generation for guiding the LLM to infer precise functional intent, and code–text semantic alignment for reliable oracle construction.

#### Code knowledge graph construction

Owing to the domain-specific nature of bioinformatics algorithms, brief code comments and source code alone often provide insufficient semantic information for LLMs to infer the functional intent of test units. To enhance the LLM’s understanding of both algorithmic logic and contextual knowledge, BioTester constructs a function-level knowledge graph through static analysis of the source code. Specifically, we parse the abstract syntax tree of each Python file and extract function invocation relationships as triples in the form of **[caller function, relation, callee function]**. If the callee is a Python built-in function, the relation is labeled as **uses**; otherwise, it is labeled as **calls**, indicating an invocation of another defined or third-party function. In addition, we fine-tune an LLM to generate concise intent summaries for functions in bioinformatics software and incorporate them into the graph as intention-oriented triples, **[function, aims, intention]**. As a result, the knowledge graph encodes both structural dependencies and functional intent, supporting more targeted and semantically aligned retrieval of relevant textual evidence from scientific literature in the subsequent step.

#### Content retrieval

Due to the high demand for domain-specific knowledge in bioinformatics, LLMs often struggle to accurately infer the expected behavior of code units in this field. To address this limitation without the computational overhead of retraining a domain-specific LLM from scratch, we adopt a retrieval-augmented approach combined with supervised fine-tuning to enhance LLM’s understanding of specialized bioinformatics logic while achieving competitive performance with minimal additional cost [39, 40].

Firstly, a fine-tuned LLM is utilized to concisely summarize each function’s intent in natural language. These generated intents are stored in the form of knowledge graph triplets **[function, aims, intention]** as mentioned, supporting the following retrieval. As shown in Fig. 1, given a function unit *x*, its source code represents as *S(x)*, and the constructed knowledge graph is used to extract its internal invocations like functions $S(x_{1})$, $S(x_{2})$, and $S(x_{3})$. And their stored intentions can be also returned as $Intention(x_{1})$, $Intention(x_{2})$, and $Intention(x_{3})$.

Then, the reference literature serves as a knowledge database for dynamic retrieval, where all sentences are first extracted from provided files using rule-based segmentation. Each sentence is then embedded into a fixed-dimensional vector using the fine-tuned LLM to ensure consistency in representation space. All sentence embeddings are indexed with FAISS [41] to support efficient similarity search. Given intention descriptions associated with unit under test such as $Intention(x_{1})$, $Intention(x_{2})$, and $Intention(x_{3})$, their embeddings are computed and the top-k most semantically similar sentences from the indexed literature corpus $Text(x_{1})$, $Text(x_{2})$, and $Text(x_{3})$ are retrieved based on vector similarity like L2 (Euclidean) distance. These retrieved sentences serve as supplementary knowledge, enriching each test unit with relevant domain context. Accordingly, the LLM is allowed to access task-relevant literature without requiring full-scale domain-specific pretraining, thereby balancing effectiveness and efficiency.

#### Specification mining

After enriching the prompt with relevant domain knowledge, BioTester performs specification mining and oracle generation through multi-stage LLM analysis. It then adopts differential testing to verify the function under test by identifying inputs that produce inconsistent outputs across implementations [26, 28]. Instead of arbitrarily generating equivalent variants, BioTester uses chain-of-thought reasoning and retrieved domain knowledge to guide the LLM in constructing a reference implementation that reflects the intended functional semantics.

In the first stage, the LLM is prompted with the intention description *Intention(x)* and the retrieved domain-specific references *Text(x)* corresponding to the test unit, enabling it to form a comprehensive contextual understanding of the algorithmic background. This step produces an auxiliary knowledge representation denoted as *Supplementary(x)*, which serves as additional expert knowledge for subsequent inference. Next, guided by the enriched context, the LLM infers the expected behavior of the target function by reasoning over the original implementation *S(x)*. Here, *S(x)* is used solely to convey programming conventions and structural cues, while its internal logic is treated as potentially unreliable due to possible implementation defects. Based on the inferred behavioral description, the LLM is then prompted to generate a pseudocode-level reconstruction that encapsulates the algorithm’s intended specification while abstracting away implementation errors. Finally, this reconstructed pseudocode is translated back into fully executable code *G(x)* within the same programming framework by LLM. The resulting *G(x)* thus serves as a refined and semantically grounded reference implementation, providing a reliable basis for subsequent oracle-based differential testing. As a result, discrepancies between the original implementation and this refined version are thus more indicative of potential flaws or subtle errors in the original code.

#### Oracle generation

Before conducting oracle-based testing, BioTester performs an additional layer of semantic analysis to enhance the reliability of differential testing. Specifically, since the original implementation *S(x)* and the refined version *G(x)* are both derived from the same intended functionality, with the latter benefiting from richer domain-specific knowledge, comparing their semantic consistency can partially reflect whether the unit under test faithfully realizes the logic described in the retrieved textual specification. To quantify this alignment, the CodeT5-base model [42] pretrained on code is leveraged to encode both *S(x)* and *G(x)* into dense vector representations via mean pooling over the token-level embeddings, which has shown strong performance in code representation learning. The cosine similarity between the two vectors is computed, and a higher similarity indicates stronger semantic alignment, suggesting that the original function code *S(x)* has effectively captured the expected behavior. Conversely, low similarity especially below the preset threshold suggests that *S(x)* may deviate from the intended behavior, prompting a flag for potential faults. Meanwhile, as the dynamically retrieved content may not always provide stable references, the similarity score is used only as a supplementary indicator in the final testing analysis. Subsequently, all cases are to be tested with oracles to deeply examine differences in detailed execution logic.

Finally, to maximize bug detection coverage, the LLM is prompted to generate diverse and valid test cases that traverse as many executable paths as possible through control-flow analysis. For each test case, the corresponding outputs from the refined implementation $O_{G}(x)$ are considered as oracles for $O_{S}(x)$, which are subsequently embedded as a set of assertions in the form of **assert $O_{S}(x)$ == $O_{G}(x)$** statements to enable automated testing. Whenever a discrepancy arises, the assertion triggers an alert, indicating a failed test caused by a potential underlying bug in the original implementation.

#### An end-to-end example of BioTester

We illustrate the complete workflow of BioTester using the function **_reference:copies_pure** from CNVkit [1]. The original implementation is shown below.

**Listing 1:**
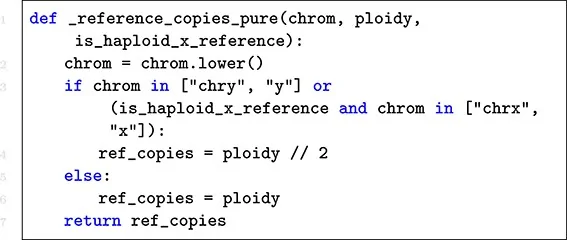
Original implementation from CNVkit

**Figure 2:**
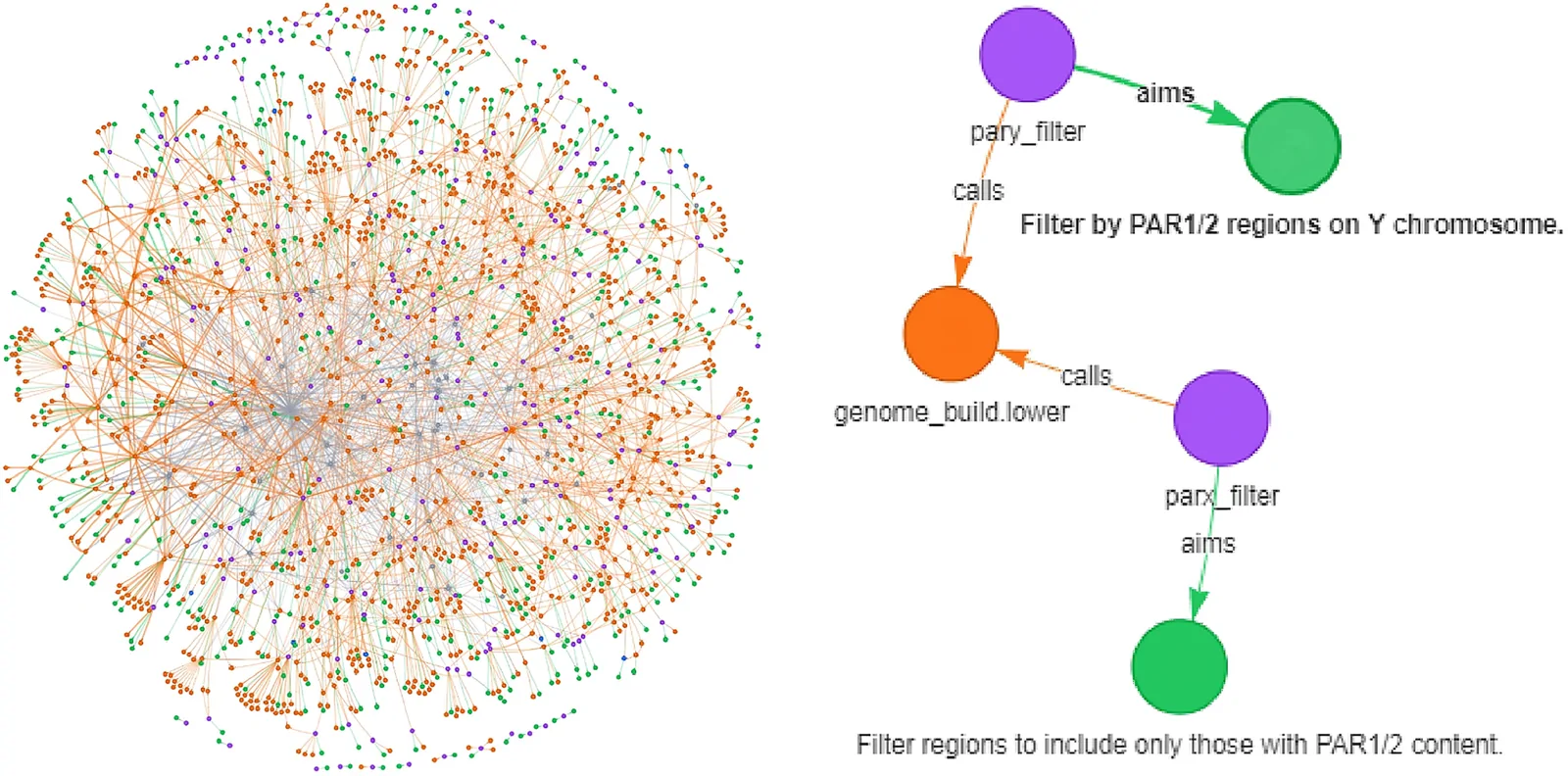
A code knowledge graph example for CNVkit.

BioTester first extracts all functions from CNVkit and collect their dependency relationships. Then it employs the domain-adapted LLM to summarize the functional intention of each function and stores the intention together with the corresponding function node. Figure 2 illustrates an example code knowledge graph constructed for CNVkit, which captures both the structural dependencies and functional intentions of its functions. For details, top-level functions with no incoming edges are colored purple, while intermediate user-defined or third-party functions are marked in orange. Intention nodes, representing the summarized functional purpose of a user-defined function, appear in green, and those with only a single outgoing **aims** edge are shown in blue to denote leaf nodes with annotated intent. Built-in functions and isolated nodes are rendered in gray. For edges, we distinguish relation types using color: **calls** relations are shown in orange, **aims** relations in green, **uses** edges (typically referencing built-in utilities) in gray, and other minor relations in blue. As a result, CNVkit contains 2245 nodes with 3902 edges. For instance, the **_reference:copies_pure** function has no dependency, while the function **pary_filter** shown in Fig. 2 invokes the built-in string normalization operation **genome_build.lower()** and is associated with the intention of filtering PAR1/2 regions on the Y chromosome.

For the target unit **_reference:copies_pure**, BioTester retrieves the intentions of the unit itself and its related dependency units from the code knowledge graph. Therefore, only its own intention is retrieved and used to support subsequent reasoning:

Determine the reference number of chromosome copies in a pure sample.

Using the intention as a query, BioTester retrieves relevant texts from the provided bioinformatics literature corpus [1]:

“The reference command estimates the expected read depth of each on- and off-target bin across a panel of control or comparison samples to produce a reference copy-number profile that can then be used to correct other test samples.”

“The fix command combines a single sample’s on- and off-target binned read depths, removes bins failing predefined criteria, corrects for systematic biases in bin coverage, subtracts the reference log$_{2}$ read depths, and finally median-centers the corrected copy ratios.”

“The call command rounds the log$_{2}$ ratios to the nearest integer absolute copy number given the normal ploidy of each chromosome, or directly maps segment log$_{2}$ ratios to absolute copy number states given a set of numeric thresholds.”

“Each of the methods was evaluated using all of the available approaches for constructing a reference: all normal samples pooled, matched tumor-normal pairs, and tumor-only calling with no normal reference.”

Based on the retrieved textual evidence, BioTester prompts the LLM to reason about the implementation details of the target function:

To understand the implementation of the code snippet, it is essential to recognize the context of determining the reference number of chromosome copies in genomic analysis. The code aims to calculate the number of chromosome copies based on the input chromosome type, ploidy level, and whether the sample is haploid for the X chromosome.

The reference texts highlight the importance of establishing a reference copy-number profile, which is crucial for normalizing test samples and correcting for systematic biases in read depths. The reference copy-number profile is derived from control or comparison samples, and the expected read depth is estimated for both on-target and off-target bins. This process is fundamental in genomic studies, particularly in copy number variation (CNV) analysis, where accurate determination of chromosome copies is necessary for interpreting genomic data.

The code snippet correctly identifies specific chromosomes (Y and X) and adjusts the reference copies based on the ploidy level. For haploid samples, it divides the ploidy by two for the X chromosome, while for other chromosomes, it uses the ploidy directly. This logic aligns with the need to account for normal ploidy levels when determining absolute copy numbers, as mentioned in the reference texts.

There are no apparent bugs in the code snippet; it accurately implements the logic required to determine the reference number of chromosome copies based on the given parameters.

Based on this understanding, BioTester further summarizes the algorithm into pseudocode:

**Listing 2:**
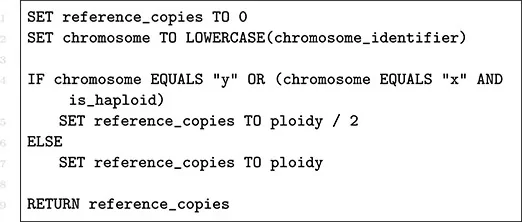
Algorithm description generated by BioTester

BioTester then provides both the algorithm description and the original source code to the LLM, allowing it to learn the function name, input parameters, return format, and programming style of the original function while disregarding its implementation logic. Guided by this information, the LLM translates the algorithm description pseudocode into an executable reference implementation consistent with the original function interface and coding style:

**Listing 3:**
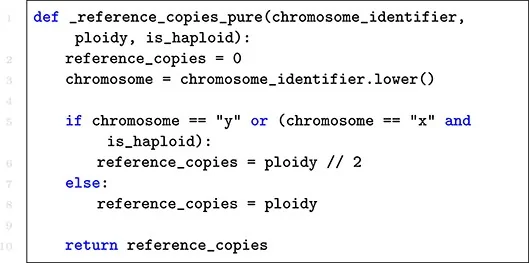
Fixed implementation given by BioTester

Then the semantic similarity of two implementations can be computed as 0.981. BioTester then generates test inputs and executes them on the reference implementation:

**Listing 4:**
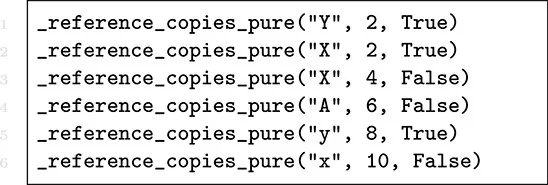
Generated test cases

The resulting outputs are automatically converted into executable assertions:

**Listing 5:**
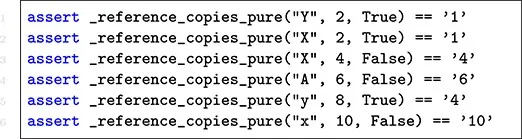
Assertions generated by BioTester

Finally, the generated assertions are executed against the original implementation. All six assertions pass successfully (assert_failed = 0), indicating that the behavior of the original function is consistent with the generated reference implementation and the retrieved domain knowledge. Therefore, BioTester assigns the prediction label 0, meaning that no defect is detected in this function.

### Implementation details

To improve the transparency and reproducibility of our framework, this section provides detailed descriptions of datasets, model configurations, training settings, and deployment strategies.

#### Datasets

We fine-tune the LLM on function–intent pairs constructed from two sources. The primary dataset is curated from four widely used open-source bioinformatics software projects, including CNVkit, CNVpytor, inferCNV, and Scanpy [1, 43–45]. We collect functions that are accompanied by concise and informative natural language descriptions from both GitHub repositories [46–49] and their corresponding documentation hosted on ReadTheDocs [50–53]. This results in 420 annotated function–intent pairs, including 112 from CNVkit, 190 from CNVpytor, 26 from inferCNV, and 92 from Scanpy. To enhance the model’s general code understanding and summarization ability, we further incorporate general datasets including two public benchmarks: 257 Python functions from MBPP [54] and 300 code–comment pairs from the Python subset of CodeSearchNet [55]. These datasets are widely adopted in program synthesis and code summarization tasks. For all datasets, we randomly split the data into 70% for training and 30% for evaluation.

#### Model and fine-tuning configuration

We adopt DeepSeek-R1-Distill-Llama-8B [56] as the base model, considering its strong balance between capability and efficiency. Parameter-efficient fine-tuning is performed using LoRA (Low-Rank Adaptation) [57], with a rank of 64 applied to all trainable layers. The fine-tuning process follows a supervised learning paradigm. The maximum input length is set to 2048 tokens. The model is trained for three epochs with a learning rate of $2\times 10^{-4}$, batch size of 1, and gradient accumulation step of 1. We adopt cosine learning rate scheduling with a warmup ratio of 0.1, and use bf16 precision for efficient training. After fine-tuning, the model is able to generate concise and semantically meaningful intent descriptions for arbitrary function inputs. Experiments were conducted on a server with an NVIDIA RTX 4090 GPU and 64GB RAM.

#### LLM usage in BioTester pipeline

Within the BioTester framework, different LLMs are employed for distinct roles. Specifically, the mentioned fine-tuned DeepSeek-R1-Distill-Llama-8B model is used exclusively for the function intent summarization task, where it generates concise and semantically accurate descriptions of input functions. For all other general-purpose code understanding and generation tasks in the pipeline (e.g. test case generation, oracle construction, and result interpretation), we utilize GPT-4o-mini [58] via the OpenAI API, due to its strong performance and cost-efficiency trade-off. The default APT settings are utilized, i.e. $\mathtt{temperature}=1.0$ and $\mathtt{top}{\_}\mathtt{p}=1.0$. We do not explicitly constrain $\mathtt{max}{\_}\mathtt{tokens}$, allowing it to be automatically determined by the model during generation. To ensure a fair comparison, all LLM-based baseline methods in our experiments also use GPT-4o-mini with consistent default API settings. The framework is model-agnostic, and the underlying LLM can be flexibly replaced depending on available computational resources or application requirements.

## Results

To comprehensively evaluate BioTester, we conduct both controlled experiments and real-world studies on bioinformatics software. The controlled experiments assess the correctness, robustness, and component-wise contributions of the framework, while the real-world studies demonstrate its practical utility in detecting defects in widely used bioinformatics software.

### Performance evaluation of BioTester in controlled experiments

#### Correctness on mutation-based defect detection

To evaluate the effectiveness of BioTester under controlled conditions, we performed mutation-based simulation experiments on the constructed bioinformatics software benchmark. Specifically, we used classical mutation testing to examine whether the generated assertions are valid on manually verified reference implementations and sufficiently sensitive to injected faulty variants [37, 38].

To quantify defect-detection capability, we first adopt the mutation score, defined as the percentage of generated mutants that are killed by the test suite, where a mutant is considered killed if at least one generated assertion distinguishes its behavior from that of the original implementation. A higher mutation score indicates a stronger ability to detect implementation-level defects. Table 2 presents mutation scores of BioTester on the bioinformatics software benchmark. As the number of generated test cases increases, the mutation score generally improves, indicating that additional test cases provide broader behavioral coverage and stronger sensitivity to faulty variants. Unless otherwise specified, the following analysis focuses on the highest mutation score obtained with the maximum number of generated test cases. Overall, BioTester killed 2072 mutants, achieving an overall mutation score of 0.849. This result shows that the oracles generated by BioTester can effectively identify faulty behaviors introduced by controlled mutations, providing empirical evidence for the correctness and reliability of its oracle generation mechanism. Several datasets achieve or approach a 100% mutation score, such as AlignQC, Cell BLAST, SV-Bay, SVAFotate, cnvkit, kinfin, and modlAMP, indicating that BioTester is broadly applicable to diverse bioinformatics functions.

**Table 2:** Mutation scores under different numbers of generated test cases on bioinformatics software benchmarks.

Dataset	#Mutants	Boolean Replacement			Compare Replacement			Constant Replacement			Function CallArgMutation			Operator Replacement		
		3	5	10	3	5	10	3	5	10	3	5	10	3	5	10
AlignQC	40	0.850	0.925	1.000	0.875	0.950	1.000	0.850	0.925	1.000	0.875	0.950	1.000	0.900	0.950	1.000
Cell BLAST	15	–	–	–	0.800	0.900	1.000	0.800	0.900	1.000	0.850	0.925	1.000	0.850	0.925	1.000
CNVpytor	25	0.850	0.925	1.000	0.850	0.925	1.000	0.400	0.500	0.600	0.850	0.925	1.000	0.875	0.950	1.000
ClassifyCNV	9	–	–	–	0.800	0.900	1.000	0.000	0.000	0.000	0.850	0.925	1.000	–	–	–
metaFlye	81	–	–	–	0.850	0.925	1.000	0.467	0.567	0.667	0.875	0.950	1.000	0.850	0.925	1.000
GSEApy	4	0.750	0.875	1.000	–	–	–	–	–	–	0.750	0.875	1.000	–	–	–
SV-Bay	19	–	–	–	0.850	0.925	1.000	0.850	0.925	1.000	0.875	0.950	1.000	0.850	0.925	1.000
SVAFotate	19	–	–	–	0.850	0.925	1.000	0.850	0.925	1.000	0.875	0.950	1.000	0.850	0.925	1.000
AltAnalyze	1707	0.623	0.731	0.823	0.534	0.628	0.716	0.551	0.646	0.740	0.672	0.771	0.866	0.705	0.812	0.901
Bakta	78	0.850	0.925	1.000	0.850	0.925	1.000	0.750	0.850	0.950	0.559	0.659	0.759	0.875	0.950	1.000
cnvkit	52	0.875	0.950	1.000	0.875	0.950	1.000	0.850	0.925	1.000	0.875	0.950	1.000	0.900	0.950	1.000
DeepCpG	19	0.850	0.925	1.000	0.850	0.925	1.000	0.400	0.500	0.600	0.850	0.925	1.000	0.875	0.950	1.000
gatk-sv	217	0.657	0.757	0.857	0.763	0.863	0.963	0.687	0.787	0.887	0.774	0.874	0.974	0.755	0.855	0.955
gubbins	40	0.000	0.000	0.000	0.467	0.567	0.667	0.400	0.500	0.600	0.657	0.757	0.857	0.850	0.925	1.000
kinfin	21	0.850	0.925	1.000	0.850	0.925	1.000	0.850	0.925	1.000	0.875	0.950	1.000	0.875	0.950	1.000
localCIDER	50	–	–	–	0.850	0.925	1.000	0.850	0.925	1.000	0.000	0.000	0.000	0.875	0.950	1.000
modlAMP	25	–	–	–	0.850	0.925	1.000	0.850	0.925	1.000	0.850	0.925	1.000	0.875	0.950	1.000
snp-pipeline	20	0.850	0.925	1.000	0.850	0.925	1.000	0.600	0.700	0.800	0.850	0.925	1.000	0.875	0.950	1.000
Total	2441	0.655	0.754	0.855	0.598	0.699	0.799	0.592	0.692	0.792	0.682	0.782	0.882	0.735	0.835	0.935

From the perspective of mutation types, BioTester achieves the highest mutation score on OperatorReplacement (0.935), followed by FunctionCallArgMutation (88.20%) and BooleanReplacement (0.855), with CompareReplacement and ConstantReplacement slightly lower (0.799 and 0.792). These differences mainly reflect the relative ease of propagating changes to observable outputs. Mutants that survive are primarily equivalent or near-equivalent, require specific triggering conditions, or involve exception-handling logic that masks internal errors.

Table 3 compares BioTester with several baseline methods on the bioinformatics software benchmark, including CHAT [59–61], Differential Prompting Plus (DPP) [26], Automated Program Repair (APR) [62, 63], and AID [28]. CHAT directly prompts the LLM with the target function and its natural language description to generate test cases with embedded assertion oracles. DPP generates multiple alternative implementations and determines test outcomes based on output consistency across these implementations. APR uses LLMs to repair the target function and then derives assertion oracles from the repaired implementation. AID constructs functionally equivalent variants as auxiliary oracles and checks whether their outputs are consistent with the original function.

**Table 3:** Performance comparison on bioinformatics software benchmarks.

Method	Mutation score	Accuracy	Precision	Recall	Specificity	F1-score	AUC
CHAT	0.574	0.661	0.603	0.574	0.776	0.588	0.675
DPP	0.781	0.821	0.806	0.781	0.873	0.793	0.827
AID	0.646	0.724	0.704	0.646	0.827	0.674	0.736
APR	0.702	0.697	0.621	0.702	0.690	0.659	0.696
**BioTester**	**0.849**	**0.889**	**0.872**	**0.849**	**0.942**	**0.860**	**0.896**

In addition, we introduce metrics to evaluate defect detection capability at the test-case level. Following prior work [28], each generated test case is treated as an independent evaluation instance. A passed test case from a correct unit is defined as a true negative (TN), whereas a correctly failed test case from a faulty unit is defined as a true positive (TP). Conversely, an unexpectedly passed test case from a faulty unit is considered a false negative (FN), and an incorrectly failed test case from a correct unit is labeled as a false positive (FP). To ensure a fair comparison, all baselines are provided with the same set of inputs and use the same underlying LLM. Based on these definitions, we compute standard classification metrics, including accuracy, precision, recall, specificity, F1-score, and AUC.

As shown in Table 3, BioTester achieves the best performance across all metrics. Its overall mutation score reaches 0.849, outperforming DPP (0.781), APR (0.702), AID (0.646), and CHAT (0.574), indicating that BioTester-generated oracles are more effective in detecting faulty behaviors introduced by controlled mutations. At the test-case level, BioTester also obtains the highest accuracy, precision, recall, specificity, F1-score, and AUC, suggesting that it can detect faulty mutants while avoiding incorrectly rejecting correct implementations.

Compared with the baselines, CHAT is less sensitive to subtle behavioral deviations because it directly relies on LLM-generated assertions, while APR and AID are limited by higher false-positive rates or insufficient behavioral constraints. DPP performs closest to BioTester, showing the benefit of differential testing for improving oracle stability. Nevertheless, BioTester consistently outperforms DPP across all metrics, suggesting that retrieval-augmented domain knowledge further improves the reliability of reference implementations and test oracles in bioinformatics software testing.

#### Robustness across LLM backbones and multiple runs

Since the quality of the generated oracle directly determines the effectiveness of subsequent defect detection, it is important to evaluate whether BioTester can consistently produce reliable testing oracles under different LLM backbones and across repeated executions. Therefore, this section investigates the robustness of mutation-testing performance of BioTester to further assess the correctness and reliability of the generated oracles.

Table 4 reports the mutation scores of BioTester on the bioinformatics benchmark using different LLM backbones over five repeated runs. We selected these backbones to cover different model families and capability levels. GPT-4o-mini is the default backbone used in our main experiments because it provides a favorable balance between performance and inference cost. GPT-4o represents a stronger proprietary model, Claude-3.5-Sonnet and Gemini-1.5-Pro are included to evaluate robustness across different commercial LLM families, and Qwen2.5-Coder-32B is selected as a code-oriented open-source backbone.

**Table 4:** Mutation score of BioTester across different LLM backbones and repeated runs.

LLM backbone	Run 1	Run 2	Run 3	Run 4	Run 5	Mean *±* Std.
GPT-4o-mini	0.846	0.852	0.849	0.851	0.847	0.849 *±* 0.002
GPT-4o	0.858	0.861	0.856	0.862	0.859	0.859 *±* 0.002
Claude-3.5-Sonnet	0.852	0.849	0.855	0.851	0.853	0.852 *±* 0.002
Gemini-1.5-Pro	0.841	0.845	0.843	0.846	0.842	0.843 *±* 0.002
Qwen2.5-Coder-32B	0.835	0.839	0.837	0.840	0.836	0.837 *±* 0.002

Overall, BioTester achieves consistently high mutation scores across all LLM backbones. GPT-4o obtains the highest average mutation score of 0.859, followed by Claude-3.5-Sonnet with 0.852 and GPT-4o-mini with 0.849. Gemini-1.5-Pro and Qwen2.5-Coder-32B achieve slightly lower but still competitive scores of 0.843 and 0.837, respectively. The gap between the strongest backbone, GPT-4o, and the default GPT-4o-mini is only 0.010, while the gap between GPT-4o-mini and the lowest-scoring backbone, Qwen2.5-Coder-32B, is 0.012. This small variation indicates that BioTester is not strongly dependent on a specific LLM backbone. The repeated-run results further demonstrate the stability of BioTester. Across all five backbones, the standard deviation is only 0.002, showing that the mutation score remains highly stable under repeated LLM invocations. This suggests that the retrieval-augmented oracle generation process helps constrain the output space of the LLM and reduces the impact of stochastic generation variability.

**Figure 3:**
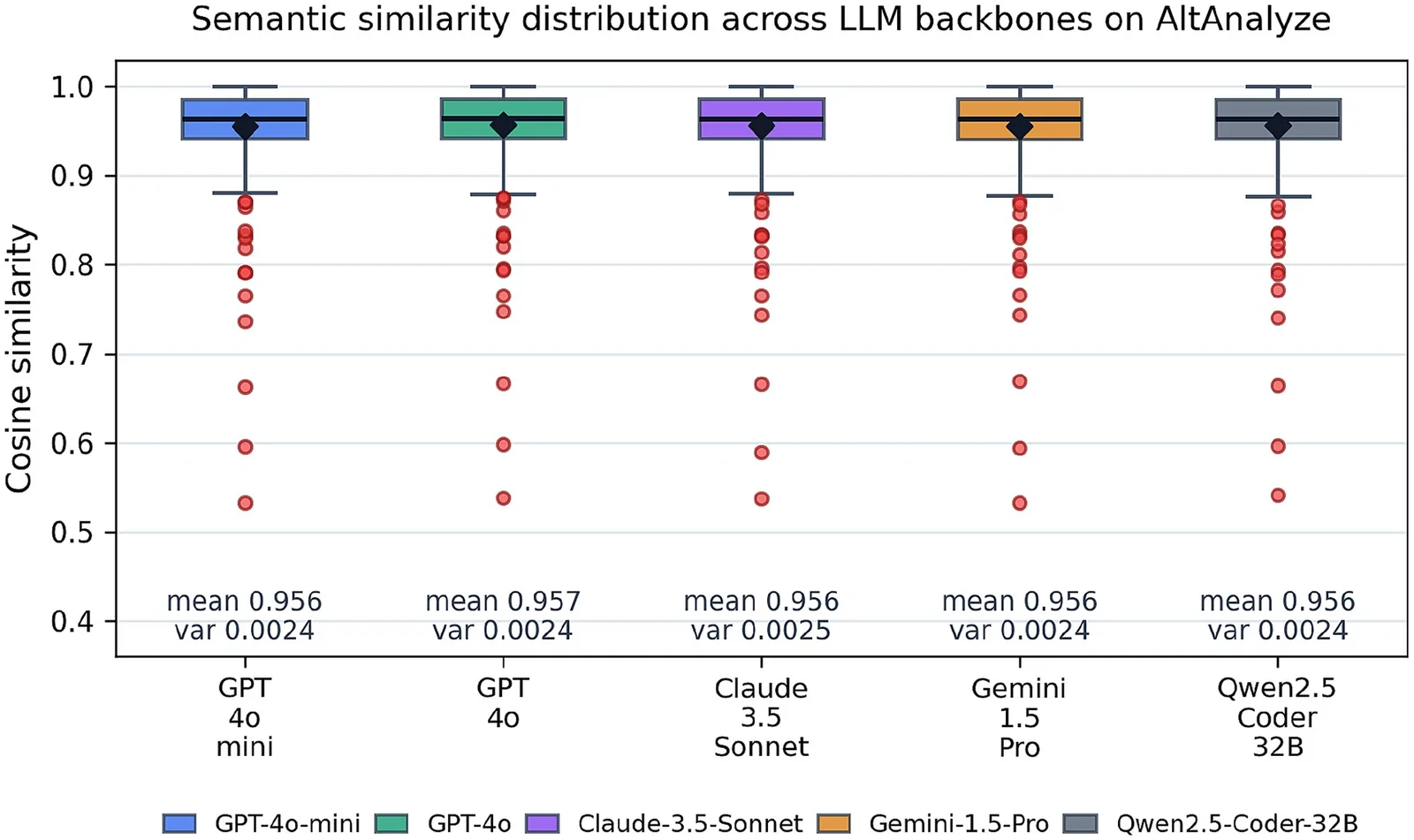
Semantic similarity between original and reference implementations of AltAnalyze samples across LLM backbones.

BioTester further incorporates a semantic consistency checking step in its oracle generation workflow, where CodeT5-base is used to estimate the semantic similarity between the original code and the LLM-generated reference implementation. This similarity score serves as an auxiliary signal for assessing whether the generated implementation preserves the intended functionality. To evaluate the stability of this process across different LLM backbones, we use 364 correct function units from AltAnalyze as examples and compare the semantic similarity distributions under five LLM backbones. As shown in Fig. 3, all five LLM backbones produce consistently high and concentrated similarity distributions. The mean scores are all around 0.956, ranging from 0.9556 to 0.9568, with stable median values around 0.963 and similar variances around 0.0024. The substantial overlap among the distributions indicates that the generated reference implementations remain semantically consistent with the original functions across different LLMs. For the few functions with lower similarity scores, manual inspection suggests that they are mainly caused by implementation-level rewrites rather than clear semantic mismatches.

These results demonstrate that BioTester can consistently generate reliable testing oracles across different LLM backbones and repeated runs, providing further evidence of the correctness and stability of the generated oracles. They also justify our choice of GPT-4o-mini as the default backbone, as it achieves performance comparable with GPT-4o while incurring substantially lower inference cost. Overall, the findings suggest that BioTester’s effectiveness primarily stems from its framework design and domain-aware retrieval augmentation rather than dependence on a particular high-capacity model.

Moreover, we conduct additional experiments to assess the contribution of key modules within BioTester, which are given in the supplementary materials.

### Application of BioTester to real-world bioinformatics software

This section applies BioTester to the collected bioinformatics tools to evaluate its practical utility in real-world software scenarios. In addition to the high-impact tools collected above, we included all open-source Python software associated with articles published in BMC Bioinformatics, Frontiers in Bioinformatics, and PLOS Computational Biology from March to May 2026. This journal-based collection reduces selection bias and evaluates BioTester on both established high-impact tools and recently published software. For each tool, BioTester leveraged its official reference documentation to generate test oracles for all units so as to verify their logical correctness.

#### Overview of defect detection in bioinformatics software

The identified discrepancies were classified into three risk levels, where the manual validation process is given in the supplementary materials. Risk I refers to clear deviations from the intended functional semantics that are likely to indicate implementation defects and require prioritized inspection. Risk II covers behavioral differences arising from alternative implementation strategies that may remain semantically acceptable but could affect reproducibility or cross-version consistency. Risk III mainly involves boundary or exceptional inputs, for which the observed differences may reflect opportunities to improve robustness or input handling. As shown in Table 5, BioTester identified 777 risk functions in 142 of the 194 tools (73.2%), corresponding to an average of 4.01 risk functions per tool. These included 80 Risk I (10.3%), 343 Risk II (44.1%), and 354 Risk III (45.6%) functions. Across the four software cohorts, the proportion of risk software ranged from 58.3% to 78.4%, demonstrating that such discrepancies were consistently identified in both established high-impact tools and recently published software.

**Table 5:** Defects identified by BioTester across different software cohorts. Percentages in parentheses indicate the distribution of risk functions within each cohort.

Software cohort	Total software	Risk software	Rate	Risk functions			Mean
				Risk I	Risk II	Risk III	
High-impact tools	33	22	66.7%	22 (24.7%)	32 (36.0%)	35 (39.3%)	2.70
BMC Bioinformatics	24	15	62.5%	4 (8.9%)	19 (42.2%)	22 (48.9%)	1.88
Frontiers in Bioinformatics	12	7	58.3%	0 (0.0%)	6 (40.0%)	9 (60.0%)	1.25
PLOS Computational Biology	125	98	78.4%	54 (8.6%)	286 (45.5%)	288 (45.9%)	5.02
**Overall**	**194**	**142**	**73.2%**	**80 (10.3%)**	**343 (44.1%)**	**354 (45.6%)**	**4.01**

#### Detailed analysis of implementation-level defects

This section presents an in-depth analysis of three typical defective functions, each selected as a representative example of one risk category. Due to space limitations, analysis of the remaining representative functions is provided in the supplementary materials.

**Listing 6:**
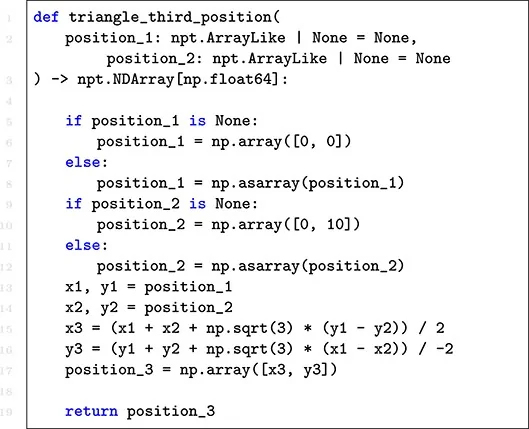
Risk function of **triangle_third_position** in fluopy

In fluopy [64], the **triangle_third_position** function calculates the third vertex of an equilateral triangle from the coordinates of two given vertices. BioTester generated test cases based on this intended geometric property and found that the returned coordinates did not satisfy the test oracle. For example, given *(0,0)* and *(0,10)*, the original implementation returns approximately *(-8.66,-5)*, whereas the corrected expression returns *(-8.66,5)*, which is equidistant from both input vertices. Further inspection revealed a sign error in the calculation of the *y*-coordinate. In Line 16, the denominator -2 negates the entire numerator, incorrectly changing the midpoint term from $(y_{1}+y_{2})/2$ to $-(y_{1}+y_{2})/2$. The negative sign should instead apply only to $(x_{1}-x_{2})$, converting it to $(x_{2}-x_{1})$. Therefore, Line 16 should be corrected to y3 = (y1 + y2 + np.sqrt(3) * (x2 - x1)) / 2. In addition, the function does not check whether the two input vertices coincide, in which case a unique equilateral triangle cannot be determined. This function represents a Risk I discrepancy because incorrect statements directly cause an error in the computational logic and produce results that violate the intended geometric semantics.

Further examination of the original software revealed that it included two test cases for the triangle_third_position function, using *(0,0)* and *(1,0)* in both possible orders. However, both cases satisfy $y_{1}+y_{2}=0$, which masks the erroneous negation of the midpoint term and allows the tests to pass despite the defect. This example demonstrates the importance of automatically generating sufficiently diverse test cases to expose defects that may remain undetected by manually designed tests.

**Listing 7:**
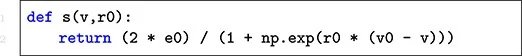
Risk function of **s** in EEG-Dementias

In EEG-Dementias [65], the function **s** transforms the postsynaptic potential into a neuronal firing rate using a sigmoid function. Based on the retrieved article, BioTester identified the following description as the functional specification:

Whole-brain activity at the source level was modeled using a modified version of the Jansen-Rit neural mass model [7,12,30]. The PSPs, *v*, are integrated and transformed into firing rates using a sigmoid function:

\begin{align*} & S(v)=\frac{\zeta_{\max}}{1+\exp\left[-r\left(v-v_{\mathrm{th}}\right)\right]}, \end{align*}

where $\zeta _{\max }$ is the maximal firing rate output, *r* is the slope, and $v_{\mathrm{th}}$ is the voltage threshold.

BioTester instantiated the parameters described in this specification and generated the following reference implementation:

**Listing 8:**
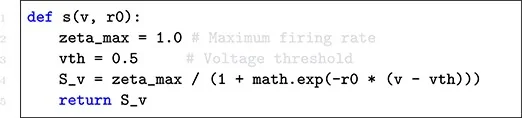
Generation of **s** in EEG-Dementias by BioTester

The original function and the generated function produced different outputs for the generated test cases, rising a Risk II discrepancy caused by inconsistent implementations. Further examination, however, showed that the original implementation is consistent with the classical Jansen–Rit sigmoid model,


\begin{align*} & S(v)=\frac{2e_{0}}{1+\exp\left[r(v_{0}-v)\right]}, \end{align*}

where $2e_{0}$ and $v_{0}$ correspond to the maximum firing rate and voltage threshold, respectively. Thus, the retrieved article expresses the function using $\zeta _{\max }$ and $v_{\mathrm{th}}$ without explicitly relating them to the implementation variables $2e_{0}$ and $v_{0}$, while BioTester assigns concrete values to the parameters when constructing the oracle.

This case therefore represents an alternative-implementation risk rather than a definite implementation defect. More importantly, it demonstrates the value of dynamic testing: the inconsistency becomes observable only by executing both implementations on concrete inputs, whereas static analysis alone may identify the formulas as syntactically and structurally valid without exposing their behavioral differences.

**Listing 9:**
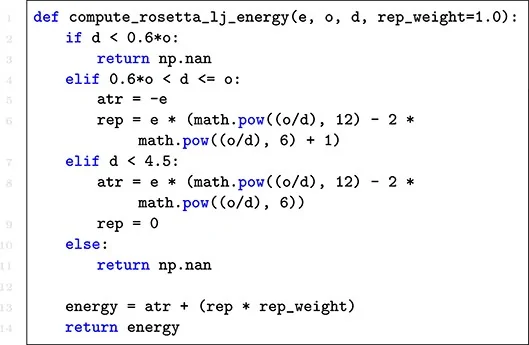
Risk function of **compute_rosetta_lj_energy** in design_guided_optE

In design-guided-optE [66], the compute_rosetta_lj_energy function calculates the Rosetta Lennard–Jones energy according to the distance *d*, well depth *e*, and equilibrium distance *o*. BioTester exposed two potential robustness issues in its boundary and input handling. First, none of the first two conditions covers the boundary *d=0.6o*. When this boundary also satisfies *d<4.5*, execution incorrectly enters the third branch, where the repulsive component is set to zero, rather than the branch intended for short-range interactions. Second, the function does not explicitly validate *d* before using it as a denominator. Consequently, a zero value may cause division by zero or produce an undefined numerical result under certain invalid parameter combinations. BioTester therefore produced an enhanced implementation that explicitly validates the denominator and includes the missing boundary into the secondary branch.

This function was classified as a Risk III discrepancy because the identified issues mainly concern boundary and exceptional inputs. More importantly, by generating sufficiently diverse test cases, BioTester can expose input conditions overlooked by the original implementation and provide an enhanced implementation accordingly. The enhanced version completes the interval definition at *d=0.6o* and explicitly validates the denominator before calculation, thereby preventing unintended branch selection and potential division-by-zero failures. This case demonstrates that comprehensive testing can not only reveal latent risks but also guide defensive improvements that make the original implementation more robust and reliable under unexpected inputs.

## Discussion and conclusion

In this study, we presented BioTester, an open-source automated unit testing framework for bioinformatics software that directly addresses the long-standing oracle problem at the function level. By integrating retrieval-augmented LLMs with differential testing, BioTester enables the automated generation of reliable test oracles and achieves substantial improvements in defect detection performance, yielding a 6.7% increase in F1-score and a 6.9% improvement in AUC in the mutation-based experiment. Through a comprehensive empirical evaluation on real-world bioinformatics tools, we identified previously uncovered subtle defects in most of the analyzed software systems.

As the first framework in bioinformatics that directly addresses the oracle problem at the function level, BioTester remains under active development. Some function units cannot yet be effectively exercised, especially those containing heterogeneous variables with domain-specific data types, which highlights a future direction of synthesizing tests to achieve broad and biologically meaningful execution coverage. Moreover, the current implementation is limited to Python-based tools, indicating a demand of extending BioTester to support multiple programming languages. Although these challenges are largely engineering oriented, further community contributions will be essential for improving interoperability and practical usability.

In summary, BioTester provides a novel and generalizable solution for automated unit-level testing in bioinformatics by effectively combining LLM-based reasoning with differential testing. By enabling systematic defect detection and revealing hidden implementation issues, BioTester supports more rigorous quality assurance practices in bioinformatics and highlights the value of integrating automated unit testing into the development lifecycle to improve software reliability, transparency, and reproducibility.

**Key Points**

- We develop BioTester, an open-source automated unit testing framework that enables systematic quality assurance for bioinformatics software.
- We construct a bioinformatics software defect benchmark by simulating realistic implementation-level discrepancies, providing a controlled basis for evaluating defect-detection methods in this domain.
- We introduce a novel integration of differential testing and retrieval-augmented large language models to address the oracle construction challenge in test case generation, achieving improvements of up to 6.7% increase in F1-score and a 6.9% improvement in AUC over state-of-the-art approaches on defect detection tasks.
- We apply BioTester to real-world bioinformatics software and identify 777 risk functions across 194 evaluated tools. Further analysis shows that correcting discrepancies can lead to measurable changes in downstream results.

## Supplementary Material

Supplementary_material_bbag452

## Data Availability

The source code of BioTester, together with all scripts required to reproduce the experiments, is provided in the access link https://github.com/lianlianlian6/BioTester.
