# Supplementary material for: BioTester: an AI-driven automated testing framework for identifying potential quality risks in bioinformatics software
Source: Brief Bioinform. 2026 Sep 16;27(5):bbag452. doi: 10.1093/bib/bbag452 (PMC13580090; doi:10.1093/bib/bbag452)
Supplement: Supplementary file 1 [file supplementary_material_bbag452.zip › proofing-supplementary_bbag452.pdf]

Supplementary materials

Collection and characterization of bioinformatics software

To understand the current state of software validation in bioinformatics, we first examined the test suites provided by each software package. Specifically, we inspected whether projects included any test files typically expected as part of a software release. This analysis reveals a pronounced lack of effective testing across widely used bioinformatics tools. Among the 33 clinically relevant bioinformatics software projects included in our evaluation, 23 tools (69.7%) show no evidence of any test support, indicating that these software packages may have never undergone systematic validation despite their frequent use and scientific impact. Of the remaining 10 tools (30.3%) that provide tests, most rely on property-based testing to primarily verify pipeline behaviors and only 2 tools (gubbins and localCIDER) provide the expected outputs of partial functional pipelines, where the test scale remains extremely limited relative to the size of the codebase with 7.87%(7/89), 5.88% (4/68), respectively. Notably, several highly influential clinical tools fall into the category of "high impact but low testing". For example, CNVkit, a widely used tool for copy number variation (CNV) detection for both NIPT and cancer screening with over 590 GitHub stars and more than 600 functional units, currently provides only 5 system-level tests, with no function-level verification. GATK-SV, a popular tool for early cancer screening published in Nature and comprising over 1,000 functional units, similarly does not include explicit function-level testing. AltAnalyze, a tool accumulating 398 citations and 104 stars, contains nearly 5,000 function units in its codebase, yet only three simple functional test files are provided. These observations indicate an apparent imbalance between the widespread practical adoption of clinical bioinformatics software and the extent of fine-grained software verification currently in place, suggesting a potentially underappreciated reliability concern in real-world clinical genomic analysis.

Manual validation of detected discrepancies

To systematically distinguish genuine defects from acceptable implementation differences, we performed a manual validation procedure as shown in Figure S1 for all discrepancies detected by BioTester.

Specifically, BioTester first executes generated test cases on both program versions and compares their outputs. Test cases producing identical outputs are labeled as consistent (0), whereas those producing different outputs are labeled as anomalous (1). For each anomalous case, we conduct a detailed manual validation involving source code analysis, documentation review, process information inspection and examination of actual execution results. To reduce subjective bias, the validation process is independently performed by two authors, and any disagreements are resolved through discussion until consensus is reached.

Based on the validation results, we further categorize the detected discrepancies into three risk levels according to their semantic consistency with the intended functionality and their potential impact on downstream analysis:

- **Risk I discrepancies**: Cases where the observed behavior clearly deviates from the intended functional semantics. These discrepancies are more likely to indicate implementation-level defects that may affect analytical results and therefore require prioritized inspection and correction.
- **Risk II discrepancies**: Cases where multiple reasonable implementation strategies may exist for the same functional intent. Although the generated test case exposes behavioral differences between versions, manual inspection suggests that the alternative behaviors can still be semantically acceptable. These discrepancies may not constitute definite defects, but they highlight potential risks for maintainability, reproducibility, or consistency across software versions.
- **Risk III discrepancies**: Cases where the generated test cases involve boundary or exceptional inputs, such as zero values, missing values (NA), or heterogeneous input types. In these situations, the alternative behavior may indicate broader applicability or improved robustness rather than an error in the original implementation. Such discrepancies mainly suggest opportunities for further refinement or defensive handling of edge cases.

Figure S2 summarizes the distribution of detected potential risk units across the assessed bioinformatics software. Based on the right pie chart, we observed that 22 out of the 33 evaluated bioinformatics software packages (66.7%) contain at least one detected anomalous functional unit. As the left bar chart shown, the number of buggy units per tool ranging from 1 to 17, resulting in 89 buggy units in total with an average number of 4 in each tool. The widespread presence of anomalous units across tools of different scales, application domains, and publication venues indicates that implementation-level defects

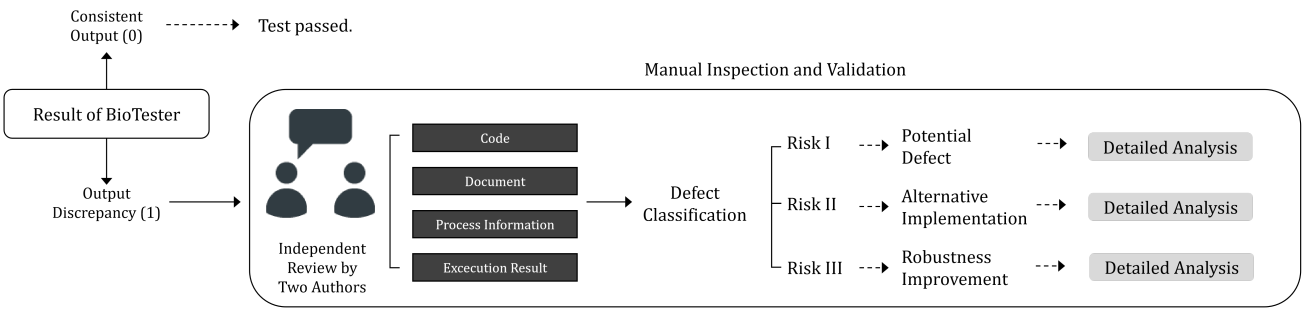

Fig. S1. Systematic validation and classification of detected discrepancies.

Table S1. Clinically relevant bioinformatics software included in the quality assessment. All metadata (e.g., latest update, GitHub stars, and citation counts) were collected as of December 15, 2025.

Domain	Task	Software	Latest update	Stars	Journal	Citations	Modules	Tests
Sequence and Genome Analysis	Read alignment	AlignQC	2018	49	F1000Research	589	143	0
		bwa-meth	2025	153	arXiv	206	68	0
	Genome assembly	modlAMP	2025	61	Bioinformatics	210	39	18
		metaFlye	2025	884	Nature Methods	949	178	0
		hecatomb	2025	59	GigaScience	16	28	0
	Annotation and interpretation	SVAFotate	2024	44	BMC Bioinformatics	22	23	0
		Bakta	2025	578	Microbial Genomics	1128	198	12
Genome Variant Analysis	CNV detection	ClassifyCNV	2023	69	Scientific Reports	79	20	0
		cnvkit	2025	590	PLOS Computational Biology	2096	613	5
		CNVpytor	2025	209	GigaScience	108	314	0
		cnvScan	2016	25	BMC Genomics	27	17	0
		PEcnv	2022	20	Briefings in Bioinformatics	14	548	0
		LMADCNV	2024	0	IEEE TCBB	0	9	0
		waveCNV	2022	2	Mathematics	3	34	0
		EMcnv	2025	0	Briefings in Bioinformatics	0	769	0
		ZIPcnv	2025	8	Bioinformatics	1	21	0
		CNV-FB	2023	0	J. Bioinformatics Comput. Biol.	0	23	0
		ECOLE	2024	26	Nature Communications	12	20	0
		WisecondorX	2025	108	Nucleic Acids Research	152	47	0
	SV detection	SV-Bay	2019	13	Bioinformatics	47	44	0
		gatk-sv	2025	196	Nature	959	1076	0
Evolutionary Bioinformatics	Phylogenetic analysis	snp-pipeline	2023	64	PeerJ Computer Science	272	99	2
		gubbins	2025	207	Nucleic Acids Research	2641	89	7
		PangenomeX	2025	3	Briefings in Bioinformatics	0	46	0
Functional Genomics and Systems Biology	Enrichment analysis	GSEApy	2025	679	Bioinformatics	886	71	0
	Transcriptomic and single-cell analysis	Cyclum	2021	23	Nature Communications	50	42	0
		Cell BLAST	2024	94	Nature Communications	143	79	4
		novoSpaRc	2023	131	Nature Protocols	125	43	0
		AltAnalyze	2022	104	Nucleic acids research	398	4992	3
	Epigenomics analysis	DeepCpG	2019	153	Genome biology	714	169	6
Protein and Structural Bioinformatics	Protein sequence and functional analysis	kinfin	2024	31	G3	100	56	0
		localCIDER	2023	25	Biophysical Journal	552	68	4
		alphafold	2025	14.6k	Nature	43672	404	8

are a common and systemic phenomenon in bioinformatics software, rather than isolated cases.

Component-wise Analysis of BioTester

Our evaluation focuses on two aspects that together evaluate the effectiveness of key components in BioTester. First, BioTester relies on a domain-adapted LLM to extract precise function intentions for subsequent knowledge retrieval, making the quality of intention summarization a critical component. To assess this module, we measure its performance using widely adopted natural language generation metrics before and after fine-tuning. Second, BioTester employs a retrieval-augmented multi-stage reasoning mechanism to generate testing oracles and identify implementation defects. While domain-specific knowledge retrieval provides valuable contextual information, relevant knowledge may not always be available or sufficiently informative in practice. Therefore, it is important to assess whether the underlying reasoning mechanism remains effective even in the absence of retrieval augmentation. To this end, we evaluate the defect-identification capability of the reasoning framework on publicly available defect datasets, where the contribution of the reasoning process can be examined.

For intention generation evaluation, we evaluated our fine-tuned model using three datasets. The primary bioinformatics benchmark (BioTester, 420 samples) consists of collected function code snippets and corresponding summaries from tools

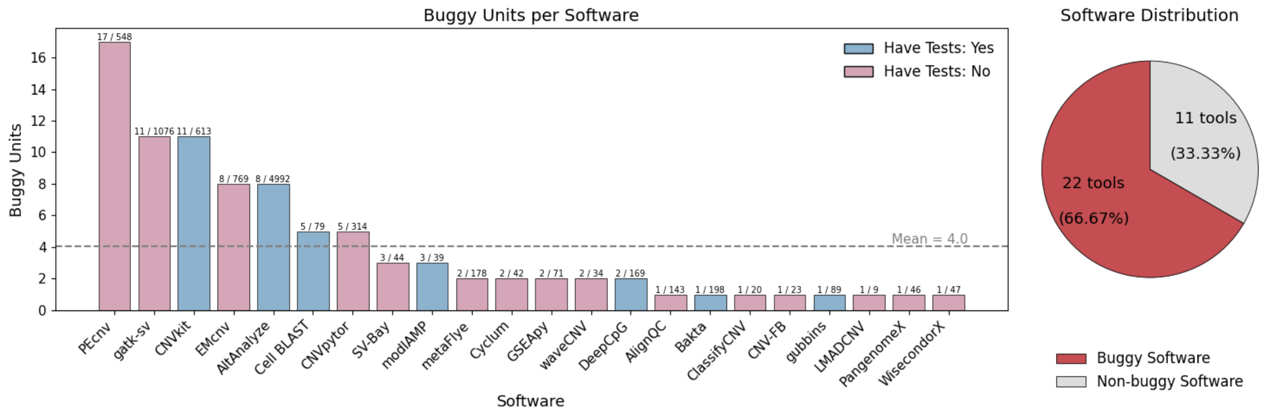

Fig. S2. Summary of detected risk software by BioTester.

such as CNVkit, CNVpytor, inferCNV, and Scanpy, assessing domain-specific summarization performance. To examine cross-domain generalization, 257 samples from MBPP and 300 samples pairs from the Python subset of CodeSearchNet are also included. For model fine-tuning, 70% of each dataset was used for training and 30% for evaluation. We evaluated the quality of generated summaries using widely adopted natural language generation metrics [67], including BLEU, which measures n-gram overlap between generated and reference summaries; ROUGE-1, assessing unigram overlap for basic content similarity; ROUGE-2, capturing bigram overlap to reflect fluency and local coherence; and ROUGE-L, which evaluates the longest common subsequence to quantify overall sequence similarity. Together, these metrics provide a quantitative assessment of how closely the generated summaries align with human-written descriptions, both syntactically and semantically.

Table S2. Performance comparison of base and trained models on intention generation across different datasets.

Dataset	Model	BLEU-4	ROUGE-1	ROUGE-2	ROUGE-L
BioTester	Base	4.502	9.913	0.988	4.977
	Fine-tuned	**33.149**	**31.143**	**15.791**	**38.422**
MBPP	Base	15.931	27.362	7.279	16.300
	Fine-tuned	**23.292**	**39.052**	**25.242**	**43.942**
CodeSearchNet	Base	5.155	10.237	1.396	4.988
	Fine-tuned	**29.076**	**35.972**	**16.668**	**33.532**

As shown in Table S2, the fine-tuned model achieves the largest performance gain on the BioTester dataset. BLEU-4, which measures the overlap of higher-order n-grams (4-grams in this case) between the generated summaries and reference descriptions, reflecting the accuracy of local semantic patterns, shows a dramatic increase from 4.50 to 33.15. BLEU-4 scores typically range from 0 to 100, with scores under 5 generally indicating poor performance in generating accurate summaries, while scores above 30 suggest high-quality output. The significant increase in BLEU-4 highlights that the base model struggles to capture the complex semantic structure of bioinformatics code, while fine-tuning enables the model to produce summaries that closely match reference descriptions, achieving strong performance in this domain-specific task. ROUGE-L, which evaluates the longest common subsequence between the generated and reference summaries, capturing global semantic consistency, improves from 4.98 to 38.42. ROUGE-L is a commonly used metric in summarization tasks, with values typically ranging from 0 to 100. A ROUGE-L score below 10 is generally considered poor, while scores between 20 and 35 are considered good, and values above 35 are considered excellent. The large increase in ROUGE-L indicates that fine-tuning significantly improves the model's ability to produce summaries that preserve the global structure and semantic content of bioinformatics code. ROUGE-2, which evaluates bigram overlap, measures the accuracy of key phrase generation and shows a notable increase from 7.28 to 25.24. ROUGE-2 typically ranges from 0 to 100, with scores below 5 indicating a lack of important phrase matches, and values between 15 and 25 signifying good performance. A score above 25 is considered excellent for summarization tasks. The improvement in ROUGE-2 suggests that fine-tuning enhances the model's ability to generate more informative and precise summaries, improving the identification of key concepts in the bioinformatics code.

For the MBPP dataset, a collection of small Python programming tasks, the fine-tuned model again outperforms the base model. The BLEU-4 score increases from 15.93 to 23.29, and ROUGE-2 nearly triples from 7.28 to 25.24. These results demonstrate that fine-tuning not only improves the model's performance on domain-specific tasks but also enhances its ability to generate more structured and informative summaries for general-purpose programming tasks. Finally, for the CodeSearchNet dataset, which contains real-world functions from diverse GitHub repositories, the fine-tuned model achieves substantial improvements. The BLEU-4 score rises from 5.16 to 29.08, ROUGE-1 increases from 10.24 to 35.97, and ROUGE-L improves from 4.99 to 33.53. ROUGE-1, which measures unigram overlap, typically ranges from 20 to 45, with values above 30 indicating high quality in capturing key semantic tokens. The large increase in ROUGE-1 indicates that fine-tuning significantly enhances the model's ability to generate summaries that capture the essential function semantics of code. These results further support the notion that domain-adapted fine-tuning improves the model's ability to understand and summarize code effectively, even when applied to real-world datasets with varying degrees of complexity. Overall, these results indicate that fine-tuning enhances the model's ability to generate accurate, informative, and semantically rich summaries across both domain-specific and general programming tasks. This demonstrates the effectiveness of fine-tuning in improving the model's performance on specialized tasks like bioinformatics code summarization, as well as on general code summarization tasks such as those in MBPP and CodeSearchNet.

Table S3. Performance comparison on the QuixBugs dataset.

Method	Accuracy	Precision	Recall	Specificity	F1-score	AUC
CHAT	0.684	0.562	0.844	0.583	0.674	0.713
DPP	0.785	0.801	0.734	0.832	0.766	0.783
AID	0.869	0.845	0.895	0.731	0.869	0.813
APR	0.744	0.591	**1.000**	0.593	0.743	0.796
**BioTester**	**0.910**	**0.870**	0.949	**0.875**	**0.908**	**0.912**

For the oracle generation evaluation, we employed two widely-used benchmark defect detection datasets including QuixBugs (100 samples) [68] and TrickyBugs (1549 samples) [69]. QuixBugs contains 50 buggy programs, each paired with a corrected version, resulting in 100 program instances. And TrickyBugs is a large-scale dataset containing 3043 buggy plausible programs sourced from human-written submissions of 324 real-world programming competition problems. From this dataset, we selected 703 verified correct programs and their 846 corresponding buggy implementations, yielding 1,549 program instances. We compared BioTester against the mentioned representative unit testing baselines with binary classification accuracy metrics.

Table S3 shows the detection accuracy of BioTester and baselines across the QuixBugs dataset. BioTester demonstrates superior performance across all six evaluation metrics. It achieves the highest accuracy at 0.910, significantly surpassing the second-best method AID (0.869), indicating a strong overall classification ability. With a value of 0.87, BioTester maintains the highest precision, showing its effectiveness in reducing false positives when identifying buggy programs. BioTester also achieves the highest recall at 0.949, indicating its ability to identify nearly all buggy programs, outperforming DPP (0.734) and CHAT (0.844). BioTester records 0.875 for

specificity, reflecting a better ability to correctly identify correct programs with fewer false alarms. With the F1-score of 0.908, BioTester shows the best balance between precision and recall, outperforming all other methods. Besides, BioTester reaches an AUC of 0.912, highlighting its strong overall classification robustness. It is also worth noting that while APR achieves a perfect recall of 1.0, its precision (0.591) and specificity (0.593) are much lower, indicating that although it detects all buggy programs, it generates a high number of false positive test cases making it impractical for real-world use.

Table S4. Performance comparison on the TrickyBugs dataset.

Method	Accuracy	Precision	Recall	Specificity	F1-score	AUC
CHAT	0.645	0.703	0.604	0.697	0.678	0.649
DPP	0.699	0.659	0.702	0.696	0.680	0.699
AID	0.683	0.665	0.819	0.529	0.734	0.674
APR	0.548	0.534	**0.943**	0.132	0.682	0.537
**BioTester**	**0.748**	**0.748**	0.815	**0.733**	**0.780**	**0.774**

On the more challenging TrickyBugs dataset, BioTester continues to demonstrate robust and balanced performance as shown in Table S4. BioTester achieves the highest accuracy (0.748), outperforming DPP (0.699) and AID (0.683). Also at 0.748 of precision, showing consistent performance in correctly identifying buggy programs with fewer false positives. BioTester achieves 0.815 recall, slightly lower than AID (0.819), but much higher than CHAT (0.604) and DPP (0.702). Compared to the highest recall of APR (0.943), BioTester avoids the drop in precision, where the precision of APR is only 0.534. With specificity At 0.733, BioTester again surpasses AID (0.529) and APR (0.132), indicating better performance in recognizing correct programs. BioTester achieves 0.780 for F1-score, the best among all methods, indicating balanced and reliable performance. With an AUC of 0.774, BioTester maintains strong classification ability, outperforming all baselines.

Across both datasets, BioTester consistently outperforms all baseline methods, demonstrating the effectiveness of its reasoning framework for defect identification. The advantage is particularly pronounced on TrickyBugs, where bug patterns are more diverse and program structures are more complex. Importantly, these experiments exclude domain-specific knowledge retrieval and evaluate only the underlying reasoning mechanism. Despite the absence of retrieval augmentation, BioTester maintains strong performance across both datasets, indicating that its chain-of-thought reasoning process possesses robust intrinsic defect-detection capabilities. This finding suggests that the reasoning framework itself forms a reliable basis for software defect identification, while retrieval augmentation further enhances its performance when relevant domain knowledge is available.

Additional representative defective functions with detailed analysis

In CNVkit, the **FDRThres** function aims to compute a significance threshold T for a set of test statistics $x_1, x_2, \ldots, x_M$ using the Benjamini-Hochberg (BH) procedure, which controls the false discovery rate (FDR) at level q [70]. The code is as follows:

```
1  def FDRThres(x, q, stdev):
2      """False discovery rate (FDR) threshold."""
3      M = len(x)
4      if M < 2:
5          return 0
6  
7      m = np.arange(1, M + 1) / M
8      x_sorted = np.sort(np.abs(x))[::-1]
9      p = 2 * (1 - stats.norm.cdf(x_sorted, stdev))
10 
11     # Get the largest index for which p <= m*q
12     indices = np.nonzero(p <= m * q)[0]
13 
14     if len(indices):
15         T = x_sorted[indices[-1]]
16     else:
17         T = x_sorted[0] + 1e-16
18     return T
```

Listing 10. Buggy function of **FDRThres** in CNVkit

The calculation steps are supposed to be: Firstly, compute p-values from test statistics x_i assumed to follow a normal distribution:

$$p_i = 2 \cdot (1 - \Phi_{\mu,\sigma}(|x_i|))$$

where $\Phi_{\mu,\sigma}$ is the CDF of the normal distribution with mean μ and standard deviation σ. Secondly, sort p-values $p_{(1)} \leq p_{(2)} \leq \cdots \leq p_{(M)}$, and find the largest k such that:

$$p_{(k)} \leq \frac{k}{M} \cdot q$$

Finally, set the threshold $T = |x|_{(k)}$.

In the CNVkit implementation, $\Phi_{\mu,\sigma}(|x_i|)$ is computed in line 9 using

```
stats.norm.cdf(x_sorted, stdev)
```

whose default function signature is `stats.norm.cdf(x, loc=0, scale=1)`. The second parameter `loc` specifies the mean μ of the normal distribution, while the third parameter `scale` corresponds to the standard deviation σ. According to the context of the function `FDRThres`, the variable `stdev` is intended to represent the standard deviation but is mistakenly passed as the second positional argument `loc` rather than `scale` in the current implementation. It results in the calculation of p_i as:

$$p_i = 2 \cdot (1 - \Phi_{\mu=\texttt{stdev},\sigma=1}(|x\_sorted_i|))$$

instead of the correct value:

$$p_i = 2 \cdot (1 - \Phi_{\mu=0,\sigma=\texttt{stdev}}(|x\_sorted_i|))$$

It is a typical example of a bug caused by incorrect argument passing. And the correct code is supposed to be:

```
stats.norm.cdf(x_sorted, scale = stdev)
```

Although this subtle bug leads to incorrect p-values, its practical impact on the CNV detection results is relatively limited. The effect is somewhat mitigated by the empirical nature of the threshold selection process and the monotonicity of the normal CDF, which can still preserve the ordering of values under certain conditions.

To further understand the impact of the bug, we conduct a comparative survey on the behaviors of the buggy and corrected `FDRThres` function. We simulate 100 random variables drawn from a normal distribution with mean 0 and standard deviation 2. These values, denoted as x_{sorted}, represent sorted

statistics used for FDR thresholding. As for the parameter `stdev`, by analyzing the invocation logic throughout the CNVkit codebase, it is found that `stdev` is derived from two primary sources:

- **Empirical estimation:** In several cases, `stdev` is computed from the observed values using the Median Absolute Deviation (MAD), which is then converted to the standard deviation of a normal distribution via a scaling factor. Specifically:

$$\texttt{stdev} = \frac{\text{MAD}}{\Phi^{-1}(0.75)} \approx \text{MAD} \times 1.4826$$

- **Fixed assignment:** In some contexts, `stdev` is simply assigned a constant value of 1, likely as a default or fallback setting.

And the parameter `q`, which controls the false discovery rate threshold, typically falls within the range: $0 < q < 0.5$, and $q \in \{10^{-2}, 10^{-3}, 10^{-4}\}$ are commonly used. Then we compare outcomes of the original call `stats.norm.cdf(x, loc=stdev)` and the correct call `cdf(x, loc=0, scale=stdev)` under different `stdev` and `q` settings. As shown in Figure S3, we visualize the p-values computed under both the original (red) and corrected (blue) implementations, along with the threshold line mq (gray dashed) for different $q \in \{0.05, 0.01, 0.001\}$. The yellow vertical line indicates the selected index and threshold T from the original implementation; the green line represents the corrected result.

Under the setting where `stdev` is fixed to 1, we observe that the original implementation of `FDRThres` erroneously returns index values of 2, 0, and -1 for q set to 0.05, 0.01, and 0.001, respectively, with corresponding thresholds $T = 4.48$, $T = 5.11$, and $T = 5.11$. In contrast, the corrected implementation returns index values of 20, 12, and 2 with thresholds $T = 2.84$, $T = 3.23$, and $T = 4.48$ under the same q values. When `stdev` is derived from MAD, the original implementation again yields $T = 4.48$, $T = 5.11$, and $T = 5.11$ for $q = 0.05$, 0.01, and 0.001, respectively. The corrected version, meanwhile, produces thresholds $T = 3.53$, $T = 5.11$, and $T = 5.11$ with corresponding index values of 8, 0, and -1.

These results demonstrate that the numerical difference in selected thresholds T between the erroneous and corrected versions is relatively small in magnitude. Furthermore, as `q` decreases, both implementations produce increasingly similar index values leading to similar thresholds. Importantly, CNVkit explicitly recommends using $q \in \{10^{-2}, 10^{-3}, 10^{-4}\}$ in practice, where the resulting index values in both implementations are often concentrated near -1, 0, or 1. Since the fallback mechanism in the `FDRThres` function returns index 0 when no p-values meet the thresholding criteria with index -1, and the surrounding indices tend to correspond to similar or slightly lower T, the output remains relatively stable. This helps explain why the incorrect implementation has minimal impact on the correctness of the logic in the most cases. Nevertheless, the bug may subtly degrade the statistical validity of the FDR thresholding and should be corrected for consistency and robustness.

It should be noted that this issue was subsequently fixed by the software developers on February 6, 2026. BioTester identified this issue during the analysis of CNVkit Version 0.9.11, which was released on April 14, 2024, and our experiments had already been completed in 2025, prior to the official fix. Therefore, the later correction by the developers provides independent evidence that the discrepancy reported by BioTester corresponded to a genuine defect.

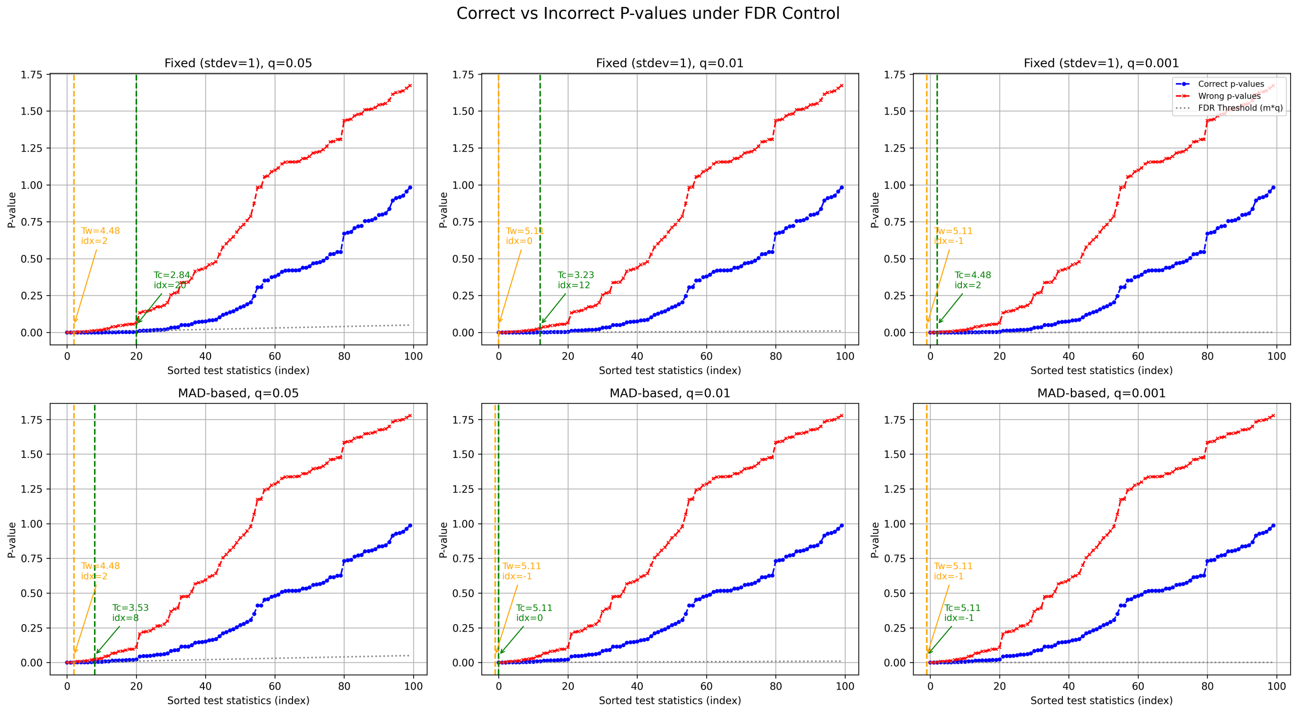

Fig. S3. Outcomes under correct and incorrect `FDRThres` function.

```
1  def in_which(x, ys, ye):
2      n = len(ys)
3      m = len(x)
4      rv = np.empty(m, dtype=np.int)
5      rv.fill(-1)
6      i = 0
7      j = 0
8      while i < n and j < m:
9          while j < m and x[j] <= ye[i]:
10             if x[j] >= ys[i]:
11                 rv[j] = i
12             j += 1
13         i += 1
14     return rv
```

Listing 11. Buggy function of **in_which** in DeepCpG

In DeepCpG, the function **in_which** is designed to return, for each position in `x`, the index of the interval `[ys[j], ye[j]]` that contains it, or `-1` if the position does not overlap with any interval. The intervals are assumed to be non-overlapping and sorted by their start positions. It initializes the return array using `np.empty(m, dtype=np.int)` in line 4 and then fills it with `-1`. However, `np.int` is a deprecated NumPy type alias and has been removed in recent NumPy versions. As a result, passing `np.int` as the `dtype` parameter to `np.empty` may cause the function to fail at runtime before the interval-search logic is executed. This represents a parameter passing error, where an invalid or no-longer-supported argument is passed to a library API. In the BioTester-generated variant, this initialization is replaced by `rv = [-1] * m`, which avoids passing the deprecated NumPy type alias and enables the function to execute successfully.

And another representative buggy implementation caused by incorrect parameter passing is the **breakpoint_select** function in PEcnv.

```
1  def breakpoint_select(dfArr, sp, upline, dowline):
2      index = 0
3      breakpoints = []
4      for i in dfArr.ewm(span=sp).mean():
5          if i > upline or i < dowline:
6              breakpoints.append(index)
7          index += 1
8      return breakpoints
```

Listing 12. Buggy function of **breakpoint_select** in PEcnv

The **breakpoint_select** function identifies potential breakpoints in a numerical sequence by applying an Exponentially Weighted Moving Average (EWMA) smoothing [71, 72]. Specifically, it scans the smoothed series and selects indices where the EWMA values exceed a predefined upper threshold or fall below a lower threshold. This method helps detect deviations or shifts in the trend of the input data sequence, which may correspond to structural changes or outliers. In order to implement the EWMA concept described in the literature:

$$\text{EWMA}_t = \alpha \cdot x_t + (1 - \alpha) \cdot \text{EWMA}_{t-1}$$

`dfArr.ewm(span=sp).mean()` in line 4 is used to compute the EWMA statistic in the code, which is a function provided by the **pandas** library [73]. Here, the parameter `span` controls the degree of smoothing, and the smoothing factor α is given by: $\alpha = \frac{2}{\text{span}+1}$. This is the recursive formulation, updating the current EWMA based on the latest observation and the previous EWMA, which is efficient and widely used in time series analysis. However, `dfArr.ewm` function has the default argument setting with `dfArr.ewm(span=sp, adjust=True)`, leading to the EWMA statistic computed as:

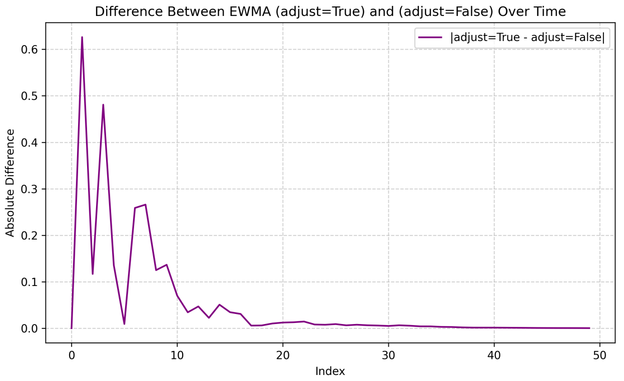

Fig. S4. Difference between correct and incorrect `breakpoint_select` function.

$$\text{EWMA}_t = \frac{\sum_{i=0}^{t} (1-\alpha)^i \cdot x_{t-i}}{\sum_{i=0}^{t} (1-\alpha)^i}$$

This formulation assigns exponentially decreasing weights to past observations and ensures proper normalization, which is mathematically equivalent to the recursive one but differs in initialization and weight scaling, especially for early values in the time series. It provides an unbiased estimate by reweighting the entire history, whereas the recursive formulation offers a computationally efficient approach corresponding to `adjust=False` in the `dfArr.ewm` function. According to the literature, the corrected code is supposed to be `dfArr.ewm(span=sp, adjust=False)`. In conclusion, it can be considered as a bug caused by configuration-driven logic changes.

To investigate the numerical differences between two formulations of EWMA, we simulate a time series by adding Gaussian noise (mean 0, std 3) to a linearly increasing trend from 0 to 10. And Figure S4 shows the absolute difference between the two EWMA statistics with $span = 10$. It can be seen that the most notable discrepancies between the two formulations occur at the beginning, and the two versions tend to converge and produce similar results as the number of observations increases over time. The diff series is expected to show small values overall and converges fast especially when the time series length is substantially larger than the `span`. Since CNV base sequences are typically much longer than the specified `span` value, the initial discrepancies introduced by the EWMA smoothing are limited in scope and considered acceptable in practice.

For both the BH and EWMA procedures are classical statistical approaches for controlling the false discovery rate and processing sequential data, respectively, which are widely adopted in variant detection software, we observed that similar incorrect and inconsistent implementations of this function in multiple tools, including PEcnv, EMcnv, and GSEApy.

For the boundary handling issue, the `EWMA_SEG` function in PEcnv aims to merge adjacent breakpoint positions into continuous segments. It takes a list of selected breakpoints as input and whenever the distance between two consecutive breakpoints is less than a predefined threshold (`edgeSize`), it

merges them into a single segment extended on both sides by `edgeSize` bases.

```
1  def EWMA_SEG(breakpointSelects):
2      segs = []
3      start = breakpointSelects[0]
4      itm = 1
5      edgeSize = 20
6      for i in range(len(breakpointSelects)):
7          if i + 1 < len(breakpointSelects):
8              if itm == 1:
9                  start = breakpointSelects[i]
10             itm += 1
11             if breakpointSelects[i + 1] -
                    breakpointSelects[i] < edgeSize:
12                 if i + 1 == len(breakpointSelects) - 1:
13                     if start - edgeSize > 0:
14                         segs.append([start - edgeSize,
                               breakpointSelects[i + 1] +
                               edgeSize])
15                     else:
16                         segs.append([start,
                               breakpointSelects[i + 1]])
17             elif breakpointSelects[i + 1] -
                    breakpointSelects[i] > edgeSize:
18                 end = breakpointSelects[i]
19                 if start - edgeSize < 0:
20                     segs.append([start, end + edgeSize])
21                 else:
22                     segs.append([start - edgeSize, end +
                           edgeSize])
23                 itm = 1
24     return segs
```

Listing 13. Buggy function of **EWMA_SEG** in PEcnv

The key logic iterates over all breakpoint positions, using a counter variable `itm` to track whether a new segment should be started. If the next breakpoint is close to the current one (i.e., within `edgeSize`), the segment continues; otherwise, the current segment is closed and a new one is started. However, the original implementation contains a limitation in its loop termination logic. When the last pair of breakpoints is encountered and their distance exceeds `edgeSize`, the final breakpoint is not merged into any segment and thus gets ignored. For example,

- `EWMA_SEG([100, 115, 130, 300, 315])` correctly returns `[[80, 150], [280, 335]]`, since the first three breakpoints form a dense cluster and the last two are also close enough to be merged.
- `EWMA_SEG([100, 105, 112, 180])` incorrectly returns `[[80, 132]]`. The last breakpoint 180 is omitted because its distance to the preceding one (112) is greater than `edgeSize`. The correct output should be `[[80, 132], [160, 200]]`.
- `EWMA_SEG([10, 100, 300])` returns `[[10, 30], [80, 120]]`, omitting the final breakpoint 300. The expected result is `[[10, 30], [80, 120], [280, 320]]`.

This behavior occurs because the logic for handling isolated breakpoints at the end of the list is only triggered when the last two breakpoints are within `edgeSize`. If not, the final point is never emitted into a segment. Because the main merging logic only executes when `i + 1 < len(breakpointSelects)` as line 7 shown. As a result, the last breakpoint is excluded from any processing if it is not part of a merged region. Besides, there is no `else` branch to handle isolated final breakpoints after the main loop finishes. It can be considered as a bug caused by improper edge-case handling. To resolve this, BioTester produced the correct code by adding an additional `else` branch corresponding to line 7 to explicitly handle the final breakpoint when it remains unprocessed. The fix can be described by the following code:

```
else:
    if itm == 1:
        if breakpointSelects[i] - edgeSize > 0:
            segs.append([breakpointSelects[i] -
                edgeSize, breakpointSelects[i] +
                edgeSize])
        else:
            segs.append([breakpointSelects[i],
                breakpointSelects[i] + edgeSize])
```

Listing 14. Fix for unprocessed final breakpoint

This ensures that all breakpoints, including isolated ones at the end of the list, are properly converted into segments. We further analyzed the usage of `EWMA_SEG` function in PEcnv and found that the subsequent processing involves smoothing the sequence segments based on their classification into CNV candidate regions and normal regions. Specifically, the output of `EWMA_SEG` function is CNV candidate regions, which are then smoothed using a fixed small window of size $2 \times wing + 1$, whereas normal regions are smoothed using a larger dynamic window of size $winSize \times wing + 1$. In the original buggy program, some CNV candidate regions were mistakenly omitted and thus processed with the dynamic window instead, leading to computational bias. To demonstrate the smoothing bias caused by incorrect region classification in CNV analysis, we simulated a one-dimensional CNV-like signal of length 250. The signal consists of three segments:

- positions 0–99: normal region sampled from $\mathcal{N}(2.0, 0.1)$;
- positions 100–149: a CNV gain region sampled from $\mathcal{N}(3.0, 0.1)$;
- positions 150–249: normal region again sampled from $\mathcal{N}(2.0, 0.1)$.

The CNV event was defined to lie between positions $[100, 150)$. With the fixed `wing` size of 2, four different smoothing window settings corresponding to `winSize` $\in \{1, 2, 3, 4\}$ are evaluated. In Figure S5, each subplot compares the raw signal (gray), correct smoothing (solid blue line using fixed small window), and incorrect smoothing (dashed orange line using larger dynamic window) within a known CNV gain segment (shaded area). When `winSize` is less than 2, the smoothed curve exhibits greater fluctuation compared to the original signal, with a mean squared error (MSE) of 0.00541. In contrast, when `winSize` exceeds 2, the smoothed curve becomes increasingly flat as the window size grows, with MSE values of 0.00296 and 0.00277, indicating that the CNV signal intensity is attenuated. Nevertheless, all issues remain on the order of 10^{-3}, and such differences have negligible impact on the final detection results.

For the statement misalignment issue,, we found a buggy function **scale_sample** in WisecondorX, which is designed to rescale genomic signal bins from a finer resolution to a coarser one by aggregating adjacent bins. Given an input signal array and two bin sizes, `from_size` and `to_size`, the function sums every `to_size/from_size` consecutive bins to construct a new signal representation. Such bin aggregation operations are fundamental to CNV detection pipelines, where normalized

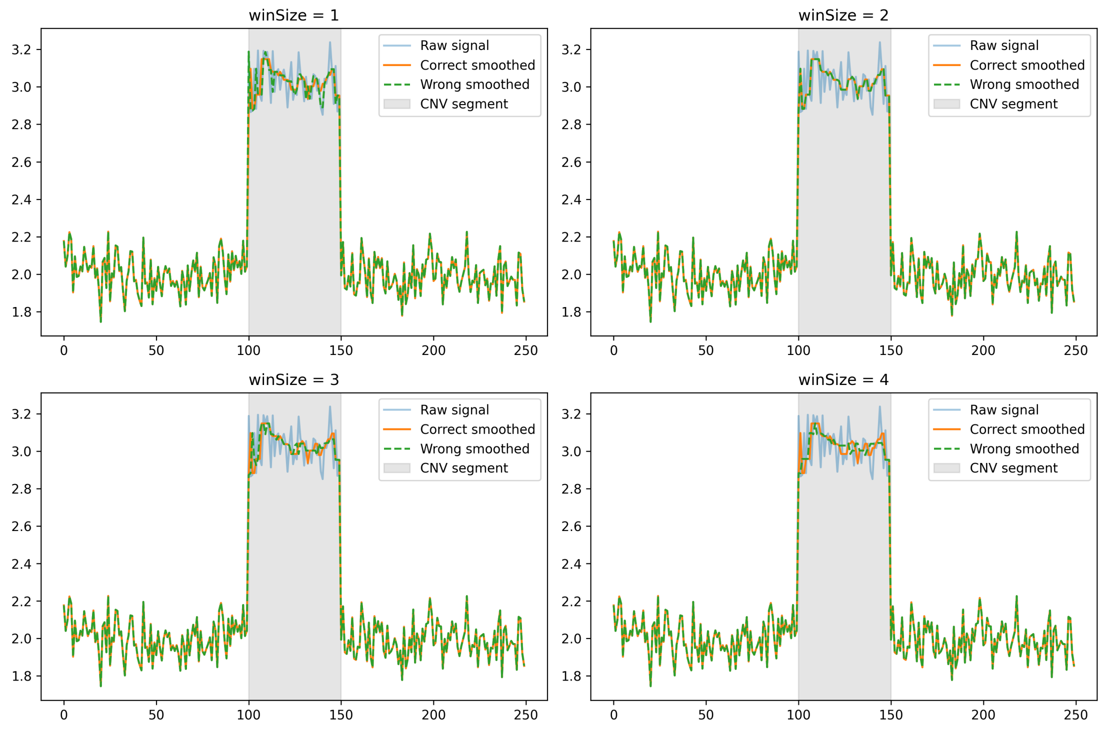

Fig. S5. Difference between correct and incorrect smoothing window size.

coverage or log-ratio signals are routinely summarized across genomic windows to reduce noise and facilitate downstream statistical analysis. The source code is shown as follows:

```
1  def scale_sample(sample, from_size, to_size):
2      if not to_size or from_size == to_size:
3          return sample
4
5      if to_size == 0 or from_size == 0 or to_size <
           from_size or to_size % from_size > 0:
6          logging.critical(
7              "Impossible binsize scaling requested: {}
                   to {}".format(
8                  int(from_size), int(to_size)
9              )
10         )
11         sys.exit()
12
13     return_sample = dict()
14     scale = to_size / from_size
15     for chr_name in sample:
16         chr_data = sample[chr_name]
17         new_len = int(np.ceil(len(chr_data) /
               float(scale)))
18         scaled_chr = np.zeros(new_len, dtype=np.int32)
19         for i in range(new_len):
20             scaled_chr[i] = np.sum(chr_data[int(i *
                   scale) : int(i * scale + scale)])
21             return_sample[chr_name] = scaled_chr
22     return return_sample
```

Listing 15. Buggy function of **scale_sample** in WisecondorX

However, during our evaluation, we observed inconsistent behaviors between original and given versions by BioTester when applying this function to floating-point genomic signals. Several representative input-output examples are summarized as follows:

- [0.1, 0.2, 0.3, 0.4]: when rescaled from bin size 1 to 2, the expected output is [0.3, 0.7], whereas the original implementation returns [0, 0].
- [0.05, 0.15, 0.25, 0.35]: the correct aggregated result [0.2, 0.6] is again reduced to [0, 0].
- [1, 2, 3, 4, 5, 6]: both implementations produce numerically identical outputs [3, 7, 11].
- [1.5, 2.5, 3.5, 4.5]: both implementations produce numerically identical outputs ([4, 8] versus [4.0, 8.0]).

These contrasting behaviors indicate that the correctness of the function is highly input-dependent and motivated a detailed inspection of its implementation. First, the aggregated signal array is originally initialized using an integer data type in line 18, which silently truncates floating-point summation results. This leads to complete signal loss when aggregated values are smaller than one, a common scenario for normalized coverage or log-ratio genomic signals. Second, aggregation boundaries are computed using floating-point arithmetic followed by implicit integer casting in line 14, making the aggregation process sensitive to bin alignment and boundary conditions. Most critically, the output container is updated inside the inner loop that iterates over aggregation windows, as shown in line 21. This state misalignment causes intermediate, partially constructed results to be repeatedly written, making the final output implicitly dependent on loop execution properties rather than on the intended aggregation semantics. Consequently, such erroneous loop coverage represents a hidden yet critical defect. Because the output structure is repeatedly overwritten

during the aggregation loop, the implementation violates the logical separation between per-bin computation and per-chromosome state updates. Notably, this defect remains latent under restricted input conditions, such as integer-valued signals especially larger than 1 with perfectly aligned bin sizes, where truncation effects vanish and the loop completes without interruption. The fixed version given by BioTester is shown as follows.

```
def scale_sample(sample, from_size, to_size):
    if not to_size or from_size == to_size:
        return sample

    if to_size == 0 or from_size == 0 or to_size <
         from_size or to_size % from_size != 0:
        raise ValueError(
            f"Impossible binsize scaling requested:
                {from_size} to {to_size}"
        )

    return_sample = {}
    scale = to_size // from_size

    for chr_name, chr_data in sample.items():
        new_len = int(np.ceil(len(chr_data) / scale))
        scaled_chr = np.zeros(new_len,
             dtype=chr_data.dtype)

        for i in range(new_len):
            start = i * scale
            end = min((i + 1) * scale, len(chr_data))
            scaled_chr[i] = np.sum(chr_data[start:end])

        return_sample[chr_name] = scaled_chr

    return return_sample
```

Listing 16. Fixed implementation given by BioTester

In modlAMP, the **_one_arc** function computes ARC (amphipathic residue cluster) descriptors for an input amino acid sequence given a selected physicochemical scale, which also handles boundaries incorrectly. The code is as follows:

```
def _one_arc(seq, modality, scale):
    desc_mat = []
    for aa in seq:
        desc_mat.append(scale[aa])
    desc_mat = np.asarray(desc_mat)
    desc_dim = desc_mat.shape[1]

    allwindows_arc = []

    if len(seq) > 18:
        window = 18
        num_windows = len(seq) - window
    else:
        window = len(seq)
        num_windows = 1

    for j in range(num_windows):
        ......
        ......
    return final_arc
```

Listing 17. Buggy function of **_one_arc** in modlAMP

It first maps each residue to its scale-specific descriptor vector and then extracts these descriptors across sliding windows along the sequence. For each window, the function reorganizes residue positions according to the helical projection order and evaluates pharmacophoric continuity to derive the maximum arc length for each feature dimension. These per-window values are finally aggregated using either the maximum or mean operator according to the user-specified modality. During the reproduction of this function, we identified an off-by-one error in the original implementation affecting the computation of sliding windows. When the input sequence exceeded 18 residues, the number of windows was calculated as `len(seq) - window`, causing the final valid window to be omitted. The correct formulation should be `len(seq) - window + 1`, ensuring that all admissible windows are included in the descriptor computation. To quantify the effect of the off-by-one sliding window error, we simulated a set of sequences longer than 18 residues along with corresponding scale parameters and the modality parameter. The outputs from both versions were then compared using statistical tests. The simulation of input data follows the following procedure: for each test case, a descriptor type is randomly chosen (either single- or multi-dimensional), a set of amino acids is selected accordingly, and each amino acid is assigned a random descriptor value. A sequence of random length more than 18 is then generated from the chosen amino acids, forming the input for the function. This process is repeated to produce a large number of test cases, enabling a robust assessment of the output differences.

Table S5. Wilcoxon signed-rank test results for different maximum sequence lengths, numbers of test cases, and modalities.

max_len	Number of Test Cases	Modality	p-value
40	100	mean	$2.807 \times 10^{-2} < 0.05$
		max	$5.847 \times 10^{-3} < 0.05$
	500	mean	$3.163 \times 10^{-3} < 0.05$
		max	$7.899 \times 10^{-4} < 0.05$
	1000	mean	$1.195 \times 10^{-5} < 0.05$
		max	$6.735 \times 10^{-6} < 0.05$
50	100	mean	$2.102 \times 10^{-2} < 0.05$
		max	$4.562 \times 10^{-3} < 0.05$
	500	mean	$2.718 \times 10^{-3} < 0.05$
		max	$6.215 \times 10^{-4} < 0.05$
	1000	mean	$9.856 \times 10^{-6} < 0.05$
		max	$5.432 \times 10^{-6} < 0.05$
60	100	mean	$1.895 \times 10^{-2} < 0.05$
		max	$3.987 \times 10^{-3} < 0.05$
	500	mean	$2.451 \times 10^{-3} < 0.05$
		max	$5.731 \times 10^{-4} < 0.05$
	1000	mean	$8.947 \times 10^{-6} < 0.05$
		max	$4.987 \times 10^{-6} < 0.05$

For each simulated input, the outputs of the correct and erroneous versions of the function were computed. To quantify the difference between the two output distributions, we performed a paired non-parametric Wilcoxon signed-rank test, which is suitable for assessing differences between paired samples without assuming a normal distribution. We systematically varied the number of test cases (num_cases=100, 500, 1000) and the maximum sequence length (max_len=40, 50, 60) to evaluate the robustness of the effect. The resulting p-values are summarized in Table S5. Across all tested conditions, the p-values were consistently below the significance threshold of 0.05, indicating that the outputs of the correct and incorrect implementations differ significantly. Specifically, increasing

the number of test cases generally resulted in smaller p-values, reflecting greater statistical power to detect differences. Similarly, larger maximum sequence lengths produced slightly smaller p-values, consistent with the increased opportunity for the off-by-one error to affect the sliding window calculations. Overall, these results confirm that the off-by-one error in the original implementation leads to statistically significant deviations in the computed results.

```
1 def covertLogFoldToNonLog(log_val):
2     try:
3         if float(log_val)<0: nonlog_val =
              (-1/math.pow(2,(float(log_val))))
4         else: nonlog_val = (math.pow(2,float(log_val)))
5     except Exception: nonlog_val = log_val
6     return str(nonlog_val)
```

Listing 18. Buggy function of **covertLogFoldToNonLog** in AltAnalyze

In AltAnalyze, the function **covertLogFoldToNonLog** is intended to convert log-transformed fold-change values back to their non-logarithmic representation. In differential expression analysis, a $\log_2$ fold change value x is typically converted using the relationship 2^x, such that positive and negative values correspond to reciprocal fold changes. However, for negative inputs, the original implementation computes `-1 / 2`^{x} in line 3 instead of preserving the reciprocal relationship implied by the logarithmic transformation. For example, the function maps `log_2FC = -1` to `-2` rather than `-0.5`, and maps `log_2FC = -3.5` to `-11.31` rather than `-0.088`. Consequently, the magnitude of the converted value increases rather than decreases as the log-fold change becomes more negative. The BioTester-generated variant restores this relationship by replacing the incorrect division operator with multiplication, where the formula in line 3 is fixed as:

$$nonlog\_val = (-1 * math.pow(2, (float(log\_val))))$$

Under the revised implementation, the sign continues to indicate the direction of regulation, while the magnitude correctly reflects the corresponding non-logarithmic fold change. This defect represents a typical state misalignment error, where the inconsistency originates from a single misplaced arithmetic operator, causing the internal numerical state to become misaligned with the intended biological semantics.

Impact of defects on analytical results: case study in PEcnv

To further investigate the practical impact of the detected issues on downstream performance, we select PEcnv as a case study. We first corrected the identified implementation-level issues and then conducted simulation experiments following the original experimental pipeline described in the corresponding publication.

PEcnv is a copy number variant detection tool. As PEcnv did, GSDcreator [74] is applied to simulate a set of sequencing data with a coverage depth of 60 and a tumor purity of 0.67. The simulated CNVs had absolute copy numbers ranging from 0 to 6, excluding 2 (normal copy number), and sizes varying between 10 kb and 100 kb. In total, 96 CNV-positive samples were generated, each containing one CNV segment embedded within a 10 Mb long genomic sequence.

The performance comparison between the original PEcnv software and the corrected version is summarized in Table S6. Compared to the original PEcnv, the corrected version demonstrates a notable improvement in sensitivity, increasing from 0.847 to 0.958. This suggests that the correction enables the detection of more true CNV regions, potentially due to enhanced boundary recognition or improved identification of small-scale CNVs. However, the precision dropped from 0.865 to 0.821. The increase in false positives (from 15 to 20) indicates that while more true positives are captured, the corrected method also introduces more incorrect predictions. Despite the precision drop, the F1-score increases from 0.856 to 0.885, indicating a better overall balance between precision and recall. This suggests that the corrected method achieves superior overall detection performance, especially in contexts where higher sensitivity is preferred and a moderate increase in false positives is acceptable.

Table S6. Performance comparison of CNV detection between original and corrected PEcnv implementations.

Methods	True CNV	TP	FP	Sensitivity	Precision	F1-score
PEcnv	96	83	15	0.847	0.865	0.856
PEcnv-corrected	96	92	20	0.958	0.821	0.885

Moreover, the predicted CNV regions against the ground truth are analyzed deeply. Visualizing a subset of 15 samples for example in Figure S6, the true CNV region and the predicted regions from both the original and corrected PEcnv are plotted as horizontal line segments along the genomic coordinate axis. True CNV regions are represented as thick black lines, predictions from PEcnv are shown as red lines, while predictions from PEcnv_corrected are drawn in blue. The results demonstrate that in most cases, the corrected version of PEcnv achieves substantially better coverage of the actual CNV regions compared to the original PEcnv. These findings indicate that detection performance is not solely determined by the theoretical advantages of the underlying algorithms, but is also strongly influenced by the accuracy of their software implementations. High-quality implementations contribute positively to more precise and reliable detection outcomes, which further highlights the need of unit testing for software verification in bioinformatics tools.

Additional notable findings

We also observed a class of anomalies that lie at the boundary between enhanced and incorrect behavior, which we term inconsistent implementations. We identify two primary sources for such inconsistencies. First, guided by retrieved domain-specific knowledge, BioTester may generate implementations that conform to established domain principles but deviate from the behavior of the original code. Second, for the same functional objective, BioTester may synthesize alternative implementations that are both reasonable and well-justified, yet produce outputs that differ from those of the original implementation. When such discrepancies arise, it is often nontrivial to determine whether the observed behavior reflects an unintentional implementation flaw or a deliberate design choice made by the original developers to accommodate specific assumptions or practical constraints. For instance of domain knowledge violation, the `recalculate_gq` function in GATK-SV is designed to transform scaled likelihood values (SL) into Phred-scaled genotype quality (GQ) scores. Conceptually, the function first clamps the input SL within user-defined lower and upper bounds, applies a sign adjustment based on genotype state, and then performs a logarithmic transformation

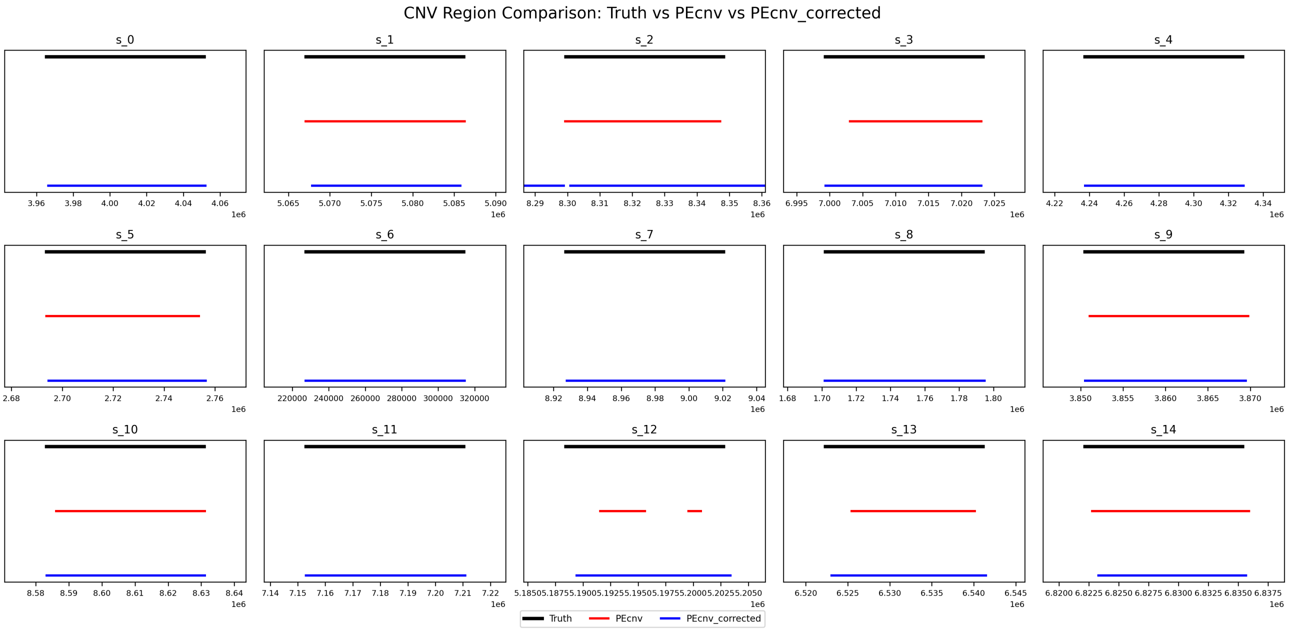

Fig. S6. Detected CNV region comparison before and after correction.

to produce a GQ value. This process aims to convert likelihood-based information into interpretable confidence scores for variant calls. However, through literature and specification retrieval, BioTester noted that the VCF standard defines the valid GQ range as 0-99. Consequently, in the generated alternative implementation, the computed GQ values are explicitly constrained within this range. This additional constraint ensures compliance with the standard but can produce outputs that differ from the original implementation, especially in cases where the calculated values would have exceeded the VCF limits. Therefore, the observed discrepancies between the original and generated implementations arise not from semantic errors in the computation, but from the enforcement of domain-specific output constraints. For instance of alternative implementation, the `gc_fraction` function in Bakta calculates the proportion of guanine (G) and cytosine (C) bases within a given nucleotide sequence. Specifically, it counts the occurrences of the characters `'G'`, `'C'`, and `'S'` in the input string and divides the sum by the sequence length, returning zero for empty sequences. Conceptually, this metric provides a simple measure of the GC content of a genomic region, which is widely used in genome annotation and comparative analysis. Through BioTester analysis, we observed that the original implementation implicitly assumes that the input sequence is in uppercase. As a result, sequences containing lowercase or mixed-case nucleotides systematically produce underestimated GC fractions. However, given the magnitude of this discrepancy, it remains unclear whether the original implementation reflects a deliberate design choice by the developers or represents an unintended oversight.

Moreover, we observed that several inconsistent implementations are concentrated in statistical routines. Typical examples include differences in variance estimation, where one implementation computes the population variance while an alternative adopts the sample variance, as well as notable discrepancies in the implementation of statistical hypothesis tests. For example, the `t_test_1_sample` function in CNVpytor is designed to perform a one-sample t-test, which evaluates whether the mean of a sample differs significantly from a specified population mean. It takes as input the sample mean, the population mean, the sample standard deviation, and the sample size, and returns a p-value quantifying the statistical significance of the deviation. However, the original implementation computes the p-value using `1.0 - stats.t.cdf(abs(t))`, which corresponds to a single-tailed probability. BioTester provided an alternative implementation that computes a two-sided p-value using the formula `2 * (1 - CDF(|t|))`, which aligns with the standard methodology for a one-sample t-test. While both implementations are mathematically plausible, this discrepancy can lead to differing outputs without necessarily indicating a clear error in either version.
