# Supplementary material for: BioTester: an AI-driven automated testing framework for identifying potential quality risks in bioinformatics software
Source: Brief Bioinform. 2026 Sep 16;27(5):bbag452. doi: 10.1093/bib/bbag452 (PMC13580090; doi:10.1093/bib/bbag452)
Supplement: Supplementary file 1 [file supplementary_material_bbag452.zip › supplementary_all_results_bbag452.docx]

| **software** | **test_program** | **BioTester_generation** | **assertion with expected results** | **actual results** | **analysis** | **Risk level** | **reason** |
| --- | --- | --- | --- | --- | --- | --- | --- |
| cnvkit | def q_n(a):  """Rousseeuw & Croux's (1993) Q_n, an alternative to MAD.   ``Qn := Cn first quartile of (\|x_i - x_j\|: i < j)``   where Cn is a constant depending on n.   Finite-sample correction factors must be used to calibrate the  scale of Qn for small-to-medium-sized samples.   n E[Qn]  -- -----  10 1.392  20 1.193  40 1.093  60 1.064  80 1.048  100 1.038  200 1.019   """  # First quartile of: (\|x_i - x_j\|: i < j)  vals = []  for i, x_i in enumerate(a):  for x_j in a[i + 1 :]:  vals.append(abs(x_i - x_j))  quartile = np.percentile(vals, 25)   # Cn: a scaling factor determined by sample size  n = len(a)  if n <= 10:  # ENH: warn when extrapolating beyond the data  # ENH: simulate for values up to 10  # (unless the equation below is reliable)  scale = 1.392  elif 10 < n < 400:  # I fitted the simulated values (above) to a power function in Excel:  # f(x) = 1.0 + 3.9559 * x ^ -1.0086  # This should be OK for interpolation. (Does it apply generally?)  scale = 1.0 + (4 / n)  else:  scale = 1.0   return quartile / scale | def q_n(data):  """Rousseeuw & Croux's (1993) Q_n, an alternative to MAD.   ``Qn := Cn first quartile of (\|x_i - x_j\|: i < j)``   where Cn is a constant depending on n.   Finite-sample correction factors must be used to calibrate the  scale of Qn for small-to-medium-sized samples.   n E[Qn]  -- -----  10 1.392  20 1.193  40 1.093  60 1.064  80 1.048  100 1.038  200 1.019   """  Q_n = 0  n = len(data)   if n <= 1:  return Q_n   absolute_differences = []   for i in range(n):  for j in range(i + 1, n):  absolute_differences.append(abs(data[i] - data[j]))   absolute_differences.sort()   first_quartile_index = int(0.25 * len(absolute_differences))   if n <= 10:  C_n = 1.0  elif n > 10 and n < 400:  C_n = 0.5 * (n ** (1/3))  else:  C_n = 1.0   Q_n = absolute_differences[first_quartile_index] / C_n   return Q_n | assert q_n([1, 2, 3, 4, 5]) == 1.0 assert q_n([1]) == 0 assert q_n([1, 2]) == 1.0 #0.7183908045977012 assert q_n([1, 2, 3, 4, 5, 6, 7, 8, 9, 10]) == 2.0 assert q_n([1, 2, 3, 4, 5, 6, 7, 8, 9, 10, 11]) == 1.7985772520904368 | q_n([1, 2, 3, 4, 5]) == 0.7183908045977012 q_n([1]) == 0 q_n([1, 2]) == 0.7183908045977012 q_n([1, 2, 3, 4, 5, 6, 7, 8, 9, 10]) == 1.4367816091954024 q_n([1, 2, 3, 4, 5, 6, 7, 8, 9, 10, 11]) == 1.4666666666666666 | Both implementations serve the same purpose: computing the Rousseeuw & Croux Qₙ scale estimator, which estimates data dispersion based on the first quartile (25% quantile) of all pairwise differences in the sample, adjusted by a correction factor that depends on the sample size. However, the way the correction factor Cn is implemented differs. | medium | alternative implementation |
| cnvkit | def FDRThres(x, q, stdev):  """False discovery rate (FDR) threshold."""  M = len(x)  if M < 2:  return 0   m = np.arange(1, M + 1) / M  x_sorted = np.sort(np.abs(x))[::-1]  p = 2 * (1 - stats.norm.cdf(x_sorted, stdev)) # like R "pnorm"  # Get the largest index for which p <= m*q  indices = np.nonzero(p <= m * q)[0]  if len(indices):  T = x_sorted[indices[-1]]  else:  logging.debug(  "No passing p-values: min p=%.4g, min m=%.4g, q=%s", p[0], m[0], q  )  T = x_sorted[0] + 1e-16 # ~= 2^-52, like MATLAB "eps"  return T | def FDRThres(x, q, stdev):  """False discovery rate (FDR) threshold."""  M = len(x)  if M < 2:  return 0   m = np.arange(1, M + 1) / M  x_sorted = np.sort(np.abs(x))[::-1]  p = 2 * (1 - stats.norm.cdf(x_sorted, loc=0, scale=stdev)) # like R "pnorm"  # Get the largest index for which p <= m*q  indices = np.nonzero(p <= m * q)[0]  if len(indices):  T = x_sorted[indices[-1]]  else:  logging.debug(  "No passing p-values: min p=%.4g, min m=%.4g, q=%s", p[0], m[0], q  )  T = x_sorted[0] + 1e-16 # ~= 2^-52, like MATLAB "eps"  return T | assert calculateFDRThreshold([0.1, 0.2, 0.3], 0.05,0.05) == 0.1 assert calculateFDRThreshold([-0.1, -0.2, -0.3], 0.05,0.05) == 0.1  assert calculateFDRThreshold([0.1, 0.2, 0.3], 0.05,0) == 0.3000000000000001 assert calculateFDRThreshold([0.1, 0.2, 0.3], 0.05,1) == 0.3000000000000001 assert calculateFDRThreshold([0.1, 0.2, 0.3, 0.4, 0.5], 0.05,0.1) == 0.3  assert calculateFDRThreshold([0.5, 0.4, 0.3, 0.2, 0.1], 0.05,0.1) == 0.3  assert calculateFDRThreshold([1.0, 2.0, 3.0], 0.05,0.5) == 1.0 assert calculateFDRThreshold([1.0, 2.0, 3.0], 0.05,0.9) == 2.0 | calculateFDRThreshold([0.1, 0.2, 0.3], 0.05,0.05) == 0.3000000000000001 calculateFDRThreshold([-0.1, -0.2, -0.3], 0.05,0.05) == 0.3000000000000001 calculateFDRThreshold([0.1, 0.2, 0.3], 0.05,0) == 0.3000000000000001 calculateFDRThreshold([0.1, 0.2, 0.3], 0.05,1) == 0.3000000000000001 calculateFDRThreshold([0.1, 0.2, 0.3, 0.4, 0.5], 0.05,0.1) == 0.5000000000000001 calculateFDRThreshold([0.5, 0.4, 0.3, 0.2, 0.1], 0.05,0.1) == 0.5000000000000001 calculateFDRThreshold([1.0, 2.0, 3.0], 0.05,0.5) == 3.0 calculateFDRThreshold([1.0, 2.0, 3.0], 0.05,0.9) == 3.0 | When stdev ≠ 1 and the parameter is mistakenly interpreted as loc (mean) rather than scale (standard deviation) due to incorrect argument positioning, the computed p-value will be incorrect, leading to a deviation in the resulting threshold. | high | Parameter passing errors |
| cnvkit | def FindLocalPeaks(  signal, # const double * signal,  # peakLoc, #int * peakLoc ):  """Find local maxima on positive values, local minima on negative values.   First and last index are never considered extramum.   Parameters  ----------  signal : const array of floats   Returns  -------  peakLoc : array of ints  Locations of extrema in `signal`   Source: HaarSeg.c  """  # use numpy.diff to simplify? argmax, argmin?  maxSuspect = minSuspect = None  peakLoc = []  for k in range(1, len(signal) - 1):  sig_prev, sig_curr, sig_next = signal[k - 1 : k + 2]  if sig_curr > 0:  # Look for local maxima  if (sig_curr > sig_prev) and (sig_curr > sig_next):  peakLoc.append(k)  elif (sig_curr > sig_prev) and (sig_curr == sig_next):  maxSuspect = k  elif (sig_curr == sig_prev) and (sig_curr > sig_next):  # Take the first in a series of equal values  if maxSuspect is not None:  peakLoc.append(maxSuspect)  maxSuspect = None  elif (sig_curr == sig_prev) and (sig_curr < sig_next):  maxSuspect = None   elif sig_curr < 0:  # Look for local maxima  if (sig_curr < sig_prev) and (sig_curr < sig_next):  peakLoc.append(k)  elif (sig_curr < sig_prev) and (sig_curr == sig_next):  minSuspect = k  elif (sig_curr == sig_prev) and (sig_curr < sig_next):  if minSuspect is not None:  peakLoc.append(minSuspect)  minSuspect = None  elif (sig_curr == sig_prev) and (sig_curr > sig_next):  minSuspect = None   return np.array(peakLoc, dtype=np.int_) | def FindLocalPeaks(signal):  """Find local maxima on positive values, local minima on negative values.   First and last index are never considered extramum.   Parameters  ----------  signal : const array of floats   Returns  -------  peakLoc : array of ints  Locations of extrema in `signal`  """  peak_locations = []  suspect_maxima = []  suspect_minima = []   if len(signal) < 3:  return []   for i in range(1, len(signal) - 1):  if signal[i] > 0:  if signal[i] > signal[i - 1]:  suspect_maxima.append(i)  elif signal[i] < signal[i - 1]:  if len(suspect_maxima) > 0:  peak_locations.append(suspect_maxima[-1])  suspect_maxima.clear()  elif signal[i] < 0:  if signal[i] < signal[i - 1]:  suspect_minima.append(i)  elif signal[i] > signal[i - 1]:  if len(suspect_minima) > 0:  peak_locations.append(suspect_minima[-1])  suspect_minima.clear()   if len(suspect_maxima) > 0:  peak_locations.append(suspect_maxima[-1])  if len(suspect_minima) > 0:  peak_locations.append(suspect_minima[-1])   return peak_locations | assert FindLocalPeaks([1, 2, 3, 4, 5]).tolist() == [3] assert FindLocalPeaks([-1, -2, -3, -4, -5]).tolist() == [3] assert FindLocalPeaks([1, -1, 1, -1, 1]).tolist() == [2, 3] assert FindLocalPeaks([1, 0, -1, 0, 1]).tolist() == [2] assert FindLocalPeaks([0, 0, 0]).tolist() == [] assert FindLocalPeaks([1, 2, -1, -2, 1]).tolist() == [1, 3] assert FindLocalPeaks([-1, -2, 1, 2, -1]).tolist() == [3, 1] assert FindLocalPeaks([1, 2, 1, 0, -1, -2]).tolist() == [1, 4] assert FindLocalPeaks([0, 1, 0, -1, 0]).tolist() == [1, 3] assert FindLocalPeaks([1, 2, 1, 2, 1]).tolist() == [1, 3] | FindLocalPeaks([1, 2, 3, 4, 5]).tolist() == [] FindLocalPeaks([-1, -2, -3, -4, -5]).tolist() == [] FindLocalPeaks([1, -1, 1, -1, 1]).tolist() == [1,2,3] FindLocalPeaks([1, 0, -1, 0, 1]).tolist() == [2] FindLocalPeaks([0, 0, 0]).tolist() == [] FindLocalPeaks([1, 2, -1, -2, 1]).tolist() == [1, 3] FindLocalPeaks([-1, -2, 1, 2, -1]).tolist() == [1,3] FindLocalPeaks([1, 2, 1, 0, -1, -2]).tolist() == [1] FindLocalPeaks([0, 1, 0, -1, 0]).tolist() == [1, 3] FindLocalPeaks([1, 2, 1, 2, 1]).tolist() == [1, 3] | The logic for ignoring the sequence boundaries differs between the two versions. Additionally, the output order in the BioTester version is based on the highest and lowest peaks, rather than the original index order. | medium | alternative implementation |
| cnvkit | def PulseConv(  signal, # const double * signal,  pulseSize, # int pulseSize, ):  """Convolve a pulse function with a signal, applying circular padding to the  signal.   Used for non-stationary variance compensation.   Parameters  ----------  signal: const array of floats  pulseSize: int   Returns  -------  array of floats   Source: HaarSeg.c  """  signalSize = len(signal)  if pulseSize > signalSize:  # ENH: handle this endcase  raise ValueError(f"pulseSize ({pulseSize}) > signalSize ({signalSize})")  pulseHeight = 1.0 / pulseSize   # Circular padding init  result = np.zeros(signalSize, dtype=np.float64)  for k in range((pulseSize + 1) // 2):  result[0] += signal[k]  for k in range(pulseSize // 2):  result[0] += signal[k]  result[0] *= pulseHeight   n = 1  for k in range(pulseSize // 2, signalSize + (pulseSize // 2) - 1):  tail = k - pulseSize  if tail < 0:  tail = -tail - 1  head = k  if head >= signalSize:  head = signalSize - 1 - (head - signalSize)  result[n] = result[n - 1] + ((signal[head] - signal[tail]) * pulseHeight)  n += 1   return result | def PulseConv(signal, pulseSize):  """Convolve a pulse function with a signal, applying circular padding.   Used for non-stationary variance compensation.   Parameters  ----------  signal: const array of floats  pulseSize: int   Returns  -------  array of floats   Source: HaarSeg.c  """  signalSize = len(signal)  if pulseSize > signalSize:  raise ValueError(f"pulseSize ({pulseSize}) > signalSize ({signalSize})")  pulseHeight = 1.0 / pulseSize   result = np.zeros(signalSize, dtype=np.float64)   half = pulseSize // 2  sum_val = 0.0  for k in range(-half, pulseSize - half):  idx = (k + signalSize) % signalSize  sum_val += signal[idx]  result[0] = sum_val * pulseHeight   for n in range(1, signalSize):  tail_idx = (n - 1 - half + signalSize) % signalSize  head_idx = (n + pulseSize - 1 - half) % signalSize  result[n] = result[n - 1] + pulseHeight * (signal[head_idx] - signal[tail_idx])   return result | assert test_PulseConv([1.0, 2.0, 3.0], 1) == '[1.0, 2.0, 3.0]' assert test_PulseConv([1.0, 2.0, 3.0], 2) == '[2.0, 1.5, 2.5]' assert test_PulseConv([1.0, 2.0, 3.0], 3) == '[2.0, 2.0, 2.0]'  assert test_PulseConv([0.0, 0.0, 0.0], 1) == '[0.0, 0.0, 0.0]' assert test_PulseConv([1.0, 2.0, 3.0, 4.0], 4) == '[2.5, 2.5, 2.5, 2.5]' assert test_PulseConv([1.0], 1) == '[1.0]' | test_PulseConv([1.0, 2.0, 3.0], 1) == [1.0, 1.0, 2.0] test_PulseConv([1.0, 2.0, 3.0], 2) == [1.0, 1.5, 2.5] test_PulseConv([1.0, 2.0, 3.0], 3) == [1.3333333333333333, 1.3333333333333333, 2.0] test_PulseConv([0.0, 0.0, 0.0], 1) == [0.0, 0.0, 0.0] test_PulseConv([1.0, 2.0, 3.0, 4.0], 4) == [1.5, 1.75, 2.5, 3.25] test_PulseConv([1.0], 1) == [1.0] | The two implementations differ primarily in how they handle boundary conditions and initialize the convolution window. The original version uses mirrored boundary reflection when accessing out-of-bound indices and applies an unclear initialization to result[0], which may lead to duplicated contributions. In contrast, the BioTester version strictly applies circular padding using modular indexing, ensuring mathematically correct circular convolution. Additionally, its sliding window implementation is more readable and robust. | medium | alternative implementation |
| cnvkit | def tsl2int(tsl):  """Convert an Ensembl Transcript Support Level (TSL) code to an integer.   The code has the format "tsl([1-5]\|NA)".   See: https://www.ensembl.org/Help/Glossary?id=492  """  if tsl in (np.nan, "", "tslNA"):  return 0  # Remove "tsl" prefix  assert tsl[:3] == "tsl"  value = tsl[3:]  # Remove possible suffix " (assigned to previous version _)"  if len(value) > 2:  value = value[:2].rstrip()  if value == "NA":  return 0  return int(value) | def tsl2int(tslCode):  if tslCode is None or tslCode == "" or tslCode == "tslNA":  return 0    if not tslCode.startswith("tsl"):  return 0    numericPart = tslCode[3:] # Extract substring starting from index 4    if numericPart == "NA":  return 0    return int(numericPart) | assert tsl2int("tsl123") == '123' assert tsl2int("tsl456") == '456' | tsl2int("tsl123") == 12 tsl2int("tsl456") == 45 | The two implementations achieve the same goal—converting Ensembl TSL codes into integers—but differ in robustness and clarity. The original tsl2int function includes additional checks for np.nan, an assert statement on the prefix, and a somewhat unclear substring truncation. In contrast, tsl2int adopts a cleaner and safer approach, using startswith() and defensive checks against None and malformed inputs. It is generally more robust and preferable in production code. | low |  |
| cnvkit | def first_of(elems):  """Return the first element of the input."""  return elems[0] | def first_of(elems):  """Return the first element of the input."""  if not elems:  return None  else:  return elems[0] | assert first_of([]) == None assert first_of([1]) == 1 assert first_of(['a', 'b', 'c']) == 'a' assert first_of([None]) == None assert first_of([0]) == 0 | first_of([]) raises IndexError first_of([1]) == 1 first_of(['a', 'b', 'c']) == 'a' first_of([None]) == None first_of([0]) == 0 | The two versions differ in how they handle empty inputs. The original version assumes the input is non-empty and directly returns the first element, which can lead to an IndexError if the input is empty. The revised version adds an explicit check and returns None when the input is empty, making it more robust and safer for general use. | low |  |
| cnvkit | def normalize(A):  """Normalize matrix columns."""  return A / A.sum(axis=0) | def normalize(A):  """Normalize matrix columns."""  num_rows = len(A)  num_cols = len(A[0])  normalized_A = [[0] * num_cols for _ in range(num_rows)]   for j in range(num_cols):  column_sum = 0  for i in range(num_rows):  column_sum += A[i][j]   if column_sum == 0:  for i in range(num_rows):  normalized_A[i][j] = A[i][j]  else:  for i in range(num_rows):  normalized_A[i][j] = A[i][j] / column_sum   return normalized_A | assert normalize([[1, 2], [3, 4]]) == [[0.25, 0.3333333333333333], [0.75, 0.6666666666666666]] assert normalize([[0, 0], [0, 0]]) == [[0, 0], [0, 0]] assert normalize([[1, 0], [0, 0]]) == [[1.0, 0], [0.0, 0]] assert normalize([[1, 2, 3], [4, 5, 6]]) == [[0.2, 0.2857142857142857, 0.3333333333333333], [0.8, 0.7142857142857143, 0.6666666666666666]] assert normalize([[0, 1], [0, 2], [0, 3]]) == [[0, 0.16666666666666666], [0, 0.3333333333333333], [0, 0.5]] assert normalize([[1, 2], [0, 0]]) == [[1.0, 1.0], [0.0, 0.0]] assert normalize([[5, 0], [0, 0]]) == [[1.0, 0], [0.0, 0]] assert normalize([[1], [2], [3]]) == [[0.16666666666666666], [0.3333333333333333], [0.5]] assert normalize([[0]]) == [[0]] assert normalize([[1, 1], [1, 1]]) == [[0.5, 0.5], [0.5, 0.5]] | normalize([[1, 2], [3, 4]]) == [[0.25, 0.3333333333333333], [0.75, 0.6666666666666666]] normalize([[0, 0], [0, 0]]) == [[nan, nan], [nan, nan]] normalize([[1, 0], [0, 0]]) == [[1.0, nan], [0.0, nan]] normalize([[1, 2, 3], [4, 5, 6]]) == [[0.2, 0.2857142857142857, 0.3333333333333333], [0.8, 0.7142857142857143, 0.6666666666666666]] normalize([[0, 1], [0, 2], [0, 3]]) == [[nan, 0.16666666666666666], [nan, 0.3333333333333333], [nan, 0.5]] normalize([[1, 2], [0, 0]]) == [[1.0, 1.0], [0.0, 0.0]] normalize([[5, 0], [0, 0]]) == [[1.0, nan], [0.0, nan]] normalize([[1], [2], [3]]) == [[nan]] normalize([[1, 1], [1, 1]]) == [[0.5, 0.5], [0.5, 0.5]] | Both functions normalize the columns of a matrix such that each column sums to 1. The first version leverages NumPy for a concise and efficient implementation but does not handle zero-sum columns, which may result in NaN values. The second version, written in pure Python, explicitly checks for zero-sum columns and retains the original values in such cases, making it more robust but less efficient and more verbose. | low |  |
| cnvkit | def _get_alt_count(sample):  """Get the alternative allele count from a sample in a VCF record."""  if sample.get("AD") not in (None, (None,)):  # GATK and other callers: (ref depth, alt depth)  if isinstance(sample["AD"], tuple):  # Ensure we have alternative alleles and thus two AD values  # ref only calls in GATK can be missing these  # 1 49515 . G . 50.80 . AN=2;DP=34;MQ=40.01 GT:AD:DP:MMQ 0/0:34:34:.  if len(sample["AD"]) > 1:  alt_count = sample["AD"][1]  else:  alt_count = 0.0  # VarScan  else:  alt_count = sample["AD"]  elif sample.get("CLCAD2") not in (None, (None,)):  # Qiagen CLC Genomics Server -- similar to GATK's AD  alt_count = sample["CLCAD2"][1]  elif "AO" in sample:  if isinstance(sample["AO"], tuple):  alt_count = _safesum(sample["AO"])  elif sample["AO"]:  alt_count = sample["AO"]  else:  alt_count = 0.0  else:  alt_count = np.nan  return alt_count | def _get_alt_count(sample_data):  if "AD" not in sample_data and "CLCAD2" not in sample_data and "AO" not in sample_data:  return 0.0   alternative_count = 0.0   if "AD" in sample_data:  ad_values = sample_data["AD"].split(",")  if len(ad_values) >= 2:  alternative_count = float(ad_values[1])  else:  return 0.0   if "CLCAD2" in sample_data:  clcad2_value = float(sample_data["CLCAD2"])  if not (clcad2_value != clcad2_value): # Check for NaN  alternative_count = _safesum(alternative_count, clcad2_value)   if "AO" in sample_data:  ao_value = float(sample_data["AO"])  if not (ao_value != ao_value): # Check for NaN  alternative_count = _safesum(alternative_count, ao_value)   return alternative_count  def _safesum(value1, value2):  if value1 != value1: # Check for NaN  return value2  if value2 != value2: # Check for NaN  return value1  return value1 + value2 | assert _get_alt_count({}) == 0.0 assert _get_alt_count({"AD": (0, 1)}) == 1.0 assert _get_alt_count({"AD": (0, 1), "CLCAD2": (2.0,)}) == 1.0 assert _get_alt_count({"AD": (0, 1), "AO": (3.0,)}) == 1.0 assert _get_alt_count({"CLCAD2": (2.0,)}) == 2.0 assert _get_alt_count({"AO": (3.0,)}) == 3.0 assert _get_alt_count({"AD": (0, 1), "CLCAD2": (np.nan,)}) == 1.0 assert _get_alt_count({"AD": (0, 1), "AO": (np.nan,)}) == 1.0 assert _get_alt_count({"CLCAD2": (np.nan,), "AO": (3.0,)}) == 3.0 assert _get_alt_count({"AD": (0, 1), "CLCAD2": (2.0,), "AO": (3.0,)}) == 1.0 | _get_alt_count({}) raises IndexError _get_alt_count({"AD": (0, 1)}) == 1.0 _get_alt_count({"AD": (0, 1), "CLCAD2": (2.0,)}) == 1.0 _get_alt_count({"AD": (0, 1), "AO": (3.0,)}) == 1.0 _get_alt_count({"CLCAD2": (2.0,)}) raises IndexError _get_alt_count({"AO": (3.0,)}) == 3.0 _get_alt_count({"AD": (0, 1), "CLCAD2": (np.nan,)}) == 1.0 _get_alt_count({"AD": (0, 1), "AO": (np.nan,)})raises IndexError _get_alt_count({"CLCAD2": (np.nan,), "AO": (3.0,)}) raises IndexError _get_alt_count({"AD": (0, 1), "CLCAD2": (2.0,), "AO": (3.0,)}) == 1.0 | The two functions aim to extract the alternative allele count from a VCF sample, but differ significantly in input assumptions and logic. _get_alt_count expects parsed values (e.g., tuples or floats), and returns the first available field among AD, CLCAD2, or AO. In contrast, _get_alt_count assumes raw string values and attempts to parse and accumulate counts from multiple fields. While the first is tailored for VCF-parsed objects (e.g., PyVCF), the second is more flexible for raw or loosely formatted data. Their return values and NaN handling also differ, with _get_alt_count returning np.nan on failure, and _get_alt_count defaulting to 0.0. | medium | domain knowledge violation |
| cnvkit | def parse_end_from_info(info):  """Parse END position, if present, from an INFO field."""  idx = info.find("END=")  if idx == -1:  return -1  info = info[idx + 4 :]  idx = info.find(";")  if idx != -1:  info = info[:idx]  return int(info) | def parse_end_from_info(INFO):  startIndex = INFO.find("END=")  if startIndex == -1:  return -1  endIndex = INFO.find(";", startIndex)  if endIndex == -1:  endIndex = len(INFO)  endValue = INFO[startIndex + 4:endIndex]  if endValue.isdigit() or (endValue[0] == '-' and endValue[1:].isdigit()):  return int(endValue)  else:  return -1 | assert parse_end_from_info("END=123;") == 123 assert parse_end_from_info("END=-456;") == -456 assert parse_end_from_info("END=789") == 789 assert parse_end_from_info("START=100; END=321;") == 321 assert parse_end_from_info("END=abc;") == -1 assert parse_end_from_info("No END here;") == -1 assert parse_end_from_info("END=-;") == -1 assert parse_end_from_info("END=0;") == 0 | parse_end_from_info("END=123;") == 123 parse_end_from_info("END=-456;") == -456 parse_end_from_info("END=789") == 789 parse_end_from_info("START=100; END=321;") == 321 parse_end_from_info("END=abc;") raises error parse_end_from_info("No END here;") == -1 parse_end_from_info("END=-;") raises error parse_end_from_info("END=0;") == 0 | Both functions aim to extract the END position from a VCF INFO string. While the first version (parse_end_from_info) is concise, it assumes the extracted value is always a valid integer and may raise a ValueError if the format is malformed. In contrast, parse_end_from_info includes additional validation to ensure the value is a valid integer (including negative numbers), returning -1 if not. This makes the latter more robust and safer in production scenarios. | low |  |
| cnvkit | def weighted_median(a, weights):  """Weighted median of a 1-D numeric array."""  order = a.argsort()  a = a[order]  weights = weights[order]  midpoint = 0.5 * weights.sum()  if (weights > midpoint).any():  # Any point with the majority of total weight must be the median  return a[weights.argmax()]  cumulative_weight = weights.cumsum()  midpoint_idx = cumulative_weight.searchsorted(midpoint)  if (  midpoint_idx > 0  and cumulative_weight[midpoint_idx - 1] - midpoint < sys.float_info.epsilon  ):  # Midpoint of 2 array values  return a[midpoint_idx - 1 : midpoint_idx + 1].mean()  return a[midpoint_idx] | def weighted_median(array, weights):  """Weighted median of a 1-D numeric array."""  if len(array) != len(weights):  return "ERROR: Array and weights must have the same length"    combined_array = list(zip(array, weights))  combined_array.sort(key=lambda x: x[0])    cumulative_weight = 0  total_weight = sum(weights)  midpoint = total_weight / 2    for value, weight in combined_array:  cumulative_weight += weight  if cumulative_weight >= midpoint:  return value    return "ERROR: No median found" | assert weighted_median([1, 2, 3], [1, 1, 1]) == '2' assert weighted_median([1, 2, 3], [1, 2, 3]) == '2' assert weighted_median([1, 2, 3, 4], [1, 1, 1, 1]) == '2' assert weighted_median([1, 2, 3, 4], [1, 2, 2, 1]) == '2' assert weighted_median([1, 2, 3], [0, 0, 0]) == '1' assert weighted_median([], []) == "'ERROR: No median found'" assert weighted_median([1, 2], [1, 1]) == '1' assert weighted_median([1, 2, 3], [1, 0, 0]) == '1' assert weighted_median([1, 2, 3], [0, 1, 0]) == '2' assert weighted_median([1, 2, 3], [0, 0, 1]) == '3' assert weighted_median([1, 2, 3], [1, 1]) == "'ERROR: Array and weights must have the same length'" assert weighted_median([1, 2, 3, 4, 5], [1, 1, 1, 1, 1]) == '3' assert weighted_median([1, 2, 3, 4, 5], [1, 2, 3, 4, 5]) == '4'  assert weighted_median([1, 2, 3, 4, 5], [5, 4, 3, 2, 1]) == '2' | weighted_median([1, 2, 3], [1, 1, 1]) == 1.5 weighted_median([1, 2, 3], [1, 2, 3]) == 1.5 weighted_median([1, 2, 3, 4], [1, 1, 1, 1]) == 1.5 weighted_median([1, 2, 3, 4], [1, 2, 2, 1]) == 1.5 weighted_median([1, 2, 3], [0, 0, 0]) == '1' weighted_median([], []) == "'ERROR: No median found'" weighted_median([1, 2], [1, 1]) == '1' weighted_median([1, 2, 3], [1, 0, 0]) == '1' weighted_median([1, 2, 3], [0, 1, 0]) == '2' weighted_median([1, 2, 3], [0, 0, 1]) == '3' weighted_median([1, 2, 3], [1, 1]) == "'ERROR: Array and weights must have the same length'" weighted_median([1, 2, 3, 4, 5], [1, 1, 1, 1, 1]) == 2.5 weighted_median([1, 2, 3, 4, 5], [1, 2, 3, 4, 5]) == 3.5 weighted_median([1, 2, 3, 4, 5], [5, 4, 3, 2, 1]) == 1.5 | Both functions compute the weighted median of a one-dimensional array with associated weights, but differ in approach and robustness. The first function, using NumPy, handles edge cases like weights exceeding half the total, and precisely computes the median even when the cumulative weight lies exactly between two elements by averaging them. The second function, implemented in pure Python, uses a simpler cumulative sum loop without such checks and returns error strings on invalid input. | medium | alternative implementation |
| cnvkit | def gapper_scale(a):  """Scale estimator based on gaps between order statistics.   See:   - Wainer & Thissen (1976)  - Beers, Flynn, and Gebhardt (1990)  """  gaps = np.diff(np.sort(a))  n = len(a)  idx = np.arange(1, n)  weights = idx * (n - idx)  return (gaps * weights).sum() * np.sqrt(np.pi) / (n * (n - 1)) | def gapper_scale(data):  """Estimate scale based on gaps between sorted data."""  data.sort()  n = len(data)  weightedSum = 0  totalWeight = 0   for i in range(1, n):  gap = data[i] - data[i - 1]  weight = i  weightedSum += weight * gap  totalWeight += weight   if totalWeight > 0:  scaleEstimate = weightedSum / totalWeight  else:  scaleEstimate = 0   return scaleEstimate | assert gapper_scale([1, 2, 3, 4, 5]) == '1.0' assert gapper_scale([5, 4, 3, 2, 1]) == '1.0' assert gapper_scale([1, 1, 1, 1]) == '0.0' assert gapper_scale([1, 2, 2, 3, 4]) == '0.8' assert gapper_scale([10]) == '0' assert gapper_scale([]) == '0' | gapper_scale([1, 2, 3, 4, 5]) == 1.7724538509055159 gapper_scale([5, 4, 3, 2, 1]) == 1.7724538509055159 gapper_scale([1, 1, 1, 1]) == 0.0 gapper_scale([1, 2, 2, 3, 4]) == 1.2407176956338613 gapper_scale([10]) == 0 gapper_scale([]) == 0 | Both functions estimate scale based on the gaps between sorted data points. The first function, gapper_scale, implements a well-known robust scale estimator that weights each gap by the product i×(n−i) and applies a normalization factor involving π^(0.5). This method is supported by statistical literature (Wainer & Thissen, 1976; Beers et al., 1990). The second function, gapper_scale, performs a simpler weighted average of gaps with weights proportional only to the index i and lacks the symmetric weighting and normalization factor, resulting in a less theoretically grounded estimate. | medium | domain knowledge violation |
| PEcnv | def merge_segments(segs):  newSegs = []  tm = []  for li in segs:  if len(tm) == 0:  tm = li  newSegs.append(tm)  else:  if li[0] <= tm[1]:  tm = [tm[0], li[1]]  del (newSegs[-1])  newSegs.append(tm)  else:  newSegs.append(li)  tm = li  return newSegs | def merge_segments(inputSegments):  newSegs = []  tm = []  for segment in inputSegments:  if not tm:  tm = segment  newSegs.append(tm)  else:  if segment[0] <= tm[1]:  tm[1] = max(tm[1], segment[1])  else:  tm = segment  newSegs.append(tm)  return newSegs | assert merge_segments([[1, 5], [2, 3], [4, 6]]) == [[1, 6]] | merge_segments([[1, 5], [2, 3], [4, 6]]) == [[1, 3], [4, 6]] | Both functions merge overlapping intervals from a list of segments. The first version replaces the last merged segment by deleting and re-adding a new combined interval using the end coordinate of the current segment, which may inadvertently shorten intervals. The second version modifies the end coordinate of the last merged segment in place using the maximum of both intervals' end points, ensuring the merged intervals are always correctly extended. The second implementation is therefore more efficient, concise, and robust. | high | State Misalignment Error |
| PEcnv | def _pad_array(x, wing):  return np.concatenate((x[wing - 1::-1],  x,  x[:-wing - 1:-1])) | def _pad_array(x, wing):  left_padding = x[:wing][::-1]  right_padding = x[-wing:][::-1]  return np.concatenate((left_padding, x, right_padding)) | assert _pad_array([1, 2, 3, 4], 0).tolist() == [1, 2, 3, 4, 4, 3, 2, 1] assert _pad_array([1, 2, 3, 4, 5], 0).tolist() == [1, 2, 3, 4, 5, 5, 4, 3, 2, 1] | _pad_array([1, 2, 3, 4], 0).tolist() == [4.0, 3.0, 2.0, 1.0, 1.0, 2.0, 3.0, 4.0] _pad_array([1, 2, 3, 4, 5], 0).tolist() == [5.0, 4.0, 3.0, 2.0, 1.0, 1.0, 2.0, 3.0, 4.0, 5.0] | Both functions perform symmetric padding on the input array by concatenating a reversed slice from the left and right edges of the array. The first function uses more intricate slicing notation (x[wing-1::-1] and x[:-wing-1:-1]), which may be harder to read but produces the same effect as the second function. The second function’s slicing (x[:wing][::-1] and x[-wing:][::-1]) is more straightforward and easier to understand, making it preferable for maintainability. | medium | alternative implementation |
| PEcnv | def merge_segs(segs):  newSegs = []  tm = []  for li in segs:  if len(tm) == 0:  tm = li  newSegs.append(tm)  else:  if li[0] <= tm[1]:  tm = [tm[0], li[1]]  del (newSegs[-1])  newSegs.append(tm)  else:  newSegs.append(li)  tm = li  return newSegs | def merge_segs(segs):  if not segs:  return []   segs.sort() # Ensure segments are sorted by start  merged = [segs[0]]   for current in segs[1:]:  last = merged[-1]  if current[0] <= last[1]: # overlap  last[1] = max(last[1], current[1])  else:  merged.append(current)   return merged | assert merge_segs([[1, 5], [2, 3], [4, 6]])== [[1,6]] | merge_segs([[1, 5], [2, 3], [4, 6]])== [[1, 3], [4, 6]] | Both functions merge overlapping intervals from a list of segments. The first version replaces the last merged segment by deleting and re-adding a new combined interval using the end coordinate of the current segment, which may inadvertently shorten intervals. The second version modifies the end coordinate of the last merged segment in place using the maximum of both intervals' end points, ensuring the merged intervals are always correctly extended. The second implementation is therefore more efficient, concise, and robust. | high | Boundary handling errors |
| PEcnv | def FDRThres(x, q, stdev):  """False discovery rate (FDR) threshold."""  M = len(x)  if M < 2:  return 0   m = np.arange(1, M + 1) / M  x_sorted = np.sort(np.abs(x))[::-1]  p = 2 * (1 - stats.norm.cdf(x_sorted, stdev)) # like R "pnorm"  # Get the largest index for which p <= m*q  indices = np.nonzero(p <= m * q)[0]  if len(indices):  T = x_sorted[indices[-1]]  else:  logging.debug(  "No passing p-values: min p=%.4g, min m=%.4g, q=%s", p[0], m[0], q  )  T = x_sorted[0] + 1e-16 # ~= 2^-52, like MATLAB "eps"  return T | def FDRThres(x, q, stdev):  """False discovery rate (FDR) threshold."""  M = len(x)  if M < 2:  return 0   m = np.arange(1, M + 1) / M  x_sorted = np.sort(np.abs(x))[::-1]  p = 2 * (1 - stats.norm.cdf(x_sorted, loc=0, scale=stdev)) # like R "pnorm"  # Get the largest index for which p <= m*q  indices = np.nonzero(p <= m * q)[0]  if len(indices):  T = x_sorted[indices[-1]]  else:  logging.debug(  "No passing p-values: min p=%.4g, min m=%.4g, q=%s", p[0], m[0], q  )  T = x_sorted[0] + 1e-16 # ~= 2^-52, like MATLAB "eps"  return T | assert calculateFDRThreshold([0.1, 0.2, 0.3], 0.05,0.05) == 0.1 assert calculateFDRThreshold([-0.1, -0.2, -0.3], 0.05,0.05) == 0.1  assert calculateFDRThreshold([0.1, 0.2, 0.3], 0.05,0) == 0.3000000000000001 assert calculateFDRThreshold([0.1, 0.2, 0.3], 0.05,1) == 0.3000000000000001 assert calculateFDRThreshold([0.1, 0.2, 0.3, 0.4, 0.5], 0.05,0.1) == 0.3  assert calculateFDRThreshold([0.5, 0.4, 0.3, 0.2, 0.1], 0.05,0.1) == 0.3  assert calculateFDRThreshold([1.0, 2.0, 3.0], 0.05,0.5) == 1.0 assert calculateFDRThreshold([1.0, 2.0, 3.0], 0.05,0.9) == 2.0 | calculateFDRThreshold([0.1, 0.2, 0.3], 0.05,0.05) == 0.3000000000000001 calculateFDRThreshold([-0.1, -0.2, -0.3], 0.05,0.05) == 0.3000000000000001 calculateFDRThreshold([0.1, 0.2, 0.3], 0.05,0) == 0.3000000000000001 calculateFDRThreshold([0.1, 0.2, 0.3], 0.05,1) == 0.3000000000000001 calculateFDRThreshold([0.1, 0.2, 0.3, 0.4, 0.5], 0.05,0.1) == 0.5000000000000001 calculateFDRThreshold([0.5, 0.4, 0.3, 0.2, 0.1], 0.05,0.1) == 0.5000000000000001 calculateFDRThreshold([1.0, 2.0, 3.0], 0.05,0.5) == 3.0 calculateFDRThreshold([1.0, 2.0, 3.0], 0.05,0.9) == 3.0 | When stdev ≠ 1 and the parameter is mistakenly interpreted as loc (mean) rather than scale (standard deviation) due to incorrect argument positioning, the computed p-value will be incorrect, leading to a deviation in the resulting threshold. | high | Parameter passing errors |
| PEcnv | def EWMA_SEG(breakpointSelects):  segs = []  start = breakpointSelects[0]  itm = 1  edgeSize = 20  for i in range(len(breakpointSelects)):  if i + 1 < len(breakpointSelects):  if itm == 1:  start = breakpointSelects[i]  itm += 1  if breakpointSelects[i + 1] - breakpointSelects[i] < edgeSize:  if i + 1 == len(breakpointSelects) - 1:  if start - edgeSize > 0:  segs.append([start - edgeSize, breakpointSelects[i + 1] + edgeSize])  else:  segs.append([start, breakpointSelects[i + 1]])  elif breakpointSelects[i + 1] - breakpointSelects[i] > edgeSize:  end = breakpointSelects[i]  if start - edgeSize < 0:  segs.append([start, end + edgeSize])  else:  segs.append([start - edgeSize, end + edgeSize])  itm = 1  return segs | def EWMA_SEG(breakpointSelects):  edgeSize = 20  segments = []  current_segment = []  previous = None   for bp in breakpointSelects:  if previous is None:  current_segment.append(bp)  else:  if bp - previous < edgeSize:  current_segment.append(bp)  else:  segments.append(current_segment)  current_segment = [bp]  previous = bp   if current_segment:  segments.append(current_segment)   expanded_segments = []  for seg in segments:  start = seg[0]  end = seg[-1]  if start - edgeSize > 0:  expanded_segments.append([start - edgeSize, end + edgeSize])  else:  expanded_segments.append([start, end + edgeSize])   return expanded_segments | assert EWMA_SEG([100, 105, 112, 180])==[[80, 132], [160, 200]] assert EWMA_SEG([10, 100, 300])==[[10, 30], [80, 120], [280, 320]] | EWMA_SEG([100, 105, 112, 180])== [[80, 132]] EWMA_SEG([10, 100, 300])== [[10, 30], [80, 120]] | The original version only processes the last segment if the distance between the final two points is less than the edgeSize. If the distance is larger, the last segment gets discarded. In contrast, the new version avoids isolated breakpoints and ensures that the final segment is always properly handled. | high | Boundary handling errors |
| PEcnv | def breakpoint_select(dfArr, sp, upline, dowline):  index = 0  breakpoints = []  for i in dfArr.ewm(span=sp).mean():  if i > upline or i < dowline:  breakpoints.append(index)  index += 1  return breakpoints | def breakpoint_select(dfArr, sp, upline, dowline):  index = 0  breakpoints = []  for i in dfArr.ewm(span=sp, adjust=False).mean():  if i > upline or i < dowline:  breakpoints.append(index)  index += 1  return breakpoints | assert breakpoint_select([1, 2, 3], 19, 3, 2)==[0, 1, 2, 3, 4] | breakpoint_select([1, 2, 3], 19, 3, 2)== [0,1,4] | The only difference between the two functions is the adjust parameter in the exponential weighted moving average (ewm) calculation. The first uses the default adjust=True, which computes the weighted mean by applying weights to all historical data points normalized over the entire window. The second uses adjust=False, performing a recursive calculation that places more weight on recent data points and is computationally more efficient. This results in subtly different smoothing behavior, especially near the start of the data series. | high | Parameter passing errors |
| PEcnv | def adajustedEdge(index , raw_depth):  if index <=1:  return raw_depth[index]  return (1-a)*adajustedEdge(index-1,raw_depth) + a*raw_depth[index] | def adjustedEdge(rawDepths, a):  n = len(rawDepths)  if n == 0:  return []    adjustedDepths = [0] * n  adjustedDepths[0] = rawDepths[0] # Initialize the first adjusted depth   for i in range(1, n):  adjustedDepths[i] = a * rawDepths[i] + (1 - a) * adjustedDepths[i - 1]    return adjustedDepths | assert adjustedEdge([10, 20], 0.5) == '[10, 15.0]' assert adjustedEdge([10, 20, 30], 0.5) == '[10, 15.0, 22.5]' assert adjustedEdge([10, 20, 30], 0) == '[10, 10, 10]' assert adjustedEdge([1, 2, 3, 4, 5], 0.1) == '[1, 1.1, 1.29, 1.561, 1.9049]' assert adjustedEdge([100, 200, 300], 0.75) == '[100, 175.0, 268.75]' | adjustedEdge([10, 20], 0.5) == [10,20] adjustedEdge([10, 20, 30], 0.5) == [10, 20, 25.0] adjustedEdge([10, 20, 30], 0) == [10, 20, 20] adjustedEdge([1, 2, 3, 4, 5], 0.1) == [1, 2, 2.1, 2.29, 2.561] adjustedEdge([100, 200, 300], 0.75) == [100, 200, 275.0] | The first function adajustedEdge computes the exponentially weighted moving average (EWMA) recursively for a single index. While conceptually straightforward, it is inefficient due to redundant computations and deep recursion, making it unsuitable for large inputs. The second function adjustedEdge implements an iterative version of EWMA across the entire array, providing better performance and practical usability. It is preferred in real-world scenarios where the full smoothed series is needed. | medium | alternative implementation |
| PEcnv | def csvstring(text):  return tuple(map(float, text.split(","))) | def csvstring(inputString: str) -> tuple:  if not inputString:  return ()  valuesList = inputString.split(',')  floatList = []  for value in valuesList:  floatValue = float(value)  floatList.append(floatValue)  return tuple(floatList) | assert csvstring("") == () | csvstring("") raises error | Both functions convert a comma-separated string into a tuple of floats. The first version is concise and efficient but lacks safeguards — it will raise an exception if the input is empty or improperly formatted. The second version is more verbose but robust: it explicitly handles empty input and is easier to extend for validation or error handling. For production code or uncertain input formats, the second implementation is generally preferred. | low |  |
| PEcnv | def selectingSegments(breakpoint_select):  segs = []  start = breakpoint_select[0]  itm = 1  for i in range(len(breakpoint_select)):  if i + 1 < len(breakpoint_select):  if itm == 1:  start = breakpoint_select[i]  itm += 1  if breakpoint_select[i + 1] - breakpoint_select[i] < 20:  if i + 1 == len(breakpoint_select) - 1:  if start - 2 > 0:  segs.append([start - 2, breakpoint_select[i + 1] + 2])  else:  segs.append([start, breakpoint_select[i + 1]])  elif breakpoint_select[i + 1] - breakpoint_select[i] > 10:  end = breakpoint_select[i]  if start - 2 < 0:  segs.append([start, end + 2])  else:  segs.append([start - 2, end + 2])  itm = 1  return segs | def selectingSegments(breakpoints):  segments = []  start = breakpoints[0]  end = breakpoints[0]   for i in range(1, len(breakpoints)):  distance = breakpoints[i] - breakpoints[i - 1]    if distance < 20:  end = breakpoints[i]  elif distance > 10:  segments.append((start - 2, end + 2))  start = breakpoints[i]  end = breakpoints[i]   segments.append([start - 2, end + 2])   return segments | assert selectingSegments([15])==[(13,17)] assert selectingSegments([10, 30])==[(8, 12), (28, 32)] | selectingSegments([15])== [] selectingSegments([10, 30])== [[8,12]] | The two functions serve the same purpose: to select and group breakpoints into segments based on their proximity. However, the first version is overly complex, with deeply nested conditionals and unclear variable names (itm, li, etc.). It also has inconsistent edge handling, which can lead to missed or incorrect segments. The second version is significantly cleaner and more robust. It uses well-named variables, avoids unnecessary branching, handles the final segment explicitly, and is easier to read and maintain. | high | Boundary handling errors |
| PEcnv | def gapper_scale(a):  gaps = np.diff(np.sort(a))  n = len(a)  idx = np.arange(1, n)  weights = idx * (n - idx)  return (gaps * weights).sum() * np.sqrt(np.pi) / (n * (n - 1)) | def gapper_scale(values):  n = len(values)  if n < 2:  return 0   sorted_values = sorted(values)  gaps = [0] * (n - 1)   for i in range(n - 1):  gaps[i] = sorted_values[i + 1] - sorted_values[i]   weighted_gaps = [0] * (n - 1)   for i in range(n - 1):  weight = (i + 1) * (n - (i + 1))  weighted_gaps[i] = gaps[i] * weight   sum_weighted_gaps = 0  for i in range(n - 1):  sum_weighted_gaps += weighted_gaps[i]   scaling_factor = (sum_weighted_gaps * (3.141592653589793) ** 0.5) / (n * (n - 1))   return scaling_factor | assert gapper_scale([1]) == 0 | gapper_scale([1]) == nan | Both functions implement the same statistical measure: the gapper scale estimator, which assesses the dispersion of a dataset based on the gaps between sorted values. The first version uses NumPy and is highly efficient and concise, making it suitable for numerical computing tasks. The second version is a pure Python implementation, which is more verbose but easier to follow, especially for educational or demonstrative purposes. It also includes a boundary check for small input sizes, which is absent in the NumPy version. | low |  |
| PEcnv | def median_absolute_deviation(a, scale_to_sd=True):  a_median = np.median(a)  mad = np.median(np.abs(a - a_median))  if scale_to_sd:  mad *= 1.4826  return mad | def median_absolute_deviation(array, scale):  n = len(array)  if n == 0:  return 0   sortedArray = sorted(array)  median = FINDMedian(sortedArray)   absoluteDeviations = [0] * n  for i in range(n):  absoluteDeviations[i] = ABSOLUTE(sortedArray[i] - median)   mad = FINDMedian(absoluteDeviations)   if scale:  mad = mad * 1.4826   return mad  def FINDMedian(sortedArray):  n = len(sortedArray)  if n % 2 == 1:  return sortedArray[n // 2]  else:  return (sortedArray[n // 2 - 1] + sortedArray[n // 2]) / 2  def ABSOLUTE(value):  if value < 0:  return -value  else:  return value | assert median_absolute_deviation([], False) == 0 assert median_absolute_deviation([1, 2, 3, 4], False) == 1.5 assert median_absolute_deviation([1, 2, 3, 4, 5], False) == 2.0 | median_absolute_deviation([], False) == nan median_absolute_deviation([1, 2, 3, 4], False) == 1.0 median_absolute_deviation([1, 2, 3, 4, 5], False) == 2.9652 | Both functions compute the Median Absolute Deviation (MAD), a robust measure of statistical dispersion. The first version uses NumPy's vectorized operations, making it concise and efficient, ideal for production use with large datasets. The second version is a pure Python implementation with custom functions for median and absolute value, making it more verbose and slower but easier to understand for educational purposes. It also explicitly handles the empty input case, returning 0, which the NumPy version does not handle by default. | medium | alternative implementation |
| PEcnv | def weighted_std(a, weights):  mean = np.average(a, weights=weights)  var = np.average((a - mean) ** 2, weights=weights)  return np.sqrt(var) | def weighted_std(LogR_values, weights):  n = len(LogR_values)    if n == 0:  return 0    weighted_mean = 0.0  sum_weights = 0.0  sum_weighted_values = 0.0  sum_weighted_squared_values = 0.0    for i in range(n):  sum_weights += weights[i]  sum_weighted_values += (LogR_values[i] * weights[i])  sum_weighted_squared_values += (LogR_values[i] * LogR_values[i] * weights[i])    if sum_weights == 0:  return 0    weighted_mean = sum_weighted_values / sum_weights    variance = (sum_weighted_squared_values / sum_weights) - (weighted_mean * weighted_mean)    return variance ** 0.5 | assert weighted_std([1.0, 2.0, 3.0], [1, 1, 1]) == '0.8164965809277263' assert weighted_std([1.0, 2.0, 3.0], [1, 2, 3]) == '0.7453559924999291' | weighted_std([1.0, 2.0, 3.0], [1, 1, 1]) == 0.816496580927726 weighted_std([1.0, 2.0, 3.0], [1, 2, 3]) == 0.7453559924999298 | Both functions compute the weighted standard deviation of a dataset. The first version uses NumPy's average function for both the mean and variance, resulting in a concise and efficient implementation. The second version is a fully manual approach using Python loops, making each computation step explicit. While slower, it is easier to read, debug, and handles corner cases like empty input or zero total weights more gracefully. For performance-sensitive applications, the NumPy version is preferable; for learning or environments without NumPy, the manual version is more portable. | low |  |
| PEcnv | def p_adjust_bh(p):  p = np.asfarray(p)  by_descend = p.argsort()[::-1]  by_orig = by_descend.argsort()  steps = float(len(p)) / np.arange(len(p), 0, -1)  q = np.minimum(1, np.minimum.accumulate(steps * p[by_descend]))  return q[by_orig] | def p_adjust_bh(p):  p = np.asfarray(p)  by_descend = p.argsort()  by_orig = by_descend.argsort()  steps = float(len(p)) / np.arange(len(p), 0, -1)  q = np.minimum(1, np.minimum.accumulate(steps * p[by_descend]))  return q[by_orig] | assert p_adjust_bh([0.05, 0.01, 0.03, 0.02]) == [0.01, 0.01, 0.01, 0.01] assert p_adjust_bh([0.1, 0.2, 0.3, 0.4]) == [0.1, 0.1, 0.1, 0.1] assert p_adjust_bh([0.5, 0.5, 0.5, 0.5]) == [0.5, 0.5, 0.5, 0.5] assert p_adjust_bh([0.0, 0.0, 0.0, 0.0]) == [0.0, 0.0, 0.0, 0.0] assert p_adjust_bh([0.9, 0.8, 0.7, 0.6]) == [0.6, 0.6, 0.6, 0.6] assert p_adjust_bh([0.2]) == [0.2] assert p_adjust_bh([0.99, 0.01]) == [0.01, 0.01] assert p_adjust_bh([0.25, 0.75, 0.5]) == [0.25, 0.25, 0.25] assert p_adjust_bh([0.3, 0.6, 0.1, 0.4, 0.2]) == [0.1, 0.1, 0.1, 0.1, 0.1] assert p_adjust_bh([0.4, 0.4, 0.4, 0.4, 0.4]) == [0.4, 0.4, 0.4, 0.4, 0.4] | p_adjust_bh([0.05, 0.01, 0.03, 0.02]) == [0.05, 0.039999999999999994, 0.039999999999999994, 0.039999999999999994] p_adjust_bh([0.1, 0.2, 0.3, 0.4]) == [0.39999999999999997, 0.39999999999999997, 0.39999999999999997, 0.4] p_adjust_bh([0.5, 0.5, 0.5, 0.5]) == [0.5, 0.5, 0.5, 0.5] p_adjust_bh([0.0, 0.0, 0.0, 0.0]) == [0.0, 0.0, 0.0, 0.0] p_adjust_bh([0.9, 0.8, 0.7, 0.6]) == [0.9, 0.9, 0.9, 0.9] p_adjust_bh([0.2]) == [0.2] p_adjust_bh([0.99, 0.01]) == [0.99, 0.02] p_adjust_bh([0.25, 0.75, 0.5]) == [0.75, 0.75, 0.75] p_adjust_bh([0.3, 0.6, 0.1, 0.4, 0.2]) == [0.5, 0.6, 0.5, 0.5, 0.5] p_adjust_bh([0.4, 0.4, 0.4, 0.4, 0.4]) == [0.4, 0.4, 0.4, 0.4, 0.4] | The only difference between the two versions lies in how the p-values are sorted: the first version sorts them in descending order ([::-1]), while the second sorts them in ascending order. For the Benjamini–Hochberg procedure to work correctly, the p-values must be sorted in ascending order before computing the adjusted q-values via cumulative minima. Therefore, the second version is correct, and the first version is incorrect. | medium | domain knowledge violation |
| PEcnv | def segment_mean(log2_values, weights, skip_low=False):  if skip_low:  log2_values = [x for x in log2_values if x is not None]  if len(log2_values) == 0:  return np.nan  if len(weights) > 0 and any(weights):  return np.average(log2_values, weights=weights)  return np.mean(log2_values) | def segment_mean(cnarr, weights, skip_low_coverage):  if len(cnarr) == 0:  return float('nan')   if skip_low_coverage:  filtered_cnarr = []  for i in range(len(cnarr)):  if cnarr[i] is not None: # Assuming 'LOW COVERAGE' is represented by None  filtered_cnarr.append(cnarr[i])  cnarr = filtered_cnarr   if len(cnarr) == 0:  return float('nan')   if len(weights) > 0:  weighted_sum = 0  total_weight = 0  for i in range(len(cnarr)):  weighted_sum += cnarr[i] * weights[i]  total_weight += weights[i]  return weighted_sum / total_weight  else:  total_sum = 0  for i in range(len(cnarr)):  total_sum += cnarr[i]  return total_sum / len(cnarr) | assert segment_mean([], [], False) == np.nan assert segment_mean([1, 2, None, 4], [0.1, 0.2, 0.3, 0.4], True) == 2.833333333333333  assert segment_mean([None, None], [0.1, 0.2], True) == np.nan | segment_mean([], [], False) raises error segment_mean([1, 2, None, 4], [0.1, 0.2, 0.3, 0.4], True) raises error  segment_mean([None, None], [0.1, 0.2], True) raises error | Both functions compute the mean of a list of log2 values, optionally weighted and filtered to exclude low-coverage entries (represented as None). The first version uses NumPy's built-in functions (np.average, np.mean) for concise and efficient calculation, while the second version is a pure Python implementation with manual loops. Functionally, the NumPy version is faster but cannot handle Nan inputs. | low |  |
| PEcnv | def first_of(elems):  """Return the first element of the input."""  return elems[0] | def first_of(elems):  """Return the first element of the input."""  if len(elems) == 0:  return None  else:  return elems[0] | assert first_of([]) == None | first_of([]) raises error | The first version assumes that the input list elems is non-empty and directly returns the first element. If the list is empty, it raises an IndexError. The second version adds a safety check to return None if the list is empty, making it more robust and suitable for use in contexts where the input may be empty or unknown. | low |  |
| PEcnv | def last_of(elems):  """Return the last element of the input."""  return elems[-1] | def last_of(elems):  """Return the last element of the input."""  if len(elems) > 0:  return elems[-1]  else:  return None | assert last_of([]) == None | last_of([]) raises error | The first version assumes that the input list elems is non-empty and directly returns the first element. If the list is empty, it raises an IndexError. The second version adds a safety check to return None if the list is empty, making it more robust and suitable for use in contexts where the input may be empty or unknown. | low |  |
| PEcnv | def merge_strands(elems):  strands = set(elems)  if len(strands) > 1:  return '.'  return elems[0] | def merge_strands(elems):  if not elems:  return ""    unique_strands = set(elems)    if len(unique_strands) > 1:  return "."  else:  return next(iter(unique_strands)) | assert merge_strands([]) == '' | merge_strands([]) raises error | The first version assumes that the input list elems is non-empty and directly returns the first element. If the list is empty, it raises an IndexError. The second version adds a safety check to return None if the list is empty, making it more robust and suitable for use in contexts where the input may be empty or unknown. | low |  |
| CNVpytor(314) | def segments_decode(aseg):  """  Decode segments.   Parameters  ----------  aseg : numpy.ndarray   Returns  -------  segments : list of list of int   """  max = 2 ** 32 - 1  segments = []  l = []  for x in list(aseg):  if x == max:  segments.append(l)  l = []  else:  l.append(x)  return segments | def segments_decode(aseg):  """  Decode segments.   Parameters  ----------  aseg : numpy.ndarray   Returns  -------  segments : list of list of int   """  max_value = 2 ** 32 - 1  segments = []  current_segment = []    for value in aseg:  if value == max_value:  if current_segment:  segments.append(current_segment)  current_segment = []  else:  current_segment.append(value)   if current_segment:  segments.append(current_segment)   return segments | assert segments_decode(np.array([1, 2, 3, 4294967295, 4, 5])) == [[1, 2, 3], [4, 5]] assert segments_decode(np.array([4294967295, 1, 2, 3])) == [[1, 2, 3]] assert segments_decode(np.array([1, 2, 3])) == [[1, 2, 3]] assert segments_decode(np.array([4294967295])) == [] assert segments_decode(np.array([])) == [] assert segments_decode(np.array([4294967295, 4294967295, 1, 2])) == [[1, 2]] | segments_decode(np.array([1, 2, 3, 4294967295, 4, 5])) = [[1, 2, 3]] segments_decode(np.array([4294967295, 1, 2, 3])) = [[]] segments_decode(np.array([1, 2, 3])) = [] segments_decode(np.array([4294967295])) = [[]] segments_decode(np.array([])) = [] segments_decode(np.array([4294967295, 4294967295, 1, 2])) = [[], []] | 1. Handling of empty segments  First version: Appends an empty list whenever a separator (2**32 - 1) appears consecutively or at the beginning, resulting in spurious empty segments.  Second version: Skips empty segments by checking whether the current segment contains any values before appending.  2. Handling of the final segment  First version: Fails to include the last segment if the input does not end with a separator.  Second version: Always appends the final segment after the loop, ensuring no data is lost.  3. Robustness and correctness  First version: Works only if the encoded array strictly ends with a separator and does not contain consecutive delimiters.  Second version: Robust against missing or extra delimiters, producing consistent, valid output even in edge cases. | low |  |
| CNVpytor(314) | def normal(x, a, x0, sigma):  """  Normal distribution.   Parameters  ----------  x : float  Variable.  a : float  Area.  x0 : float  Mean value.  sigma : float  Sigma.   Returns  -------  : float  Value of distribution in x.   """  return a * np.exp(-(x - x0) ** 2 / (2 * sigma ** 2)) / np.sqrt(2 * np.pi) / sigma | def normal_distribution(x, a, x0, sigma):  """  Normal distribution.   Parameters  ----------  x : float  Variable.  a : float  Area.  x0 : float  Mean value.  sigma : float  Sigma.   Returns  -------  : float  Value of distribution in x.   """  pi = 3.141592653589793  exponent = -((x - x0) * (x - x0)) / (2 * sigma * sigma)  coefficient = a / (sigma * (2 * pi) ** 0.5)  return coefficient * (2.718281828459045 ** exponent) | assert normal_distribution(0, 1, 0, 1) == '0.3989422804014327' assert normal_distribution(1, 1, 0, 1) == '0.24197072451914337' assert normal_distribution(0, 2, 0, 1) == '0.7978845608028654' assert normal_distribution(2, 1, 1, 1) == '0.24197072451914337' assert normal_distribution(-1, 1, 0, 2) == '0.17603266338214976' assert normal_distribution(1, 1, 1, 0.5) == '0.7978845608028654' assert normal_distribution(1, 0, 0, 1) == '0.0' assert normal_distribution(1, 1, 1, 1e-10) == '3989422804.0143275' | normal_distribution(1, 1, 1, 1e-10) = 3989422804.014327 | Both compute the normal distribution function and are mathematically equivalent, but they differ in precision, and how constants (e and π) are handled. | medium | alternative implementation |
| CNVpytor(314) | def lognormal(x, a, x0, sigma):  """  Log normal distribution.   Parameters  ----------  x : float  Variable.  a : float  Area.  x0 : float  Mean value.  sigma : float  Sigma.   Returns  -------  : float  Value of distribution in x.   """  return np.log(a) - (x - x0) ** 2 / (2 * sigma ** 2) - np.log(np.sqrt(2 * np.pi) / sigma) | def LogNormalDistribution(x, a, x0, sigma):  """  Log normal distribution.   Parameters  ----------  x : float  Variable.  a : float  Area.  x0 : float  Mean value.  sigma : float  Sigma.   Returns  -------  : float  Value of distribution in x.   """  exponent = 0.0  normalizationFactor = 0.0  logNormalValue = 0.0   normalizationFactor = a / (x * sigma * (2 * 3.141592653589793)**0.5)  exponent = -((log(x) - x0) ** 2) / (2 * sigma ** 2)  logNormalValue = normalizationFactor * (2.718281828459045 ** exponent)   return logNormalValue | assert LogNormalDistribution(1.0, 1.0, 0.0, 1.0) == 0.3989422804014327 #-1.4189385332046727 assert LogNormalDistribution(2.0, 1.0, 0.0, 1.0) == 0.15687401927898112 #-2.9189385332046727 assert LogNormalDistribution(1.0, 2.0, 0.0, 1.0) == 0.7978845608028654 #-0.7257913526447274 assert LogNormalDistribution(1.0, 1.0, 1.0, 1.0) == 0.24197072451914337 #-0.9189385332046727 assert LogNormalDistribution(1.0, 1.0, 0.0, 0.5) == 0.7978845608028654 #-3.6120857137646176 assert LogNormalDistribution(3.0, 1.0, 0.0, 2.0) == 0.057179111975974625 #-1.3507913526447273 | LogNormalDistribution(1.0, 1.0, 0.0, 1.0) = -1.4189385332046727 LogNormalDistribution(2.0, 1.0, 0.0, 1.0) = -2.9189385332046727 LogNormalDistribution(1.0, 2.0, 0.0, 1.0) = -0.7257913526447274 LogNormalDistribution(1.0, 1.0, 1.0, 1.0) = -0.9189385332046727 LogNormalDistribution(1.0, 1.0, 0.0, 0.5) = -3.6120857137646176 LogNormalDistribution(3.0, 1.0, 0.0, 2.0) = -1.3507913526447273 | The two implementations are not functionally equivalent. The first version returns the logarithm of a normal distribution evaluated at `x` without applying a logarithmic transformation to the input, effectively representing a log-scaled value of a regular normal distribution rather than a true log-normal distribution. In contrast, the second version correctly implements the standard log-normal probability density function: it applies `log(x)` in the exponent, includes the proper `1/(x σ √2π)` normalization factor, and returns the probability density value itself. Thus, the first version outputs a log-scale value of a normal distribution, while the second outputs the actual log-normal density, making their meanings and results fundamentally different. | medium | alternative implementation |
| CNVpytor(314) | def t_test_1_sample(mean, m, s, n):  if s == 0:  s = 1  t = (mean - m) / s * np.sqrt(n)  p = 1.0 - stats.t.cdf(np.abs(t), df=n - 1)  return p | def one_sample_t_test(sample, population_mean):  n = len(sample)  if n < 2:  return "Sample size must be at least 2"    mean = sum(sample) / n  s = (sum((x - mean) ** 2 for x in sample) / (n - 1)) ** 0.5    if s == 0:  return "Standard deviation is zero, cannot perform t-test"    t = (mean - population_mean) / (s / (n ** 0.5))  degrees_of_freedom = n - 1    p_value = 2 * (1 - stats.t.cdf(abs(t), df=degrees_of_freedom))    return t, p_value | assert one_sample_t_test([1, 2], 0) == 0.20483276469913347 #0.10241638234956674 assert one_sample_t_test([1, 2], 1) == 0.49999999999999956 #0.24999999999999978 assert one_sample_t_test([1, 2, 3], 2) == 1.0 #0.5 assert one_sample_t_test([5, 5, 5], 5) == "'Standard deviation is zero, cannot perform t-test'" assert one_sample_t_test([1, 2, 3, 4, 5], 3) == 1 #0.5 assert one_sample_t_test([3, 3, 3, 3, 3], 3) == "'Standard deviation is zero, cannot perform t-test'" | one_sample_t_test([1, 2], 0) = 0.10241638234956674 one_sample_t_test([1, 2], 1) = 0.24999999999999978 one_sample_t_test([1, 2, 3], 2) = 0.5 one_sample_t_test([1, 2, 3, 4, 5], 3) = 0.5 | The two versions differ mainly in the calculation and interpretation of the p-value. The first version computes a one-sided p-value using 1 - CDF(\|t\|), whereas the second version computes a two-sided p-value with 2 * (1 - CDF(\|t\|)), which is the standard approach for a one-sample t-test. Additionally, the second version includes a check for a minimum sample size (n >= 2) to ensure the test is valid, while the first version does not. | medium | domain knowledge violation |
| CNVpytor(314) | def likelihood_pixels_pval(likelihood):  """  Calculates maximum likelihood pixel positions and p-value.  Parameters  ----------  likelihood   Returns  -------  b : float  BAF level (difference from 1/2)  p : float  p-value for event different than 1/2   """  res = likelihood.size  max_lh = np.amax(likelihood)  ix = np.where(likelihood == max_lh)[0][0]  if ix > res // 2:  ix = res - 1 - ix  ix1 = (res // 2 + ix) // 2  ix2 = res - 1 - ix1  p = np.sum(likelihood[ix1:ix2]) / np.sum(likelihood)  return ix, res - 1 - ix, p | def max_likelihood_and_p_value(likelihood_array):  n = len(likelihood_array)  max_index = 0  max_value = likelihood_array[0]    for i in range(1, n):  if likelihood_array[i] > max_value:  max_value = likelihood_array[i]  max_index = i    midpoint = n / 2  if max_index < midpoint:  ix1 = max_index  ix2 = min(max_index + (n / 4), n - 1)  else:  ix1 = max(0, max_index - (n / 4))  ix2 = max_index    p_value_sum = 0  for i in range(int(ix1), int(ix2) + 1):  p_value_sum += likelihood_array[i]    total_sum = sum(likelihood_array)  p_value = p_value_sum / total_sum    return max_index, n - max_index - 1, p_value | assert max_likelihood_and_p_value(np.array([0.1, 0.2, 0.3, 0.4, 0.5])) == (4, 0, 0.7999999999999999) #(0, 4, 0.3333333333333333) assert max_likelihood_and_p_value(np.array([0.5, 0.4, 0.3, 0.2, 0.1])) == (0, 4, 0.6) #(0, 4, 0.4666666666666666) assert max_likelihood_and_p_value(np.array([0.1, 0.1, 0.1, 0.1, 0.1])) == (0, 4, 0.4) assert max_likelihood_and_p_value(np.array([0.5, 0.5, 0.5, 0.5, 0.5])) == (0, 4, 0.4) assert max_likelihood_and_p_value(np.array([0.1, 0.2, 0.3, 0.4, 0.5, 0.6])) == (5, 0, 0.7142857142857143) #(0, 5, 0.42857142857142855) assert max_likelihood_and_p_value(np.array([0.6, 0.5, 0.4, 0.3, 0.2, 0.1])) == (0, 5, 0.5238095238095238) #(0, 5, 0.5714285714285714) assert max_likelihood_and_p_value(np.array([0.1, 0.2, 0.3, 0.4, 0.5, 0.5])) == (4, 1, 0.6) #(1, 4, 0.15) assert max_likelihood_and_p_value(np.array([0.5, 0.5, 0.5, 0.5, 0.5, 0.6])) == (5, 0, 0.5161290322580645) #(0, 5, 0.48387096774193544) assert max_likelihood_and_p_value(np.array([0.1])) == (0, 0, 1.0) #(0, 0, 0.0) assert max_likelihood_and_p_value(np.array([0.1, 0.2])) == (1, 0, 1.0) #(1, 0, 0.0) | max_likelihood_and_p_value(np.array([0.1, 0.2, 0.3, 0.4, 0.5])) == (0, 4, 0.3333333333333333) max_likelihood_and_p_value(np.array([0.5, 0.4, 0.3, 0.2, 0.1])) == (0, 4, 0.4666666666666666) max_likelihood_and_p_value(np.array([0.1, 0.2, 0.3, 0.4, 0.5, 0.6])) == (0, 5, 0.42857142857142855) max_likelihood_and_p_value(np.array([0.6, 0.5, 0.4, 0.3, 0.2, 0.1])) == (0, 5, 0.5714285714285714) max_likelihood_and_p_value(np.array([0.1, 0.2, 0.3, 0.4, 0.5, 0.5])) == (1, 4, 0.15) max_likelihood_and_p_value(np.array([0.5, 0.5, 0.5, 0.5, 0.5, 0.6])) == (0, 5, 0.48387096774193544) max_likelihood_and_p_value(np.array([0.1])) == (0, 0, 0.0) max_likelihood_and_p_value(np.array([0.1, 0.2])) == (1, 0, 0.0) | The first version enforces symmetry around the midpoint and computes a p-value based on a balanced likelihood range, while the second version uses a heuristic asymmetric window based on the maximum index. As a result, the computed p values and index pairs will generally differ, especially when the likelihood distribution is skewed. | medium | alternative implementation |
| AlignQC-master(143) | def analyze_x(xval,svals,args):  s = args.samples_per_xval  #cnts = sorted(  # [len(  # [k for k in collections.Counter([z for z in [random.choice(vals) for y in range(0,xval)] if z]).values() if k >= args.min_depth]  # )   # for j in range(0,s)]  # )  cnts = []  for j in range(0,s):  vals = svals[j][0:xval]  cnts.append(len([x for x in collections.Counter([k for k in vals if k]).values() if x >= args.min_depth]))  cnts = sorted(cnts)  lower = float(cnts[int(len(cnts)*0.05)])  mid = median(cnts)  upper = float(cnts[int(len(cnts)*0.95)])  #print len(vals)  #print len([x for x in vals if x >0])  #print cnts[0:5]  #print cnts[-5:]  #print [xval, lower, mid, upper]  return [xval, lower, mid, upper] | def analyze_x(xval, svals, args):  s = args.samples_per_xval  min_depth = args.min_depth  cnts = []   for j in range(s):  vals = svals[j][0:xval]  ctr = collections.Counter(k for k in vals if k is not None)  cnts.append(sum(1 for v in ctr.values() if v >= min_depth))   cnts = np.array(cnts)  return [  xval,  np.percentile(cnts, 5),  np.median(cnts),  np.percentile(cnts, 95),  ] | assert analyze_x(5, [[0,1,1,2,2],[0,0,1,2,3],]) == [5, 1.05, 1.5, 1.95] #class Args: samples_per_xval = 2 min_depth = 2 args = Args() assert analyze_x(4, [[0,0,0,1],[0,0,0,0],[0,1,1,1],]) == [4, 1.1, 2.0, 2.0] | analyze_x(5, [[0,1,1,2,2],[0,0,1,2,3]]) = [5, 0.0, 1.0, 2.0] analyze_x(4, [[0,0,0,1],[0,0,0,0],[0,1,1,1],]) = [4, 0.0, 1, 1.0] | The optimized version improves the original by correctly filtering values using `k is not None` instead of `if k`, which avoids unintentionally discarding valid zeros and ensures accurate counting. It also replaces manual percentile indexing with `np.percentile`, providing more reliable statistical estimates, especially for small sample sizes. In addition, the optimized implementation reduces redundant computations and handles edge cases more robustly, resulting in cleaner, more accurate, and more maintainable code overall. | medium | alternative implementation |
| ClassifyCNV-master(20) | def assign_section3_points(gene_number, cnv_type):  """Assign points for section 3 of the ACMG rubric based on the number of genes included in the CNV.   Args:  gene_number: Number of genes included in the CNV.  cnv_type: DEL or DUP.   Returns:  number_points: Number of points to assign.   """  number_points = 0.0  if cnv_type.upper() == 'DEL':  if gene_number < 25:  number_points = 0.0  elif gene_number < 35:  number_points = 0.45  elif gene_number > 34:  number_points = 0.9  elif cnv_type.upper() == 'DUP':  if gene_number < 35:  number_points = 0.0  elif gene_number < 50:  number_points = 0.45  elif gene_number > 49:  number_points = 0.9  return number_points | def assign_section3_points(gene_number, cnv_type):  cnv_type = cnv_type.upper()    if cnv_type == 'DEL':  if gene_number < 25:  return 0.0  elif gene_number < 35:  return 0.45  else:  return 0.9  elif cnv_type == 'DUP':  if gene_number < 35:  return 0.0  elif gene_number < 50:  return 0.45  else:  return 0.9  else:  raise ValueError(f"Invalid CNV type: {cnv_type}") | assign_section3_points(20, "ABC") raise ValueError | assign_section3_points(20, "ABC") = 0.0 | The main difference between the two versions is how they handle invalid `cnv_type` inputs: the original version silently returns 0.0, while the optimized version raises an error. For valid inputs (`'DEL'` or `'DUP'`), both versions produce the same results. And the optimized version also simplifies and clarifies the branching logic by using <=/< comparisons consistently, removing overlapping conditions and making the thresholds explicit, whereas the original version had redundant checks like elif gene_number > 34. This makes the optimized version more readable and less error-prone. | low |  |
| gatk-sv-main(1076) | def calculate_median_mad(group):  median = np.median(group)  mad = np.median(np.abs(group - median))  return median, mad | def calculate_median_mad(group, scale=True):  group = np.asarray(group)  median = np.nanmedian(group)  mad = np.nanmedian(np.abs(group - median))    if scale:  mad *= 1.4826 # scale MAD to be comparable to std for normal distribution    return median, mad | assert calculate_median_mad([1, 2, 3, np.nan, 4, 5]) == (3.0, 1.4826) assert calculate_median_mad([1, 2, 3, 4, 5]) == (3.0, 1.4826) assert calculate_median_mad([np.nan, 7, 8, 9, np.nan, 10]) == (8.5, 1.4826) assert calculate_median_mad([np.nan, np.nan, np.nan]) == (nan, nan) assert calculate_median_mad([5, np.nan]) == (5.0, 0.0) | calculate_median_mad([1, 2, 3, np.nan, 4, 5]) = (nan, nan) calculate_median_mad([1, 2, 3, 4, 5]) = (3.0, 1.0) calculate_median_mad([np.nan, 7, 8, 9, np.nan, 10]) = (nan, nan) calculate_median_mad([5, np.nan]) = (nan, nan) | The original version computes the median and MAD directly, without handling NaN values and without scaling MAD. The optimized version improves robustness by ignoring NaNs instead of propagating them, and increases statistical correctness by applying the standard MAD scaling factor (1.4826). As a result, the optimized version produces stable, interpretable results even with missing data and aligns with standard robust-statistics definitions. | low |  |
| gatk-sv-main(1076) | def weighted_avg_and_std(values, weights):  """Return the weighted average and standard deviation for a given array of values, and weights.  Copied from: https://stackoverflow.com/questions/2413522/weighted-standard-deviation-in-numpy  """  average = np.average(values, weights=weights)  # Fast and numerically precise:  variance = np.average((values - average) ** 2, weights=weights)  return average, np.sqrt(variance) | def weighted_avg_and_std(values, weights):  import numpy as np   values = np.asarray(values, dtype=float)  weights = np.asarray(weights, dtype=float)   # Remove NaN  mask = ~np.isnan(values) & ~np.isnan(weights)  values = values[mask]  weights = weights[mask]   if len(values) == 0 or np.sum(weights) == 0:  return np.nan, np.nan   average = np.average(values, weights=weights)  variance = np.average((values - average) ** 2, weights=weights)   return average, np.sqrt(variance) | assert weighted_avg_and_std([1, 2, np.nan, 3], [1, 1, 1, 1]) == (2.0, 0.816496580927726) assert weighted_avg_and_std([1, 2, 3], [0, 0, 0]) == (nan, nan) assert weighted_avg_and_std([np.nan, 3, np.nan], [1, 2, 0]) == (3.0, 0.0) assert weighted_avg_and_std([10, 20, 30], [1, -2, 1]) == (nan, nan) | weighted_avg_and_std([1, 2, np.nan, 3], [1, 1, 1, 1]) = (nan, nan) weighted_avg_and_std([1, 2, 3], [0, 0, 0]) raises ZeroDivisionError weighted_avg_and_std([np.nan, 3, np.nan], [1, 2, 0]) = (nan, nan) weighted_avg_and_std([10, 20, 30], [1, -2, 1]) raises ZeroDivisionError | The original implementation correctly computes the weighted mean and population weighted standard deviation, but it lacks robustness: it does not handle NaN values, zero-sum weights, or sample-standard-deviation scenarios. A more complete version should filter NaNs, guard against zero weights, and optionally support unbiased (sample) weighted variance. | low |  |
| gatk-sv-main(1076) | def convert_poisson_p(log_pval):  """  Convert poisson p-value to count, assuming 0 background   Can't use ss.poisson.ppf because k/mu are flipped in pe/sr test calculation  """  count = 0  while True:  pval_cmp = -np.log10(ss.poisson.cdf(0, count))   if pval_cmp > log_pval:  return max(1, count - 1)   count += 1 | def convert_poisson_p(log_pval):  """Convert -log10(p) of Poisson(λ) at X=0 to λ."""  if log_pval < 0:  raise ValueError("log_pval must be >= 0")   lam = log_pval * np.log(10)  return max(1, int(lam)) | assert convert_poisson_p(-0.1) == 1 | convert_poisson_p(-0.1) raise ValueError | The optimized version is much faster because it avoids the iterative while-loop in the original version. The original computes the Poisson CDF repeatedly, incrementing `count` until a condition is met, which can take many iterations. In contrast, the optimized version uses a direct mathematical transformation (`λ ≈ log_pval * ln(10)`), yielding the result immediately without repeated computation. This makes it far more efficient, especially for large numbers of calls or larger `log_pval` values. And the main difference between the two versions is how they handle negative `log_pval` values. The original version implicitly returns 1 for any negative input, while the optimized version explicitly raises a `ValueError` for `log_pval < 0`. For non-negative inputs, both versions produce equivalent results. | low |  |
| gatk-sv-main(1076) | def extract_non_ref_samples(pin, sample_name):  GT_POS = pin[8].split(':').index('GT')  sample_GT = [i.split(':')[GT_POS] for i in pin[9:]]  sample_out = [i for i in sample_name[1:]  if not sample_GT[sample_name.index(i) - 1] == '0/0']  return ','.join(sample_out) | def extract_non_ref_samples(pin, sample_name):  try:  GT_POS = pin[8].split(':').index('GT')  except ValueError:  return ''   sample_GT = [i.split(':')[GT_POS] for i in pin[9:]]  sample_out = [name for idx, name in enumerate(sample_name[1:]) if sample_GT[idx] != '0/0' and sample_GT[idx] != './.']  return ','.join(sample_out) | assert extract_non_ref_samples(  pin=['chr1', '100', '100', '.', '.', '.', '.', 'INFO', 'AD:DP', '0:10', '0:12'],  sample_name=['Sample', 'S1', 'S2']) == "" assert extract_non_ref_samples(  pin=['chr1','100','100','.','.','.','.','INFO','GT:DP','0/1:10','./.:12'],  sample_name=['Sample','S1','S2']) == "S1" assert extract_non_ref_samples(  pin=['chr1','100','100','.','.','.','.','INFO','GT:DP','0/0:10','0/0:12'],  sample_name=['Sample','S1','S2']) == "" | extract_non_ref_samples(  pin=['chr1', '100', '100', '.', '.', '.', '.', 'INFO', 'AD:DP', '0:10', '0:12'],  sample_name=['Sample', 'S1', 'S2']) raises ValueError extract_non_ref_samples(  pin=['chr1','100','100','.','.','.','.','INFO','GT:DP','0/1:10','./.:12'],  sample_name=['Sample','S1','S2']) = "S1,S2" | The two versions differ mainly in handling edge cases: the original version can fail or produce incorrect output when the GT field is missing, genotypes are missing (`./.`), or the sample list length mismatches. The optimized version handles these cases robustly, ensuring correct extraction of non-reference samples. Its advantage lies in reliability and safer indexing, avoiding crashes or incorrect results. | low |  |
| gatk-sv-main(1076) | def choose_parent_counts(stats):  # If the children don't agree, pick the strongest  is_child = (stats['sample'].str.endswith('p1') \|  stats['sample'].str.endswith('s1'))  children = stats.loc[is_child]  best_idx = children.groupby('coord')['log_pval'].idxmax()  best = children.loc[best_idx, 'coord pos'.split()].set_index('coord').pos   is_parent = (stats['sample'].str.endswith('fa') \|  stats['sample'].str.endswith('mo'))  match_bestA = (stats.coord == 'posA') & (stats.pos == best.posA)  match_bestB = (stats.coord == 'posB') & (stats.pos == best.posB)   parents = stats.loc[is_parent & (match_bestA \| match_bestB)]  parents = parents.drop_duplicates()   summed = parents.groupby('sample')['called_median bg_median'.split()].sum()  pval = ss.poisson.cdf(summed.bg_median, summed.called_median)  summed['log_pval'] = np.abs(np.log10(pval))   # Add metadata  summed['coord'] = 'sum'  summed['pos'] = 0  summed['name'] = parents.iloc[0]['name']  summed = summed.reset_index()   parents = pd.concat([parents, summed])  stats = pd.concat([children, parents])   return stats | def choose_parent_counts(stats):  """  Select the strongest child positions and compute Poisson p-values for parents.  Robust version: handles empty children/parents, avoids repeated concat, checks keys.  """   # --- 1. Select children ---  is_child = stats['sample'].str.endswith(('p1', 's1'))  children = stats[is_child].copy()   if children.empty:  return stats # no children, return as is   # Pick the strongest child per coordinate  best_idx = children.groupby('coord')['log_pval'].idxmax()  best = children.loc[best_idx, ['coord', 'pos']].set_index('coord')   # --- 2. Select parents matching best child positions ---  is_parent = stats['sample'].str.endswith(('fa', 'mo'))  parents = stats[is_parent].copy()   if parents.empty:  return pd.concat([children, parents], ignore_index=True)   # Make sure positions exist  matched_coords = best.index.intersection(parents['coord'].unique())  if matched_coords.empty:  return pd.concat([children, parents], ignore_index=True)   matched = pd.DataFrame()  for coord in matched_coords:  best_pos = best.loc[coord, 'pos']  match_rows = parents[(parents['coord'] == coord) & (parents['pos'] == best_pos)]  matched = pd.concat([matched, match_rows], ignore_index=True)   if matched.empty:  return pd.concat([children, parents], ignore_index=True)   # --- 3. Compute Poisson p-value per parent ---  summed = matched.groupby('sample')[['called_median', 'bg_median']].sum()  # Ensure k=observed, mu=expected  pvals = ss.poisson.cdf(summed['called_median'], summed['bg_median'])  summed['log_pval'] = np.abs(np.log10(np.clip(pvals, 1e-300, 1.0))) # avoid log(0)   # Add metadata  summed['coord'] = 'sum'  summed['pos'] = 0  if not matched.empty:  summed['name'] = matched.iloc[0]['name']  summed.reset_index(inplace=True)   # --- 4. Combine children, parents, and parent summary ---  final_parents = pd.concat([matched, summed], ignore_index=True)  final_stats = pd.concat([children, final_parents], ignore_index=True)   return final_stats | assert choose_parent_counts(pd.DataFrame({  'sample': ['fa', 'mo'],  'coord': ['posA', 'posB'],  'pos': [100, 200],  'log_pval': [1.0, 2.0],  'called_median': [10, 5],  'bg_median': [5, 3],  'name': ['cnv1', 'cnv1'] })) == "sample coord pos log_pval called_median bg_median name 0 fa posA 100 1.0 10 5 cnv1 1 mo posB 200 2.0 5 3 cnv1" assert choose_parent_counts(pd.DataFrame({  'sample': ['p1', 's1'],  'coord': ['posA', 'posB'],  'pos': [100, 200],  'log_pval': [3.0, 4.0],  'called_median': [10, 5],  'bg_median': [5, 3],  'name': ['cnv1', 'cnv1'] })) == " sample coord pos log_pval called_median bg_median name 0 p1 posA 100 3.0 10 5 cnv1 1 s1 posB 200 4.0 5 3 cnv1" assert choose_parent_counts(pd.DataFrame({  'sample': ['p1', 's1', 'fa', 'mo'],  'coord': ['posA', 'posB', 'posA', 'posB'],  'pos': [100, 200, 300, 400], # parent pos 与 best pos 不匹配  'log_pval': [3.0, 4.0, 1.0, 2.0],  'called_median': [10, 5, 5, 5],  'bg_median': [5, 3, 2, 1],  'name': ['cnv1', 'cnv1', 'cnv1', 'cnv1'] })) == "sample coord pos log_pval called_median bg_median name 0 p1 posA 100 3.0 10 5 cnv1 1 s1 posB 200 4.0 5 3 cnv1 2 fa posA 300 1.0 5 2 cnv1 3 mo posB 400 2.0 5 1 cnv1" assert choose_parent_counts(pd.DataFrame({  'sample': ['p1', 's1', 'fa', 'mo'],  'coord': ['posA', 'posB', 'posA', 'posB'],  'pos': [100, 200, 100, 200],  'log_pval': [10, 20, 5, 15],  'called_median': [1000, 2000, 500, 1500],  'bg_median': [1, 1, 1, 1],  'name': ['cnv1', 'cnv1', 'cnv1', 'cnv1'] })) == "sample coord pos log_pval called_median bg_median name 0 p1 posA 100 10.0 1000 1 cnv1 1 s1 posB 200 20.0 2000 1 cnv1 2 fa posA 100 5.0 500 1 cnv1 3 mo posB 200 15.0 1500 1 cnv1 4 fa sum 0 0.0 500 1 cnv1 5 mo sum 0 0.0 1500 1 cnv1" | choose_parent_counts(pd.DataFrame({  'sample': ['fa', 'mo'],  'coord': ['posA', 'posB'],  'pos': [100, 200],  'log_pval': [1.0, 2.0],  'called_median': [10, 5],  'bg_median': [5, 3],  'name': ['cnv1', 'cnv1'] })) raises AttributeError choose_parent_counts(pd.DataFrame({  'sample': ['p1', 's1'],  'coord': ['posA', 'posB'],  'pos': [100, 200],  'log_pval': [3.0, 4.0],  'called_median': [10, 5],  'bg_median': [5, 3],  'name': ['cnv1', 'cnv1'] })) raises IndexError choose_parent_counts(pd.DataFrame({  'sample': ['p1', 's1', 'fa', 'mo'],  'coord': ['posA', 'posB', 'posA', 'posB'],  'pos': [100, 200, 300, 400], # parent pos 与 best pos 不匹配  'log_pval': [3.0, 4.0, 1.0, 2.0],  'called_median': [10, 5, 5, 5],  'bg_median': [5, 3, 2, 1],  'name': ['cnv1', 'cnv1', 'cnv1', 'cnv1'] })) raises IndexError choose_parent_counts(pd.DataFrame({  'sample': ['p1', 's1', 'fa', 'mo'],  'coord': ['posA', 'posB', 'posA', 'posB'],  'pos': [100, 200, 100, 200],  'log_pval': [10, 20, 5, 15],  'called_median': [1000, 2000, 500, 1500],  'bg_median': [1, 1, 1, 1],  'name': ['cnv1', 'cnv1', 'cnv1', 'cnv1'] })) = "sample coord pos log_pval called_median bg_median name 0 p1 posA 100 10.000000 1000 1 cnv1 1 s1 posB 200 20.000000 2000 1 cnv1 2 fa posA 100 5.000000 500 1 cnv1 3 mo posB 200 15.000000 1500 1 cnv1 0 fa sum 0 214.447403 500 1 cnv1 1 mo sum 0 inf 1500 1 cnv1" | The original version can fail or produce incorrect results when children or parent entries are missing, when best positions don’t match any parent, or when Poisson p-values are extremely small. The optimized version handles these edge cases safely, avoids errors, and ensures consistent and reliable `log_pval` calculations. | medium | alternative implementation |
| gatk-sv-main(1076) | def identify_mosaics(metrics):  metrics = metrics.loc[metrics.Median_Separation.astype(  str) != 'coverage_failure'].copy()  metrics.Median_Separation = metrics.Median_Separation.astype(float)   q1 = metrics.Median_Separation.quantile(0.25)  q3 = metrics.Median_Separation.quantile(0.75)  IQR = q3 - q1   cutoff = q1 - (1.5 * IQR)   mosaics = metrics.loc[metrics.Median_Separation < cutoff]   return mosaics['CNVID SampleIDs Median_Separation'.split()] | def identify_mosaics(metrics):  metrics_clean = metrics.copy()  metrics_clean['Median_Separation'] = pd.to_numeric(metrics_clean['Median_Separation'], errors='coerce')  metrics_clean = metrics_clean.dropna(subset=['Median_Separation'])  q1 = metrics_clean['Median_Separation'].quantile(0.25)  q3 = metrics_clean['Median_Separation'].quantile(0.75)  IQR = q3 - q1  cutoff = q1 - 1.5 * IQR  mosaics = metrics_clean[metrics_clean['Median_Separation'] < cutoff]  return mosaics[['CNVID','SampleIDs','Median_Separation']] | assert identify_mosaics_original({'CNVID': 'cnv5', 'SampleIDs': 's5', 'Median_Separation': 'abc'}) == "Empty DataFrame Columns: [CNVID, SampleIDs, Median_Separation] Index: []" | identify_mosaics_original({'CNVID': 'cnv5', 'SampleIDs': 's5', 'Median_Separation': 'abc'}) raises ValueError | The original version fails or crashes when `Median_Separation` contains non-numeric or missing values, whereas the optimized version safely handles such cases by converting invalid entries to NaN and dropping them. This makes the optimized version more robust and reliable for real-world datasets. | low |  |
| gatk-sv-main(1076) | def samples_overlap(samplesA, samplesB, upper_thresh=0.8, lower_thresh=0.5):  # Get lists of called samples for each record  samplesA = set(samplesA)  samplesB = set(samplesB)   # Compute fraction of each record's samples which are shared  shared = samplesA & samplesB  fracA = len(shared) / len(samplesA)  fracB = len(shared) / len(samplesB)   min_frac, max_frac = sorted([fracA, fracB])   return min_frac >= lower_thresh and max_frac >= upper_thresh | def samples_overlap(samplesA, samplesB, upper_thresh=0.8, lower_thresh=0.5):  """  Determine if two sets of samples overlap sufficiently.    Args:  samplesA (iterable): First list or set of samples.  samplesB (iterable): Second list or set of samples.  upper_thresh (float): Minimum fraction of the larger set that must overlap.  lower_thresh (float): Minimum fraction of the smaller set that must overlap.    Returns:  bool: True if overlap meets thresholds, False otherwise.  """  # Convert to sets  samplesA = set(samplesA)  samplesB = set(samplesB)   # Return False immediately if any set is empty  if not samplesA or not samplesB:  return False   # Compute shared samples  shared = samplesA & samplesB  fracA = len(shared) / len(samplesA)  fracB = len(shared) / len(samplesB)   # Identify smaller/larger fraction  min_frac, max_frac = sorted([fracA, fracB])   return min_frac >= lower_thresh and max_frac >= upper_thresh | assert samples_overlap([], []) == False assert samples_overlap(["s1", "s2"], []) == False | samples_overlap([], []) raises ZeroDivisionError samples_overlap(["s1", "s2"], []) raises ZeroDivisionError | The optimized version is more robust: it safely handles empty or small sample sets, avoids division-by-zero errors, and strictly enforces the overlap thresholds, ensuring consistent and reliable results. | low |  |
| gatk-sv-main(1076) | def reformat_dn_rdtest(dn_metrics):  id_vars = 'chr Start End CNVID Type Family AffectedMember'.split()  metrics = pd.melt(dn_metrics, id_vars=id_vars)   metrics['metric'] = metrics.variable.str.split('.').str[1]  metrics['member'] = metrics.variable.str.split('.').str[0].str.lower()  metrics.member = metrics.member.replace({'pro': 'p1', 'sib': 's1'})   # restrict to parents  # metrics = metrics.loc[(metrics.member != 'pro') &  # (metrics.member != 'sib')].copy()   # Restrict to parents of candidate de novo variants  metrics = metrics.loc[(~metrics.AffectedMember.str.contains('fa')) &  (~metrics.AffectedMember.str.contains('mo'))].copy()   metrics['SampleIDs'] = (metrics['Family'].astype(str) +  '.' +  metrics['member'])   # Restrict to ID and metric cols  cols = 'chr Start End CNVID SampleIDs Type metric value'.split()  metrics = metrics[cols].copy()   # Pivot out to standard RdTest format  id_vars = 'chr Start End CNVID SampleIDs Type'.split()  metrics = metrics.pivot_table(values='value',  index=id_vars,  columns='metric').reset_index()   # Rename and add standard RdTest columns  repl = dict(secMaxP='2ndMaxP', rank='Median_Rank', Sep='Median_Separation')  metrics = metrics.rename(columns=repl)  metrics['Median_Power'] = np.nan  metrics['Model'] = 'denovo'   # Re-order and return  cols = ('chr Start End CNVID SampleIDs Type Median_Power P 2ndMaxP Model '  'Median_Rank Median_Separation').split()   return metrics[cols].copy() | def reformat_dn_rdtest(dn_metrics: pd.DataFrame):  # Required columns  required_cols = set("chr Start End CNVID Type Family AffectedMember".split())  if not required_cols.issubset(dn_metrics.columns):  missing = required_cols - set(dn_metrics.columns)  raise ValueError(f"Missing required columns: {missing}")   # Identify metric variable columns automatically  variable_cols = [c for c in dn_metrics.columns if "." in c and c not in required_cols]   if not variable_cols:  raise ValueError("No metric columns found (columns containing '.')")   # Melt to long format  metrics = pd.melt(  dn_metrics,  id_vars=list(required_cols),  value_vars=variable_cols,  var_name="variable",  value_name="value"  )   # Parse 'metric' and 'member'  split_vals = metrics["variable"].str.split(".", n=1, expand=True)  metrics["member"] = split_vals[0].str.lower()  metrics["metric"] = split_vals[1]   # Normalize family member names  metrics["member"] = metrics["member"].replace({"pro": "p1", "sib": "s1"})   # Restrict to parents logically:  # Only keep rows where AffectedMember is child (not fa/mo)  metrics = metrics.loc[  ~metrics["AffectedMember"].str.endswith(("fa", "mo"))  ].copy()   # Construct SampleIDs  metrics["SampleIDs"] = metrics["Family"].astype(str) + "." + metrics["member"]   # Final columns selection  metrics = metrics[  ["chr", "Start", "End", "CNVID", "SampleIDs", "Type", "metric", "value"]  ].copy()   # Pivot to RdTest format  metrics = metrics.pivot_table(  values="value",  index=["chr", "Start", "End", "CNVID", "SampleIDs", "Type"],  columns="metric",  aggfunc="first" # prevents loss when duplicates appear  ).reset_index()   # Rename  metrics = metrics.rename(columns={  "secMaxP": "2ndMaxP",  "rank": "Median_Rank",  "Sep": "Median_Separation",  })   # Add standard RdTest columns  metrics["Median_Power"] = np.nan  metrics["Model"] = "denovo"   # Ensure all expected RdTest cols exist  expected = (  "chr Start End CNVID SampleIDs Type Median_Power P 2ndMaxP "  "Model Median_Rank Median_Separation"  ).split()   for col in expected:  if col not in metrics.columns:  metrics[col] = np.nan   return metrics[expected].copy() | assert reformat_dn_rdtest(pd.DataFrame([  {  "chr": "1", "Start": 100, "End": 200, "CNVID": "cnv1",  "Type": "DEL", "Family": "F1", "AffectedMember": "p1",  "pro.P": 0.1, "pro.secMaxP": 0.2, "pro.rank": 1  },  {  "chr": "1", "Start": 100, "End": 200, "CNVID": "cnv1",  "Type": "DEL", "Family": "F1", "AffectedMember": "p1",  "sib.P": 0.15, "sib.Sep": 0.3  } ])) == "metric chr Start End CNVID ... 2ndMaxP Model Median_Rank Median_Separation 0 1 100 200 cnv1 ... 0.2 denovo 1.0 NaN 1 1 100 200 cnv1 ... NaN denovo NaN 0.3" assert reformat_dn_rdtest(pd.DataFrame([  {  "chr": "3", "Start": 10, "End": 20, "CNVID": "cnv3",  "Type": "INS", "Family": "F3", "AffectedMember": "p1",  "pro.P": 0.8  },  {  "chr": "3", "Start": 10, "End": 20, "CNVID": "cnv3",  "Type": "INS", "Family": "F3", "AffectedMember": "mo",  "pro.P": 0.9 # should be filtered out  } ])) == "metric chr Start End CNVID ... 2ndMaxP Model Median_Rank Median_Separation 0 2 300 500 cnv2 ... NaN denovo NaN NaN" assert reformat_dn_rdtest(pd.DataFrame([  {  "chr": "3", "Start": 10, "End": 20, "CNVID": "cnv3",  "Type": "INS", "Family": "F3", "AffectedMember": "p1",  "pro.P": 0.8  },  {  "chr": "3", "Start": 10, "End": 20, "CNVID": "cnv3",  "Type": "INS", "Family": "F3", "AffectedMember": "mo",  "pro.P": 0.9 # should be filtered out  } ])) == "metric chr Start End CNVID ... 2ndMaxP Model Median_Rank Median_Separation 0 3 10 20 cnv3 ... NaN denovo NaN NaN" assert reformat_dn_rdtest(pd.DataFrame([  {  "chr": "4", "Start": 40, "End": 60, "CNVID": "cnv4",  "Type": "DEL", "Family": "F4", "AffectedMember": "c1",  "pro.P": 0.1  },  {  "chr": "4", "Start": 40, "End": 60, "CNVID": "cnv4",  "Type": "DEL", "Family": "F4", "AffectedMember": "c1",  "pro.P": 0.12 # duplicate → original code will break  } ])) == "metric chr Start End CNVID ... 2ndMaxP Model Median_Rank Median_Separation 0 4 40 60 cnv4 ... NaN denovo NaN NaN" assert reformat_dn_rdtest(pd.DataFrame([  {  "chr": "5", "Start": 400, "End": 800, "CNVID": "cnv5",  "Type": "DUP", "Family": "F5", "AffectedMember": "p1",  "pro.rank": 2  } ])) == "metric chr Start End CNVID ... 2ndMaxP Model Median_Rank Median_Separation 0 5 400 800 cnv5 ... NaN denovo 2 NaN" | reformat_dn_rdtest(pd.DataFrame([  {  "chr": "3", "Start": 10, "End": 20, "CNVID": "cnv3",  "Type": "INS", "Family": "F3", "AffectedMember": "p1",  "pro.P": 0.8  },  {  "chr": "3", "Start": 10, "End": 20, "CNVID": "cnv3",  "Type": "INS", "Family": "F3", "AffectedMember": "mo",  "pro.P": 0.9 # should be filtered out  } ])) raises KeyError (same for test case 2-5) | The function contains several functional bugs. Most critically, the filtering step intended to keep parental samples actually removes them, because rows containing "fa" or "mo" in AffectedMember are excluded. This behavior contradicts the comment (“Restrict to parents”) and results in silently losing all parental data. Additional issues include unsafe parsing of variable names, ambiguous substring matching (“fa”), and fragile pivoting logic that fails when metrics are missing. | low |  |
| gatk-sv-main(1076) | def recalculate_gq(sl, scale_factor, is_hom_ref, upper, lower, shift):  sign = -1 if is_hom_ref else 1  sl = (sign * min(max(sl, lower), upper)) + shift  return int(math.floor(-10 * math.log10(1 / (math.pow(scale_factor, sl) + 1)))) | def recalculate_gq(sl, scale_factor, is_hom_ref, upper, lower, shift, max_gq=99):   # --- Validate inputs ---  if scale_factor is None or not isinstance(scale_factor, (int, float)):  raise ValueError("scale_factor must be a numeric value.")  if scale_factor <= 0:  raise ValueError("scale_factor must be > 0.")  if lower > upper:  raise ValueError("lower cannot exceed upper.")   # --- Step 1: Clamp sl ---  sl_clipped = min(max(sl, lower), upper)   # --- Step 2: Apply sign based on genotype ---  sign = -1 if is_hom_ref else 1  sl_adj = sign * sl_clipped + shift   # --- Step 3: Compute exponent safely ---  # Avoid pow overflow by clamping exponent  # If scale_factor > 1, exponent grows quickly  MAX_EXPONENT = 300 # ~ 1e300 magnitude limit for double precision   # Convert exponent to natural log space: pow(a, b) = exp(b * ln(a))  try:  exponent_value = sl_adj * math.log(scale_factor)  except ValueError:  # In case scale_factor is invalid; we already check but double safety  exponent_value = -999   if exponent_value > MAX_EXPONENT:  # saturate to avoid overflow → GQ will approach max  gq = max_gq  return gq   if exponent_value < -MAX_EXPONENT:  # extremely small → scale_factor^sl ≈ 0  pow_val = 0.0  else:  pow_val = math.exp(exponent_value)   # --- Step 4: Compute GQ ---  # Original formula equivalent: GQ = 10 * log10(1 + a^b)  raw_gq = 10 * math.log10(1 + pow_val)   # --- Step 5: Ensure valid GQ range ---  if raw_gq < 0:  raw_gq = 0  if raw_gq > max_gq:  raw_gq = max_gq   return int(math.floor(raw_gq)) | assert recalculate_gq(sl=200, scale_factor=10, is_hom_ref=False, upper=500, lower=-500, shift=0) == 99 assert recalculate_gq(sl=5, scale_factor=3, is_hom_ref=False, upper=10, lower=-10, shift=50) == 99 assert recalculate_gq(sl=2, scale_factor=1e10, is_hom_ref=False, upper=10, lower=-10, shift=0) == 99 | recalculate_gq(sl=200, scale_factor=10, is_hom_ref=False, upper=500, lower=-500, shift=0) = 2000 recalculate_gq(sl=5, scale_factor=3, is_hom_ref=False, upper=10, lower=-10, shift=50) == 262 recalculate_gq(sl=2, scale_factor=1e10, is_hom_ref=False, upper=10, lower=-10, shift=0) == 200 | Improvements over the original implementation:  Added strict parameter validation (e.g., scale_factor > 0, numeric inputs).  Prevented floating-point overflow by clamping the exponent in log-space.  Ensured numerical stability for extremely large or small SL values.  Guaranteed that GQ is always within the valid VCF range (0–99 by default).  Avoided negative or undefined values resulting from invalid log10 calls.  Simplified the mathematical expression for readability while preserving semantics.  Increased robustness for real data where SL and scale factors vary widely. | medium | domain knowledge violation |
| gatk-sv-main(1076) | def rmsstd(intervals):  starts = np.array([interval.start for interval in intervals])  ends = np.array([interval.end for interval in intervals])   def _meanSS(X):  mu = np.mean(X)  return np.sum((X - mu) ** 2) / len(X)   SS = _meanSS(starts) + _meanSS(ends)  return np.sqrt(SS) | import numpy as np import math  def rmsstd(intervals, *, use_sample_variance=True, ignore_invalid=True, return_nan_if_empty=False):  """  Compute RMSSTD (root mean sum of squared deviations) for interval start/end positions.   Parameters:  ----------  intervals : list of objects (each must have .start and .end)  use_sample_variance : bool  True → use unbiased sample variance (divide by n-1)  False → use population variance (divide by n)  ignore_invalid : bool  True → skip intervals with invalid or reversed coordinates  False → raise ValueError on invalid interval  return_nan_if_empty : bool  True → return np.nan if no valid intervals  False → return 0   Returns:  --------  float : RMSSTD value  """   # Extract valid intervals  clean_starts = []  clean_ends = []    for iv in intervals:  try:  s = float(iv.start)  e = float(iv.end)  except Exception:  if ignore_invalid:  continue  else:  raise ValueError(f"Invalid interval object: {iv}")   # If reversed interval, fix or ignore  if s > e:  if ignore_invalid:  s, e = e, s # auto-fix reversed intervals  else:  raise ValueError(f"Reversed interval ({s}, {e})")   if math.isnan(s) or math.isnan(e):  if ignore_invalid:  continue  else:  raise ValueError("Encountered NaN interval coordinate")   clean_starts.append(s)  clean_ends.append(e)   n = len(clean_starts)   # No valid intervals  if n == 0:  return np.nan if return_nan_if_empty else 0.0   # Single interval → no spread  if n == 1:  return 0.0   # Convert to arrays  starts = np.array(clean_starts)  ends = np.array(clean_ends)   # Internal function  def variance(X):  mu = X.mean()  if use_sample_variance and len(X) > 1:  return ((X - mu)**2).sum() / (len(X) - 1)  else:  return ((X - mu)**2).sum() / len(X)   SS = variance(starts) + variance(ends)  return float(np.sqrt(SS)) | assert rmsstd([IV(10,20), IV(12,22), IV(14,24)] == 2.8284271247461903 assert rmsstd([IV(10,20)]) == 0.0 assert rmsstd([]) == 0.0 assert rmsstd([IV(10,20), IV(float('nan'), 50)]) == 0.0 | rmsstd([IV(10,20), IV(12,22), IV(14,24)] = 2.309401076758503 rmsstd([]) = nan rmsstd([IV(10,20), IV(float('nan'), 50)]) = nan | The improved version provides full input validation, supports empty and singleton input sets, corrects reversed intervals, optionally uses unbiased sample variance, and handles NaN/invalid values gracefully. This ensures robust statistical behavior and prevents runtime errors that occur in the original implementation. | medium | alternative implementation |
| gatk-sv-main(1076) | def _hardy_weinberg_chi_squared_to_num_hets_boundary(  hardy_weinberg_confidence: float,  alt_allele_frequency: numpy.ndarray,  num_ref_genotypes: numpy.ndarray,  num_called_genotypes: Union[numpy.ndarray, int] ) -> (numpy.ndarray, numpy.ndarray):  """  Given a likelihood threshold and existing observed allele / genotype data, compute confidence interval on the number  of HET genotypes compatible with Hardy-Weinberg Equilibrium (HWE). Uses chi2 distribution as model of variability.  Args:  hardy_weinberg_confidence: float  Proportion of calls that should be in confidence interval for number of HET calls, assuming HWE  alt_allele_frequency: numpy.ndarray  num_variants length array of allele frequency for ALT allele  num_ref_genotypes: numpy.ndarray  number of genotypes called REF for each variant  num_called_genotypes: numpy.ndarray or int  number of genotypes called for each variant  Returns:  num_expected_het: numpy.ndarray  number of expected HET calls, if each variant was in HWE  het_delta: numpy.ndarray  +-delta to high/low boundary of confidence interval for HET calls in HWE  """  # get chi-squared threshold at desired rareness, with one degree of freedom (only #HETs varying)  chi_squared = scipy.stats.chi2.ppf(hardy_weinberg_confidence, df=1)  ref_allele_frequency = 1.0 - alt_allele_frequency  num_expected_ref = num_called_genotypes * ref_allele_frequency ** 2  num_expected_het = num_called_genotypes * 2 * ref_allele_frequency * alt_allele_frequency  # MATH and logic below  # num_expected_homvar = num_called_genotypes * alt_allele_frequency ** 2  # chi_squared = (  # (num_ref_genotypes - num_expected_ref) ** 2 +  # (num_homvar_genotypes - num_expected_homvar) ** 2 +  # (num_het_genotypes - num_expected_het) ** 2  # ) / num_called_genotypes  # num_homvar_genotypes = (num_alt_alleles - num_het_genotypes) / 2  # num_homvar_genotypes = num_called_genotypes * alt_allele_frequency - num_het_genotypes / 2  # num_homvar_genotypes = num_expected_homvar / alt_allele_frequency - num_het_genotypes / 2  # => num_homvar_genotypes - num_expected_homvar =  # = num_expected_homvar * (1 - alt_allele_frequency) / alt_allele_frequency - num_het_genotypes / 2  # = num_expected_homvar * ref_allele_frequency / alt_allele_frequency - num_het_genotypes / 2  # = num_expected_het / 2 - num_het_genotypes / 2  # chi_squared = (  # (num_ref_genotypes - num_expected_ref) ** 2 +  # 1/4 * (num_het_genotypes - num_expected_het) ** 2 +  # (num_het_genotypes - num_expected_het) ** 2  # ) / num_called_genotypes  # het_delta = +/- abs(num_het_genotypes - num_expected_het)  # = sqrt(4/5 * (chi_squared * num_called_genotypes - (num_ref_genotypes - num_expected_ref) ** 2)  het_delta = numpy.sqrt(  numpy.maximum(  0.8 * (chi_squared * num_called_genotypes - (num_ref_genotypes - num_expected_ref) ** 2),  0  )  )  return num_expected_het, het_delta | import numpy as np import scipy.stats   def _hardy_weinberg_chi_squared_to_num_hets_boundary(  hardy_weinberg_confidence: float,  alt_allele_frequency: np.ndarray,  num_ref_genotypes: np.ndarray,  num_called_genotypes ):  """  Improved and mathematically consistent estimation of expected HET count  and its chi-squared confidence interval boundary under HWE.  """   num_called_genotypes = np.asarray(num_called_genotypes)   # χ² threshold for 1 degree of freedom  chi_squared_threshold = scipy.stats.chi2.ppf(hardy_weinberg_confidence, df=1)   p = alt_allele_frequency  q = 1 - p  N = num_called_genotypes   # Expected counts under HWE  expected_ref = N * (q ** 2)  expected_het = N * (2 * p * q)  expected_homvar = N * (p ** 2)   # Variance of HET count (from multinomial HWE model)  het_var = N * (2 * p * q) * (1 - 2 * p * q)   # Avoid variance = 0 causing nan  het_var = np.maximum(het_var, 1e-12)   # Compute how much (num_ref - expected_ref) contributes to χ²  ref_component = (num_ref_genotypes - expected_ref) ** 2   # Remaining χ² budget for HET deviation  remain = chi_squared_threshold * N - ref_component   # Negative means no deviation allowed  remain = np.maximum(remain, 0)   # Δ is the maximal allowed deviation for HET count  het_delta = np.sqrt(remain * (expected_het / (expected_ref + expected_homvar + 1e-12)))   return expected_het, het_delta | assert _hardy_weinberg_chi_squared_to_num_hets_boundary(0.95, np.array([0.1, 0.25, 0.5]), np.array([90, 130, 80]), np.array([200, 200, 200])) == (array([ 36., 75., 100.]), array([ 0. , 19.22585268, 0. ])) assert _hardy_weinberg_chi_squared_to_num_hets_boundary(0.99, np.array([0.01]), np.array([495]),np.array([500])) == (array([9.9]), array([51.32598407])) assert _hardy_weinberg_chi_squared_to_num_hets_boundary(0.95, np.array([0.3]), np.array([350]),1000) == (array([420.]), array([0.])) | _hardy_weinberg_chi_squared_to_num_hets_boundary(0.95, np.array([0.1, 0.25, 0.5]), np.array([90, 130, 80]), np.array([200, 200, 200])) = (array([ 36., 75., 100.]), array([ 0. , 16.65007683, 0. ])) _hardy_weinberg_chi_squared_to_num_hets_boundary(0.99, np.array([0.01]), np.array([495]),np.array([500])) == (array([9.9]), array([8.15581822])) | The original implementation used an incomplete and mathematically inconsistent derivation of the chi-squared contribution for HET counts under Hardy–Weinberg Equilibrium (HWE). It relied on a fixed heuristic (0.8 * (...)) that did not correspond to any formal statistical model, and could produce negative values inside the square root, unstable behavior when allele frequency approached 0 or 1, and incorrect confidence intervals when the REF deviation dominated the χ² budget.  The improved version provides a fully consistent, variance-based formulation derived from the multinomial HWE model. It correctly computes expected REF, HET, and HOMVAR counts; uses the proper chi-squared threshold with 1 degree of freedom; incorporates the contribution of REF deviation explicitly; ensures non-negative remaining χ² budget; avoids variance-zero issues; and supports both scalar and vectorized inputs. This results in mathematically valid, numerically stable, and biologically interpretable confidence intervals for HET counts. | medium | domain knowledge violation |
| gubbins-master(89) | def chunks(l, k):  n = len(l)  return [l[i * (n // k) + min(i, n % k):(i+1) * (n // k) + min(i+1, n % k)] for i in range(k)] | from typing import List, Any  def chunks(l: List[Any], k: int) -> List[List[Any]]:  """  Split a list `l` into `k` approximately equal chunks.    Parameters  ----------  l : List[Any]  Input list to split  k : int  Number of chunks to create    Returns  -------  List[List[Any]]  List of length `k` containing sublists of `l`. Empty lists are returned if k > len(l).    Notes  -----  - The first (len(l) % k) chunks will have one more element than the others.  - If k <= 0, raises ValueError.  """  if k <= 0:  raise ValueError("k must be a positive integer")    n = len(l)  q, r = divmod(n, k) # q = base size, r = remainder    chunks_list = []  start = 0  for i in range(k):  end = start + q + (1 if i < r else 0)  chunks_list.append(l[start:end])  start = end  return chunks_list | assert chunks([1,2,3,4,5,6,7], 3) == [[1, 2, 3], [4, 5], [6, 7]] assert chunks([1,2,3,4], 2) == [[1, 2], [3, 4]] assert chunks([1,2], 5) == [[1], [2], [], [], []] assert chunks([], 3) == [[], [], []] chunks([1,2,3], 0) raises ValueError("k must be a positive integer") | chunks([1,2,3], 0) = [] | The original version used a compact one-liner with integer division and `min` to split the list, but it lacked checks for invalid `k` values and could behave unexpectedly when `k > len(l)` or `k <= 0`.  The improved version is more robust: it explicitly computes the base chunk size and remainder, handles edge cases (`k <= 0`, `k > len(l)`), ensures exactly `k` chunks are returned (empty if necessary), and is easier to read and maintain. | low |  |
| modlAMP(39) | def count_ngrams(seq, n):  """Function to count the n-grams of an amino acid sequence. N can be one integer or a list of integers   :param seq: {str} amino acid sequence  :param n: {int or list of ints} defines whether counts or frequencies are given for each AA  :return: {dict} dictionary with n-grams as keys and their counts in the sequence as values.  """  if seq == "":  seq = " "  if isinstance(n, int):  n = [n]  ngrams = list()  for i in n:  ngrams.extend([seq[j : j + i] for j in range(len(seq) - (i - 1))])  counts = {g: (seq.count(g)) for g in set(ngrams)}  counts = collections.OrderedDict(sorted(counts.items(), key=operator.itemgetter(1), reverse=True))  return counts | import collections from typing import Union, List, Dict  def count_ngrams(seq: str, n: Union[int, List[int]]) -> Dict[str, int]:  """  Count n-grams in an amino acid sequence.    Parameters  ----------  seq : str  Amino acid sequence  n : int or list of ints  Length(s) of n-grams to count    Returns  -------  Dict[str, int]  Ordered dictionary of n-grams and their counts (descending order)  """  if not seq:  return {}   seq = seq.upper() # Make sequence case-insensitive   if isinstance(n, int):  n = [n]   # Filter out invalid n values  n = [i for i in n if i > 0 and i <= len(seq)]  if not n:  return {}   all_ngrams = []  for size in n:  all_ngrams.extend([seq[i:i+size] for i in range(len(seq)-size+1)])   counts = collections.Counter(all_ngrams)  # Sort by count descending, then by n-gram alphabetically  counts = collections.OrderedDict(sorted(counts.items(), key=lambda x: (-x[1], x[0])))   return counts | assert count_ngrams("ACacGAC", 1) == OrderedDict([('A', 3), ('C', 3), ('G', 1)]) assert count_ngrams("ACacGAC", 2) == OrderedDict([('AC', 3), ('CA', 1), ('CG', 1), ('GA', 1)]) assert count_ngrams("ACacGAC",[1, 2]) == OrderedDict([('A', 3), ('AC', 3), ('C', 3), ('CA', 1), ('CG', 1), ('G', 1), ('GA', 1)]) | count_ngrams("ACacGAC", 1) = OrderedDict([('C', 2), ('A', 2), ('G', 1), ('c', 1), ('a', 1)]) count_ngrams("ACacGAC", 2) = OrderedDict([('AC', 2), ('Ca', 1), ('cG', 1), ('ac', 1), ('GA', 1)]) count_ngrams("ACacGAC",[1, 2]) = OrderedDict([('C', 2), ('A', 2), ('AC', 2), ('Ca', 1), ('G', 1), ('cG', 1), ('ac', 1), ('GA', 1), ('c', 1), ('a', 1)]) | The improved `count_ngrams` version is faster, more robust, and consistent: it uses `collections.Counter` for efficient counting instead of repeatedly calling `seq.count()`, handles empty sequences and invalid n values gracefully, standardizes sequence case for consistent counting, and produces a stably sorted output by count and then alphabetically, while still supporting both single integers and lists for n-gram sizes. | low |  |
| modlAMP(39) | def _one_crosscorr(seq, window, scale):  """Private function used for calculating cross-correlated descriptors for 1 given sequence, window and an AA scale.  This function is used by the :py:func:`calculate_crosscorr` method of :py:class:`PeptideDescriptor`.   :param seq: {str} amino acid sequence to calculate descriptor for  :param window: {int} correlation-window size  :param scale: {str} amino acid scale to be used to calculate descriptor  :return: {numpy.array} calculated descriptor data  """  try:  m = list() # list of lists to store translated sequence values  for shp in range(len(seq)): # translate AA sequence into values  m.append(scale[str(seq[shp])])  # auto-correlation in defined sequence window  seqdesc = list()  for val in range(len(scale["A"])): # for all features of the descriptor scale  for cc in range(len(scale["A"])): # for every feature cross correlation  if (val + cc) < len(scale["A"]): # check if corr distance is in range of the num of features  for dist in range(window): # for all correlation distances  cntr = float()  valsum = list()  for pos in range(len(seq)): # for every position in the sequence  if (pos + dist) < len(seq): # check if corr distance is possible at that sequence pos  cntr += 1 # counter to scale sum  valsum.append(m[pos][val] * m[pos + dist][val + cc])  seqdesc.append(sum(valsum) / cntr) # append scaled correlation distance values  return seqdesc  except ZeroDivisionError:  print("ERROR!\nThe chosen correlation window % i is larger than the sequence %s !" % (window, seq)) | import numpy as np  def _one_crosscorr(seq: str, window: int, scale: dict) -> np.ndarray:  """  Compute cross-correlated descriptors for a given amino acid sequence, window, and AA scale.  Optimized, vectorized and robust version.   Parameters  ----------  seq : str  Amino acid sequence  window : int  Correlation window size  scale : dict  Dictionary mapping AA letters to numeric feature vectors   Returns  -------  np.ndarray  1D array of cross-correlation values  """  # Input validation  if not seq:  raise ValueError("Sequence must be non-empty.")  if window < 1 or window > len(seq):  raise ValueError(f"Window size {window} is invalid for sequence length {len(seq)}.")   # Translate sequence to numeric matrix (num_positions x num_features)  try:  m = np.array([scale[aa] for aa in seq], dtype=np.float32)  except KeyError as e:  raise KeyError(f"Amino acid '{e.args[0]}' not found in scale dictionary.") from None   num_pos, num_feat = m.shape  result = []   # Vectorized cross-correlation  for val in range(num_feat):  for cc in range(num_feat):  # Skip out-of-range feature pairs  if val + cc >= num_feat:  continue  for dist in range(window):  if num_pos - dist <= 0:  continue  prod = m[:num_pos - dist, val] * m[dist:, val + cc]  corr_value = np.mean(prod)  result.append(corr_value)   return np.array(result, dtype=np.float32) | assert _one_crosscorr("ACDE", 2, simple_scale = {  "A": [1.0, 0.5, 0.2],  "C": [0.8, 0.3, 0.1],  "D": [0.5, 0.9, 0.4],  "E": [0.6, 0.7, 0.8],  "F": [0.2, 0.4, 0.6], }) == [0.5625, 0.5, 0.40250003, 0.45666668, 0.24000001, 0.27333334, 0.41, 0.35, 0.2625, 0.29666665, 0.2125, 0.12666668] assert _one_crosscorr("ACDE", 4, simple_scale = {  "A": [1.0, 0.5, 0.2],  "C": [0.8, 0.3, 0.1],  "D": [0.5, 0.9, 0.4],  "E": [0.6, 0.7, 0.8],  "F": [0.2, 0.4, 0.6], }) == [0.5625, 0.5, 0.49, 0.6, 0.40250003, 0.45666668, 0.73, 0.7, 0.24000001, 0.27333334, 0.52000004, 0.8, 0.41, 0.35, 0.32999998, 0.35, 0.2625, 0.29666665, 0.22, 0.4, 0.2125, 0.12666668, 0.080000006, 0.16000001] assert _one_crosscorr("ACD", 5, simple_scale = {  "A": [1.0, 0.5, 0.2],  "C": [0.8, 0.3, 0.1],  "D": [0.5, 0.9, 0.4],  "E": [0.6, 0.7, 0.8],  "F": [0.2, 0.4, 0.6], }) raises ValueError | _one_crosscorr("ACDE", 2, simple_scale = {  "A": [1.0, 0.5, 0.2],  "C": [0.8, 0.3, 0.1],  "D": [0.5, 0.9, 0.4],  "E": [0.6, 0.7, 0.8],  "F": [0.2, 0.4, 0.6], }) = [0.5625, 0.5000000000000001, 0.40249999999999997, 0.4566666666666667, 0.24, 0.2733333333333334, 0.41, 0.35000000000000003, 0.2625, 0.2966666666666667, 0.21250000000000005, 0.12666666666666668] _one_crosscorr("ACDE", 4, simple_scale = {  "A": [1.0, 0.5, 0.2],  "C": [0.8, 0.3, 0.1],  "D": [0.5, 0.9, 0.4],  "E": [0.6, 0.7, 0.8],  "F": [0.2, 0.4, 0.6], }) = [0.5625, 0.5000000000000001, 0.49, 0.6, 0.40249999999999997, 0.4566666666666667, 0.73, 0.7, 0.24, 0.2733333333333334, 0.52, 0.8, 0.41, 0.35000000000000003, 0.33, 0.35, 0.2625, 0.2966666666666667, 0.22, 0.4, 0.21250000000000005, 0.12666666666666668, 0.08000000000000002, 0.16000000000000003] _one_crosscorr("ACD", 5, simple_scale = {  "A": [1.0, 0.5, 0.2],  "C": [0.8, 0.3, 0.1],  "D": [0.5, 0.9, 0.4],  "E": [0.6, 0.7, 0.8],  "F": [0.2, 0.4, 0.6], }) == None | The optimized version strengthens validation, improves error handling, and makes the computation clearer and more robust. It adds explicit checks for empty sequences, window size constraints, and unknown amino acids, replacing silent failures with clear exceptions. It also simplifies loops, avoids unnecessary list constructions, and enhances readability and maintainability. Overall, the optimized version is safer, more efficient, and more reliable than the original implementation. | low |  |
| modlAMP(39) | def _one_arc(seq, modality, scale):  """Privat function used for calculating arc descriptors for one sequence and AA scale. This function is used by  :py:func:`calculate_arc` method method of :py:class:`PeptideDescriptor`.   :param seq: {str} amino acid sequence to calculate descriptor for  :param scale: {str} amino acid scale to be used to calculate descriptor  :return: {numpy.array} calculated descriptor data  """  desc_mat = []  for aa in seq:  desc_mat.append(scale[aa])  desc_mat = np.asarray(desc_mat)   # Check descriptor dimension  desc_dim = desc_mat.shape[1]   # list to store descriptor values for all windows  allwindows_arc = []   if len(seq) > 18:  window = 18  # calculates number of windows in sequence  num_windows = len(seq) - window  else:  window = len(seq)  num_windows = 1   # loop through all windows  for j in range(num_windows):  # slices descriptor matrix into current window  window_mat = desc_mat[j : j + window, :]   # defines order of amino acids in helical projection  order = [0, 11, 4, 15, 8, 1, 12, 5, 16, 9, 2, 13, 6, 17, 10, 3, 14, 7]   # orders window descriptor matrix into helical projection order  ordered = []  for pos in order:  try:  ordered.append(window_mat[pos, :])  except Exception:  # for sequences of len < 18 adding dummy vector with 2s, length of descriptor dimensions  ordered.append([2] * desc_dim)  ordered = np.asarray(ordered)   window_arc = []   # loop through pharmacophoric features  for m in range(desc_dim):  all_arcs = [] # stores all arcs that can be found of a pharmacophoric feature  arc = 0   for n in range(18): # for all positions in helix, regardless of sequence length  if ordered[n, m] == 0: # if position does not contain pharmacophoric feature  all_arcs.append(arc) # append previous arc to all arcs list  arc = 0 # arc is initialized  elif ordered[n, m] == 1: # if position contains pharmacophoric feature(PF), elongate arc by 20°  arc += 20  elif ordered[n, m] == 2: # if position doesn't contain amino acid:  if ordered[n - 1, m] == 1: # if previous position contained PF add 10°  arc += 10  elif ordered[n - 1, m] == 0: # if previous position didn't contain PF don't add anything  arc += 0  elif ordered[n - 2, m] == 1: # if previous position is empty then check second previous for PF  arc += 10  if n == 17: # if we are at the last position check for position n=0 instead of next position.  if ordered[0, m] == 1: # if it contains PF add 10° extra  arc += 10  else: # if next position contains PF add 10° extra  if ordered[n + 1, m] == 1:  arc += 10  elif ordered[n + 1, m] == 0:  arc += 0  else: # if next position is empty check for 2nd next position  if n == 16:  if ordered[0, m] == 1:  arc += 10  else:  if ordered[n + 2, m] == 1:  arc += 10   all_arcs.append(arc)  if not arc == 360:  arc0 = all_arcs.pop() + all_arcs[0] # join first and last arc together  all_arcs = [arc0] + all_arcs[1:]   window_arc.append(np.max(all_arcs)) # append to window arcs the maximum arc of this PF  allwindows_arc.append(window_arc) # append all PF arcs of this window   allwindows_arc = np.asarray(allwindows_arc)   if modality == "max":  final_arc = np.max(allwindows_arc, axis=0) # calculate maximum / mean arc along all windows  elif modality == "mean":  final_arc = np.mean(allwindows_arc, axis=0)  else:  print('modality is unknown, please choose between "max" and "mean"\n.')  sys.exit()  return final_arc | def _one_arc(seq, modality, scale):  """  Optimized version focusing on fixing the incorrect number of windows.  The rest of the logic is kept fully consistent with the original code.  """   # translate AA sequence into descriptor matrix  desc_mat = []  for aa in seq:  if aa not in scale: # minor robustness improvement  raise KeyError(f"Amino acid '{aa}' not found in scale definition.")  desc_mat.append(scale[aa])  desc_mat = np.asarray(desc_mat)   desc_dim = desc_mat.shape[1] # descriptor dimension   # list to store descriptor values for all windows  allwindows_arc = []   # -------- FIXED WINDOW NUMBER BUG --------  if len(seq) > 18:  window = 18  # original: num_windows = len(seq) - window (WRONG)  num_windows = len(seq) - window + 1 # FIXED  else:  window = len(seq)  num_windows = 1  # -----------------------------------------   # loop through all windows  for j in range(num_windows):   window_mat = desc_mat[j : j + window, :]   order = [0, 11, 4, 15, 8, 1, 12, 5, 16, 9, 2, 13, 6, 17, 10, 3, 14, 7]   # reorder window into helical projection order  ordered = []  for pos in order:  try:  ordered.append(window_mat[pos, :])  except Exception:  ordered.append([2] * desc_dim) # dummy vector  ordered = np.asarray(ordered)   window_arc = []   # loop through descriptor features  for m in range(desc_dim):  all_arcs = []  arc = 0   for n in range(18):   if ordered[n, m] == 0:  all_arcs.append(arc)  arc = 0   elif ordered[n, m] == 1:  arc += 20   elif ordered[n, m] == 2:  if ordered[n - 1, m] == 1:  arc += 10  elif ordered[n - 1, m] == 0:  arc += 0  elif ordered[n - 2, m] == 1:  arc += 10   if n == 17:  if ordered[0, m] == 1:  arc += 10  else:  if ordered[n + 1, m] == 1:  arc += 10  elif ordered[n + 1, m] == 0:  arc += 0  else:  if n == 16:  if ordered[0, m] == 1:  arc += 10  else:  if ordered[n + 2, m] == 1:  arc += 10   all_arcs.append(arc)  if arc != 360:  arc0 = all_arcs.pop() + all_arcs[0]  all_arcs = [arc0] + all_arcs[1:]   window_arc.append(np.max(all_arcs))   allwindows_arc.append(window_arc)   allwindows_arc = np.asarray(allwindows_arc)   if modality == "max":  final_arc = np.max(allwindows_arc, axis=0)  elif modality == "mean":  final_arc = np.mean(allwindows_arc, axis=0)  else:  raise ValueError('modality must be "max" or "mean".')   return final_arc | assert _one_arc("ABCDABCD", modality="max", scale={  "A": [1,0,0],  "B": [0,1,0],  "C": [0,0,1],  "D": [1,1,0], }) == [160 100 100] assert _one_arc("ABCDABCD", modality="mean", scale={  "A": [1,0,0],  "B": [0,1,0],  "C": [0,0,1],  "D": [1,1,0], }) == [160. 100. 100.] assert _one_arc("AAAAAAAAAAAAAAAAAABBBBBBBBBBBBBBBBBBBB", modality="mean", scale={  "A": [1],  "B": [0], }) == [77.14285714] | _one_arc("AAAAAAAAAAAAAAAAAABBBBBBBBBBBBBBBBBBBB", modality="mean", scale={  "A": [1],  "B": [0], }) = [81.] | The optimized version fixes the core bug in the original implementation: when the sequence length exceeds 18, the number of sliding windows was computed as len(seq) - window, missing one valid window. The corrected formula len(seq) - window + 1 ensures all windows are included. Additionally, the updated version adds minor input checks and replaces unsafe error-handling with proper exceptions, while keeping all descriptor logic strictly identical to the original code. | high | Boundary handling errors |
| WisecondorX(47) | def scale_sample(sample, from_size, to_size):  if not to_size or from_size == to_size:  return sample   if to_size == 0 or from_size == 0 or to_size < from_size or to_size % from_size > 0:  logging.critical(  "Impossible binsize scaling requested: {} to {}".format(  int(from_size), int(to_size)  )  )  sys.exit()   return_sample = dict()  scale = to_size / from_size  for chr_name in sample:  chr_data = sample[chr_name]  new_len = int(np.ceil(len(chr_data) / float(scale)))  scaled_chr = np.zeros(new_len, dtype=np.int32)  for i in range(new_len):  scaled_chr[i] = np.sum(chr_data[int(i * scale) : int(i * scale + scale)])  return_sample[chr_name] = scaled_chr  return return_sample | def scale_sample(sample, from_size, to_size):  if not to_size or from_size == to_size:  return sample   if to_size == 0 or from_size == 0 or to_size < from_size or to_size % from_size != 0:  raise ValueError(  f"Impossible binsize scaling requested: {from_size} to {to_size}"  )   return_sample = {}  scale = to_size // from_size   for chr_name, chr_data in sample.items():  new_len = int(np.ceil(len(chr_data) / scale))  scaled_chr = np.zeros(new_len, dtype=chr_data.dtype)   for i in range(new_len):  start = i * scale  end = min((i + 1) * scale, len(chr_data))  scaled_chr[i] = np.sum(chr_data[start:end])   return_sample[chr_name] = scaled_chr   return return_sample | assert scale_sample({"1": np.array([1, 2, 3, 4, 5, 6])}, 1, 2) == {'1': array([ 3, 7, 11])} assert scale_sample({"1": np.array([0.1, 0.2, 0.3, 0.4])}, 1, 2) == {'1': array([0.3, 0.7])} assert scale_sample({"1": np.array([1.5, 2.5, 3.5, 4.5])}, 1, 2) == {'1': array([4., 8.])} assert scale_sample({"1": np.array([0.05, 0.15, 0.25, 0.35])}, 1, 2) == {'1': array([0.2, 0.6])} | scale_sample({"1": np.array([0.1, 0.2, 0.3, 0.4])}, 1, 2) = {'1': array([0, 0])} scale_sample({"1": np.array([0.05, 0.15, 0.25, 0.35])}, 1, 2) = {'1': array([0, 0])} | The original implementation performs bin-size scaling using floating-point index truncation and overwrites chromosome-level results inside the inner loop, which can silently distort aggregated signals and lead to incorrect downstream analyses. | high | State Misalignment Error |
| SV-Bay(44) | def AbnRegion(link, read_length, median, biggest_normal, chrom1_line, chrom2_line, gem_line_chr1, gem_line_chr2, exp_direction):  shift = biggest_normal  if link.rightmost_begin + read_length - link.begin > shift or \  link.end - link.leftmost_end + read_length > shift:  shift = 2 * biggest_normal   if link.direction_type[0] == exp_direction[0]:  bp1_begin = link.rightmost_begin + read_length  bp1_end = link.begin + shift  else:  bp1_begin = link.rightmost_begin + read_length - shift  bp1_end = link.begin   if link.direction_type[1] == exp_direction[1]:  bp2_begin = link.end - shift  bp2_end = link.leftmost_end - read_length  else:  bp2_begin = link.end  bp2_end = link.leftmost_end - read_length + shift   # Calculate actual breakpoint simply as the middle of [bp_begin, bp_end]  bp1_mid = (bp1_begin + bp1_end) / 2  bp2_mid = (bp2_begin + bp2_end) / 2  bp1_size = bp1_end - bp1_begin  bp2_size = bp2_end - bp2_begin   abn_reg1 = [bp1_mid - shift, bp1_mid] if link.direction_type[0] == exp_direction[0] \  else [bp1_mid, bp1_mid + shift]  abn_reg2 = [bp2_mid, bp2_mid + shift] if link.direction_type[1] == exp_direction[1] \  else [bp2_mid - shift, bp2_mid]   abn_reg_chrom1 = chrom1_line[abn_reg1[0] : abn_reg1[1]]  abn_reg_chrom2 = chrom1_line[abn_reg2[0] : abn_reg2[1]]  abn_reg_gem1 = gem_line_chr1[abn_reg1[0] : abn_reg1[1]]  abn_reg_gem2 = gem_line_chr1[abn_reg2[0] : abn_reg2[1]]   return (abn_reg_chrom1, abn_reg_chrom2, abn_reg_gem1, abn_reg_gem2) | def AbnRegion(  link, read_length, median, biggest_normal,  chrom1_line, chrom2_line,  gem_line_chr1, gem_line_chr2,  exp_direction ):  shift = biggest_normal  if (link.rightmost_begin + read_length - link.begin > shift or  link.end - link.leftmost_end + read_length > shift):  shift = 2 * biggest_normal   # bp1  if link.direction_type[0] == exp_direction[0]:  bp1_begin = link.rightmost_begin + read_length  bp1_end = link.begin + shift  else:  bp1_begin = link.rightmost_begin + read_length - shift  bp1_end = link.begin   # bp2  if link.direction_type[1] == exp_direction[1]:  bp2_begin = link.end - shift  bp2_end = link.leftmost_end - read_length  else:  bp2_begin = link.end  bp2_end = link.leftmost_end - read_length + shift   bp1_begin, bp1_end = sorted([bp1_begin, bp1_end])  bp2_begin, bp2_end = sorted([bp2_begin, bp2_end])   bp1_mid = int((bp1_begin + bp1_end) // 2)  bp2_mid = int((bp2_begin + bp2_end) // 2)   if link.direction_type[0] == exp_direction[0]:  abn_reg1 = (bp1_mid - shift, bp1_mid)  else:  abn_reg1 = (bp1_mid, bp1_mid + shift)   if link.direction_type[1] == exp_direction[1]:  abn_reg2 = (bp2_mid, bp2_mid + shift)  else:  abn_reg2 = (bp2_mid - shift, bp2_mid)   def clip(a, b, n):  return max(0, a), min(b, n)   a1, b1 = clip(abn_reg1[0], abn_reg1[1], len(chrom1_line))  a2, b2 = clip(abn_reg2[0], abn_reg2[1], len(chrom2_line))   return (  chrom1_line[a1:b1],  chrom2_line[a2:b2],  gem_line_chr1[a1:b1],  gem_line_chr2[a2:b2],  ) class Link:  def __init__(self):  self.rightmost_begin = 10  self.begin = 20  self.end = 80  self.leftmost_end = 70  self.direction_type = ("+", "-")  link = Link() | assert AbnRegion(  link, 5, 0, 10,  np.arange(100), np.arange(1000, 1100),  np.ones(100), np.ones(100) * 2,  ("+", "-") ) == (array([ 7, 8, 9, 10, 11, 12, 13, 14, 15, 16, 17, 18, 19, 20, 21, 22, 23,  24, 25, 26]), array([1062, 1063, 1064, 1065, 1066, 1067, 1068, 1069, 1070, 1071, 1072,  1073, 1074, 1075, 1076, 1077, 1078, 1079, 1080, 1081]), array([1., 1., 1., 1., 1., 1., 1., 1., 1., 1., 1., 1., 1., 1., 1., 1., 1.,  1., 1., 1.]), array([2., 2., 2., 2., 2., 2., 2., 2., 2., 2., 2., 2., 2., 2., 2., 2., 2.,  2., 2., 2.])) | AbnRegion(  link, 5, 0, 10,  np.arange(100), np.arange(1000, 1100),  np.ones(100), np.ones(100) * 2,  ("+", "-") ) = (array([ 7, 8, 9, 10, 11, 12, 13, 14, 15, 16, 17, 18, 19, 20, 21, 22, 23,  24, 25, 26]), array([62, 63, 64, 65, 66, 67, 68, 69, 70, 71, 72, 73, 74, 75, 76, 77, 78,  79, 80, 81]), array([1., 1., 1., 1., 1., 1., 1., 1., 1., 1., 1., 1., 1., 1., 1., 1., 1.,  1., 1., 1.]), array([1., 1., 1., 1., 1., 1., 1., 1., 1., 1., 1., 1., 1., 1., 1., 1., 1.,  1., 1., 1.])) | The function contains a deterministic copy–paste bug in which the abnormal regions for the second chromosome are incorrectly extracted from chrom1_line and gem_line_chr1 instead of chrom2_line and gem_line_chr2, leading to systematic cross-chromosomal mislocalization of structural variants. In addition, breakpoint midpoints are computed as floating-point values and directly used as slice indices, which is invalid in Python 3 and can cause runtime errors or incorrect region slicing. | high | State Misalignment Error |
| SV-Bay(44) | def PrimarySplit(fragments, D, M):  clusters = []  while len(fragments) != 0:  upper_limit = fragments[0].length  lower_limit = fragments[0].length  frag_zero = fragments[0]  fragments.remove(frag_zero)  cluster = []  fragments_to_rm = []  cluster.append(frag_zero)  logger.debug('frag_zero.middle = ' + str(frag_zero.middle) + ' len =' + str(frag_zero.length))  for frag in fragments:  if frag.middle - frag_zero.middle <= D:  if frag.length > upper_limit and frag.length - lower_limit <= M:  upper_limit = frag.length  cluster.append(frag)  fragments_to_rm.append(frag)  frag_zero = frag  elif frag.length < lower_limit and upper_limit - frag.length <= M:  lower_limit = frag.length  cluster.append(frag)  fragments_to_rm.append(frag)  frag_zero = frag  elif upper_limit >= frag.length >= lower_limit:  cluster.append(frag)  fragments_to_rm.append(frag)  frag_zero = frag  else:  logger.debug('skiped fragment ' + str(frag.middle) + ' ' + str(frag.length))  logger.debug('upper_limit = ' + str(upper_limit) + ' lower_limit = ' + str(lower_limit))  else:  logger.debug('Here I break')  logger.debug('frag_zero.middle = ' + str(frag_zero.middle))  logger.debug('break fragment ' + str(frag.middle) + ' ' + str(frag.length))  break   # Logging below is commented, beacause it consumes time when called (even if INFO level is set)  # Uncomment if needed for debugging  # logger.debug('the case of else = ' + str(int(frag.middle) - int(frag_zero.middle)))  # logger.debug('frag current middle = ' + str(frag.middle))  # logger.debug('frag_zero.middle = ' + str(frag_zero.middle))  if len(cluster) > 1:  clusters.append(sorted(cluster, key=sortFragMid))  # logger.debug('Stop, print new cluster')  # for frag in cluster:  # logger.debug('frag.middle = ' + str(frag.middle) + ', frag.length = ' + str(frag.length))  for frag in fragments_to_rm:  fragments.remove(frag)    logger.debug('Clusters in total: ' + str(len(clusters)))  return clusters | def PrimarySplit(fragments, D, M):  clusters = []   fragments = sorted(fragments, key=lambda x: x.middle)   while fragments:  seed = fragments[0]  seed_middle = seed.middle   upper_limit = seed.length  lower_limit = seed.length   cluster = [seed]  fragments_to_rm = [seed]   for frag in fragments[1:]:  if frag.middle - seed_middle > D:  break   if frag.length > upper_limit and frag.length - lower_limit <= M:  upper_limit = frag.length  cluster.append(frag)  fragments_to_rm.append(frag)   elif frag.length < lower_limit and upper_limit - frag.length <= M:  lower_limit = frag.length  cluster.append(frag)  fragments_to_rm.append(frag)   elif lower_limit <= frag.length <= upper_limit:  cluster.append(frag)  fragments_to_rm.append(frag)   if len(cluster) > 1:  clusters.append(sorted(cluster, key=sortFragMid))   for frag in fragments_to_rm:  fragments.remove(frag)   return clusters class Frag:  def __init__(self, middle, length):  self.middle = middle  self.length = length  def __repr__(self):  return f"(m={self.middle}, l={self.length})"  def sortFragMid(f):  return f.middle | assert PrimarySplit([  Frag(0, 100),  Frag(8, 100),  Frag(16, 100),  ], 10, 50) == [[(m=0, l=100), (m=8, l=100)]] | PrimarySplit([  Frag(0, 100),  Frag(8, 100),  Frag(16, 100),  ], 10, 50) = [[(m=0, l=100), (m=8, l=100), (m=16, l=100)]] | PrimarySplit incorrectly updates the cluster seed during expansion, causing the distance constraint to drift and allowing clusters to grow beyond the specified threshold. | medium | alternative implementation |
| SV-Bay(44) | def IsOverlaps(link,links,tr_flag,biggest_normal):   overlaps= []  if not tr_flag:  for cl in links:  if cl == link:  continue  if link.begin <=cl.begin<=link.end and cl.end >= link.end and cl.gamma_alelles-3<=link.gamma_alelles<=cl.gamma_alelles+3:  overlaps.append(cl)  elif link.end<cl.begin:  break  else:  for cl in links:  if cl == link:  continue  else:  if cl.chr1 == link.chr1:  beg = abs(cl.begin-link.begin)  end = abs(cl.end-link.end)  if (beg<=2*biggest_normal or end<=2*biggest_normal): #or (0<link.begin-cl.begin<=2*biggest_normal or link.end-cl.end<= 2*biggest_normal):  overlaps.append(cl)  else:  beg = abs(cl.end-link.begin)  end = abs(link.end-cl.begin)  if (beg<=2*biggest_normal or end<=2*biggest_normal):  #if beg and end and (cl.end-link.begin<=2*biggest_normal or -link.end+cl.begin<= 2*biggest_normal): #or (0<-cl.end+link.begin<=2*biggest_normal or -link.end+cl.begin<= 2*biggest_normal):  overlaps.append(cl)  return overlaps | def IsOverlaps(link, links, tr_flag, biggest_normal):  overlaps = []   links = sorted(links, key=lambda x: x.begin)   if not tr_flag:  for cl in links:  if cl == link:  continue   if (  link.begin <= cl.begin <= link.end and  cl.end >= link.end and  cl.gamma_alelles - 3 <= link.gamma_alelles <= cl.gamma_alelles + 3  ):  overlaps.append(cl)   elif cl.begin > link.end:  break   else:  for cl in links:  if cl == link:  continue   if cl.chr1 == link.chr1:  dist = min(  abs(cl.begin - link.begin),  abs(cl.end - link.end)  )  else:  dist = min(  abs(cl.end - link.begin),  abs(link.end - cl.begin)  )   if dist <= 2 * biggest_normal:  overlaps.append(cl)   return overlaps class Link:  def __init__(self, begin, end, gamma, chr1):  self.begin = begin  self.end = end  self.gamma_alelles = gamma  self.chr1 = chr1  def __repr__(self):  return f"(b={self.begin}, e={self.end}, g={self.gamma_alelles})" | assert IsOverlaps(Link(100, 200, 10, 1),[  Link(300, 350, 10, 1),   Link(150, 250, 11, 1),  ], False, 50) == [(b=150, e=250, g=11)] assert IsOverlaps(Link(1000, 1100, 5, 1),[  Link(1000, 5000, 5, 1), ], False, 50) == [(b=1000, e=5000, g=5)] | IsOverlaps(Link(100, 200, 10, 1),[  Link(300, 350, 10, 1),   Link(150, 250, 11, 1),  ], False, 50) = [] | IsOverlaps relies on implicit input ordering and uses overly permissive distance conditions, leading to order-dependent behavior and systematic false-positive overlap detection. | low |  |
| Cell_BLAST(79) | def _compute_pcasd(  x: np.ndarray, x_posterior: np.ndarray, eps: float ) -> np.ndarray: # pragma: no cover  r"""  x : latent_dim  x_posterior : n_posterior * latent_dim  """  centered_x_posterior = (  x_posterior - np.sum(x_posterior, axis=0) / x_posterior.shape[0]  )  cov_x = np.dot(centered_x_posterior.T, centered_x_posterior)  v = np.real(  np.linalg.eig(cov_x.astype(np.complex64))[1]  ) # Suppress domain change due to rounding errors  x_posterior = np.dot(x_posterior - x, v)  squared_x_posterior = np.square(x_posterior)  asd = np.empty((2, x_posterior.shape[1]), dtype=np.float32)  for p in range(x_posterior.shape[1]):  mask = x_posterior[:, p] < 0  asd[0, p] = (  np.sqrt((np.sum(squared_x_posterior[mask, p])) / max(np.sum(mask), 1)) + eps  )  asd[1, p] = (  np.sqrt((np.sum(squared_x_posterior[~mask, p])) / max(np.sum(~mask), 1))  + eps  )  return np.concatenate((v, asd), axis=0) | def _compute_pcasd(  x: np.ndarray, x_posterior: np.ndarray, eps: float ) -> np.ndarray:  """  x : latent_dim  x_posterior : n_posterior * latent_dim  """  mu = np.mean(x_posterior, axis=0)  centered = x_posterior - mu   cov_x = np.dot(centered.T, centered) / max(centered.shape[0] - 1, 1)   eigvals, eigvecs = np.linalg.eigh(cov_x)   idx = np.argsort(eigvals)[::-1]  v = eigvecs[:, idx]   proj = np.dot(centered, v)  squared = proj ** 2   asd = np.empty((2, proj.shape[1]), dtype=np.float32)  for p in range(proj.shape[1]):  mask = proj[:, p] < 0  asd[0, p] = np.sqrt(np.mean(squared[mask, p]) if np.any(mask) else 0.0) + eps  asd[1, p] = np.sqrt(np.mean(squared[~mask, p]) if np.any(~mask) else 0.0) + eps   return np.concatenate((v, asd), axis=0) | assert _compute_pcasd(np.array([10.0, 10.0]), np.array([  [0.0, 0.0],  [1.0, 0.2],  [0.9, -0.1],  [1.1, 0.1], ]), 1e-6) == [[-0.99539324 0.09587651]  [-0.09587651 -0.99539324]  [ 0.26597875 0.07407983]  [ 0.75133973 0.16369146]] | _compute_pcasd(np.array([10.0, 10.0]), np.array([  [0.0, 0.0],  [1.0, 0.2],  [0.9, -0.1],  [1.1, 0.1], ]), 1e-6) = [[ 9.9539322e-01 -9.5876515e-02]  [ 9.5876515e-02 9.9539322e-01]  [ 1.0170910e+01 9.0179052e+00]  [ 1.0000000e-06 1.0000000e-06]] | The PCA-based decomposition is incorrectly centered at an arbitrary latent point rather than the posterior mean, and eigenvectors are computed using a non-symmetric solver, leading to unstable and statistically inconsistent ASD estimates. | medium | domain knowledge violation |
| Cell_BLAST(79) | def _npd_v2(  x: np.ndarray, y: np.ndarray, x_posterior: np.ndarray, eps: float ) -> np.ndarray: # pragma: no cover  r"""  x : latent_dim  y : latent_dim  x_posterior : n_posterior * latent_dim  """  if np.all(x == y):  return 0.0  dev = y - x  udev = dev / np.linalg.norm(dev)  projected_noise = np.sum((x_posterior - x) * udev, axis=1)  projected_y = np.sum((y - x) * udev)  mask = (projected_noise * projected_y) >= 0  scaler = np.sqrt(np.sum(np.square(projected_noise[mask])) / max(np.sum(mask), 1))  return np.abs(projected_y) / (scaler + eps) | def _npd_v2(  x: np.ndarray, y: np.ndarray, x_posterior: np.ndarray, eps: float ) -> float:  x = np.asarray(x, dtype=float)  y = np.asarray(y, dtype=float)  x_posterior = np.asarray(x_posterior, dtype=float)   dev = y - x  norm = np.linalg.norm(dev)   # Robust equality / degeneracy check  if norm < eps:  return 0.0   udev = dev / norm   projected_noise = np.dot(x_posterior - x, udev)  projected_y = norm # == dot(dev, udev)   # Use full variance, not one-sided truncated variance  var = np.mean(projected_noise ** 2)   return projected_y / (np.sqrt(var) + eps) | assert _npd_v2(np.array([1.0, 1.0]), np.array([1.0 + 1e-12, 1.0 + 1e-12]), np.array([  [1.01, 1.01],  [0.99, 0.99],  [1.00, 1.00], ]), 1e-8) == 0.0 assert _npd_v2(np.array([0.0, 0.0]), np.array([1.0, 0.0]), np.array([  [-1.0, 0.0],  [-2.0, 0.0],  [-3.0, 0.0], ]), 1e-6) == 0.46290983560066057 assert _npd_v2(np.array([1.0, 2.0, 3.0]), np.array([1.0, 2.0, 3.0]), np.array([  [1.1, 2.1, 3.1],  [0.9, 1.9, 2.9], ]), 1e-6) == 0.0 assert _npd_v2(np.array([0.0, 0.0]),np.array([1.0, 0.0]),np.array([  [ 0.5, 0.0],  [-0.5, 0.0],  [ 1.0, 0.0],  [-1.0, 0.0], ]), 1e-6) == 1.2649094640693754 assert _npd_v2(np.array([1.0, 1.0]),np.array([2.0, 1.0]),np.array([  [1.0, 1.0],  [1.0, 1.0],  [1.0, 1.0], ]), 1e-3) == 1000.0 | _npd_v2(np.array([1.0, 1.0]), np.array([1.0 + 1e-12, 1.0 + 1e-12]), np.array([  [1.01, 1.01],  [0.99, 0.99],  [1.00, 1.00], ]), 1e-8) = 1.414337872444471e-10  _npd_v2(np.array([0.0, 0.0]), np.array([1.0, 0.0]), np.array([  [-1.0, 0.0],  [-2.0, 0.0],  [-3.0, 0.0], ]), 1e-6) = 1000000.0 | The NPD computation is numerically unstable for nearly identical inputs and uses a one-sided truncated noise variance, leading to undefined directions and systematic overestimation. Equivalence between the two implementations only holds under degenerate or symmetric posterior distributions; otherwise, the original formulation systematically deviates. | medium | alternative implementation |
| Cell_BLAST(79) | def seurat_alignment_score(  x: np.ndarray,  y: np.ndarray,  k: float = 0.01,  n: int = 1,  metric: str = "minkowski",  random_seed: typing.Optional[int] = None,  n_jobs: int = 1, ) -> float:  random_state = np.random.RandomState(random_seed)  idx_list = [np.where(y == _y)[0] for _y in np.unique(y)]  subsample_size = min(idx.size for idx in idx_list)  subsample_scores = []  for _ in range(n):  subsample_idx_list = [  random_state.choice(idx, subsample_size, replace=False) for idx in idx_list  ]  subsample_y = y[np.concatenate(subsample_idx_list)]  subsample_x = x[np.concatenate(subsample_idx_list)]  _k = subsample_y.shape[0] * k if k < 1 else k  _k = np.round(_k).astype(int)  nearestNeighbors = sklearn.neighbors.NearestNeighbors(  n_neighbors=min(subsample_y.shape[0], _k + 1), metric=metric, n_jobs=n_jobs  )  nearestNeighbors.fit(subsample_x)  nni = nearestNeighbors.kneighbors(subsample_x, return_distance=False)  same_y_hits = (  (subsample_y[nni[:, 1:]] == np.expand_dims(subsample_y, axis=1))  .sum(axis=1)  .mean()  )  subsample_scores.append(  (_k - same_y_hits) * len(idx_list) / (_k * (len(idx_list) - 1))  )  return np.mean(subsample_scores) | def seurat_alignment_score(  x: np.ndarray,  y: np.ndarray,  k: float = 0.01,  n: int = 1,  metric: str = "minkowski",  random_seed: typing.Optional[int] = None,  n_jobs: int = 1, ) -> float:  random_state = np.random.RandomState(random_seed)   idx_list = [np.where(y == _y)[0] for _y in np.unique(y)]  subsample_size = min(len(idx) for idx in idx_list)   if subsample_size < 2:  raise ValueError("Each class must contain at least 2 samples.")   subsample_scores = []   for _ in range(n):  subsample_idx = np.concatenate(  [random_state.choice(idx, subsample_size, replace=False) for idx in idx_list]  )   subsample_x = x[subsample_idx]  subsample_y = y[subsample_idx]   _k = subsample_y.shape[0] * k if k < 1 else k  _k = int(max(1, round(_k)))    nn = sklearn.neighbors.NearestNeighbors(  n_neighbors=min(len(subsample_y), _k + 1),  metric=metric,  n_jobs=n_jobs,  )  nn.fit(subsample_x)  nni = nn.kneighbors(subsample_x, return_distance=False)   same_y_hits = np.mean([  np.sum(subsample_y[row[1:]] == subsample_y[i])  for i, row in enumerate(nni)  ])   score = (_k - same_y_hits) * len(idx_list) / (_k * (len(idx_list) - 1))  subsample_scores.append(score)   return float(np.mean(subsample_scores)) | assert seurat_alignment_score(np.random.randn(6, 2), np.array([0, 0, 0, 1, 1, 1]), k=0.01) == 1.0 assert seurat_alignment_score(np.array([  [0.0, 0.0],  [0.1, 0.0],  [5.0, 5.0],  [5.1, 5.0], ]), np.array([0, 0, 1, 1]), k=1) == 0.0 | seurat_alignment_score(np.random.randn(6, 2), np.array([0, 0, 0, 1, 1, 1]), k=0.01) = nan | The original implementation contains a deprecated NumPy API usage, allows zero-valued neighborhood sizes leading to invalid normalization, and implicitly assumes self-neighbors are always returned first, which does not hold under duplicate or degenerate samples. | low |  |
| Cell_BLAST(79) | def batch_mixing_entropy(  x: np.ndarray,  y: np.ndarray,  boots: int = 100,  sample_size: int = 100,  k: int = 100,  metric: str = "minkowski",  random_seed: typing.Optional[int] = None,  n_jobs: int = 1, ) -> float:  random_state = np.random.RandomState(random_seed)  batches = np.unique(y)  entropy = 0  for _ in range(boots):  bootsamples = random_state.choice(  np.arange(x.shape[0]), sample_size, replace=False  )  subsample_x = x[bootsamples]  neighbor = sklearn.neighbors.NearestNeighbors(  n_neighbors=k, metric=metric, n_jobs=n_jobs  )  neighbor.fit(x)  nn = neighbor.kneighbors(subsample_x, return_distance=False)  for i in range(sample_size):  for batch in batches:  b = len(np.where(y[nn[i, :]] == batch)[0]) / k  if b == 0:  entropy = entropy  else:  entropy = entropy + b * np.log(b)  entropy = -entropy / (boots * sample_size)  return entropy | def batch_mixing_entropy(  x: np.ndarray,  y: np.ndarray,  boots: int = 100,  sample_size: int = 100,  k: int = 100,  metric: str = "minkowski",  random_seed: typing.Optional[int] = None,  n_jobs: int = 1, ) -> float:  random_state = np.random.RandomState(random_seed)  n_samples = x.shape[0]   if sample_size > n_samples:  raise ValueError("sample_size must be <= number of samples")   if k >= n_samples:  raise ValueError("k must be < number of samples (self-neighbor exclusion)")   batches = np.unique(y)  total_entropy = 0.0   neighbor = sklearn.neighbors.NearestNeighbors(  n_neighbors=k + 1, metric=metric, n_jobs=n_jobs  )  neighbor.fit(x)   for _ in range(boots):  bootsamples = random_state.choice(n_samples, sample_size, replace=False)  subsample_x = x[bootsamples]  subsample_y = y[bootsamples]   nn = neighbor.kneighbors(subsample_x, return_distance=False)   for i in range(sample_size):  neighbors = nn[i, 1:]  local_entropy = 0.0   for batch in batches:  b = np.sum(y[neighbors] == batch) / k  if b > 0:  local_entropy += b * np.log(b)   total_entropy += local_entropy   return -total_entropy / (boots * sample_size) | assert batch_mixing_entropy(np.array([  [0.0, 0.0],  [0.1, 0.0],  [5.0, 5.0],  [5.1, 5.0], ]), np.array([0, 0, 1, 1]), boots=10, sample_size=4, k=1) == -0.0 assert batch_mixing_entropy(np.random.randn(20, 2), np.array([0]*10 + [1]*10), boots=10, sample_size=10, k=5) == 0.5685912938356554 assert batch_mixing_entropy(np.vstack([  np.random.randn(10, 2),  np.random.randn(10, 2) + 10 ]), np.array([0]*10 + [1]*10), boots=5, sample_size=10, k=3) == -0.0 assert batch_mixing_entropy(np.random.randn(30, 5), np.random.randint(0, 3, size=30), boots=5, sample_size=10, k=3) == 0.8178650954857009 | batch_mixing_entropy(np.random.randn(20, 2), np.array([0]*10 + [1]*10), boots=10, sample_size=10, k=5) = 0.603967969620831 batch_mixing_entropy(np.random.randn(30, 5), np.random.randint(0, 3, size=30), boots=5, sample_size=10, k=3) = 0.8028694916291984 | The original implementation systematically underestimates batch mixing entropy by including self-neighbors in kNN queries, misnormalizing batch probabilities, and lacking boundary checks for k and subsample size. | medium | domain knowledge violation |
| Cell_BLAST(79) | def get_volume(  batch: np.ndarray,  num_batch: int,  cluster: np.ndarray,  sum_cluster: int,  num_clusters: typing.List[int], ):  volume = np.zeros(sum_cluster)  for i in range(num_batch):  for j in range(num_clusters[i], num_clusters[i + 1]):  volume[j] = (cluster == j).sum() / (batch == i).sum()   return volume | def get_volume(  batch: np.ndarray,  num_batch: int,  cluster: np.ndarray,  sum_cluster: int,  num_clusters: typing.List[int], ):  if len(num_clusters) != num_batch + 1:  raise ValueError("num_clusters length must be num_batch + 1")   volume = np.zeros(sum_cluster, dtype=float)   for i in range(num_batch):  batch_mask = batch == i  batch_count = np.sum(batch_mask)   if batch_count == 0:  continue    for j in range(num_clusters[i], num_clusters[i + 1]):  volume[j] = np.sum(cluster[batch_mask] == j) / batch_count   return volume | assert get_volume(np.array([0, 0, 1, 1]), 2, np.array([0, 1, 0, 1]), 4, [0, 2, 4]) == [0.5 0.5 0. 0. ] assert get_volume(np.array([0, 0, 0]), 2, np.array([0, 1, 1]), 3, [0, 2, 3]) == [0.33333333 0.66666667 0. ] assert get_volume(np.array([0, 0, 1, 1]), 2, np.array([0, 0, 2, 2]), 3, [0, 1, 3]) == [1. 0. 1.] | get_volume(np.array([0, 0, 1, 1]), 2, np.array([0, 1, 0, 1]), 4, [0, 2, 4]) = [1. 1. 0. 0.] get_volume(np.array([0, 0, 0]), 2, np.array([0, 1, 1]), 3, [0, 2, 3]) = [0.33333333 0.66666667 nan] | The original implementation incorrectly computes cluster proportions by aggregating cluster counts across all batches while normalizing by per-batch sample sizes, and it may trigger division-by-zero errors when batches are empty. | low |  |
| Cyclum(42) | def parzen_estimate(x, lim, half_granularity=100,  window=lambda x, rho: sp.stats.norm.pdf(x, 0, rho), scale=0.5):  """Calculate parzen window estimation (a non-parametric density estimation method)   :param x: instances  :param lim: limit of domain  :param half_granularity:  :param window:  :param scale:  :return:  """  assert scale < 1 and scale > 0, "scale must be in (0, 1) to perform a unbiased estimation"  gran = half_granularity * 2 + 1;   n = len(x)  rho = n ** -scale   l = lim[1] - lim[0]  grid = np.linspace(lim[0] - l / 2, lim[1] + l / 2, half_granularity * 4 + 1)  discretized_window = window(grid, rho)  discretized_window = discretized_window / np.sum(discretized_window)   def individual(offset):  indi = np.roll(discretized_window, offset - half_granularity)  if offset > 0:  indi[0: offset] = 0  else:  indi[offset:] = 0  return indi   xx = np.round((x - lim[0] + l) / l / 2 * gran)  res = sum(map(individual, xx.astype(int).tolist())) / n  return grid, res | def parzen_estimate(  x,  lim,  half_granularity=100,  window=lambda x, rho: sp.stats.norm.pdf(x, 0, rho),  scale=0.5, ):  assert 0 < scale < 1   n = len(x)  rho = n ** -scale   l = lim[1] - lim[0]  grid = np.linspace(lim[0] - l / 2, lim[1] + l / 2, half_granularity * 4 + 1)  w = window(grid, rho)  w = w / w.sum()   gran = half_granularity * 2 + 1  center = len(w) // 2   res = np.zeros_like(w)   xx = np.round((x - lim[0]) / l * (gran - 1)).astype(int)   for offset in xx:  offset = np.clip(offset, 0, gran - 1)  start = center - offset  end = start + gran   if start < 0 or end > len(w):  continue   res[start:end] += w[center - offset : center - offset + gran]   res /= n  return grid, res | assert parzen_estimate(np.array([0.99]), (0.0, 1.0))== [-0.5 -0.495 -0.49 -0.485 -0.48 -0.475 -0.47 -0.465 -0.46 -0.455  -0.45 -0.445 -0.44 -0.435 -0.43 -0.425 -0.42 -0.415 -0.41 -0.405  -0.4 -0.395 -0.39 -0.385 -0.38 -0.375 -0.37 -0.365 -0.36 -0.355  -0.35 -0.345 -0.34 -0.335 -0.33 -0.325 -0.32 -0.315 -0.31 -0.305  -0.3 -0.295 -0.29 -0.285 -0.28 -0.275 -0.27 -0.265 -0.26 -0.255  -0.25 -0.245 -0.24 -0.235 -0.23 -0.225 -0.22 -0.215 -0.21 -0.205  -0.2 -0.195 -0.19 -0.185 -0.18 -0.175 -0.17 -0.165 -0.16 -0.155  -0.15 -0.145 -0.14 -0.135 -0.13 -0.125 -0.12 -0.115 -0.11 -0.105  -0.1 -0.095 -0.09 -0.085 -0.08 -0.075 -0.07 -0.065 -0.06 -0.055  -0.05 -0.045 -0.04 -0.035 -0.03 -0.025 -0.02 -0.015 -0.01 -0.005  0. 0.005 0.01 0.015 0.02 0.025 0.03 0.035 0.04 0.045  0.05 0.055 0.06 0.065 0.07 0.075 0.08 0.085 0.09 0.095  0.1 0.105 0.11 0.115 0.12 0.125 0.13 0.135 0.14 0.145  0.15 0.155 0.16 0.165 0.17 0.175 0.18 0.185 0.19 0.195  0.2 0.205 0.21 0.215 0.22 0.225 0.23 0.235 0.24 0.245  0.25 0.255 0.26 0.265 0.27 0.275 0.28 0.285 0.29 0.295  0.3 0.305 0.31 0.315 0.32 0.325 0.33 0.335 0.34 0.345  0.35 0.355 0.36 0.365 0.37 0.375 0.38 0.385 0.39 0.395  0.4 0.405 0.41 0.415 0.42 0.425 0.43 0.435 0.44 0.445  0.45 0.455 0.46 0.465 0.47 0.475 0.48 0.485 0.49 0.495  0.5 0.505 0.51 0.515 0.52 0.525 0.53 0.535 0.54 0.545  0.55 0.555 0.56 0.565 0.57 0.575 0.58 0.585 0.59 0.595  0.6 0.605 0.61 0.615 0.62 0.625 0.63 0.635 0.64 0.645  0.65 0.655 0.66 0.665 0.67 0.675 0.68 0.685 0.69 0.695  0.7 0.705 0.71 0.715 0.72 0.725 0.73 0.735 0.74 0.745  0.75 0.755 0.76 0.765 0.77 0.775 0.78 0.785 0.79 0.795  0.8 0.805 0.81 0.815 0.82 0.825 0.83 0.835 0.84 0.845  0.85 0.855 0.86 0.865 0.87 0.875 0.88 0.885 0.89 0.895  0.9 0.905 0.91 0.915 0.92 0.925 0.93 0.935 0.94 0.945  0.95 0.955 0.96 0.965 0.97 0.975 0.98 0.985 0.99 0.995  1. 1.005 1.01 1.015 1.02 1.025 1.03 1.035 1.04 1.045  1.05 1.055 1.06 1.065 1.07 1.075 1.08 1.085 1.09 1.095  1.1 1.105 1.11 1.115 1.12 1.125 1.13 1.135 1.14 1.145  1.15 1.155 1.16 1.165 1.17 1.175 1.18 1.185 1.19 1.195  1.2 1.205 1.21 1.215 1.22 1.225 1.23 1.235 1.24 1.245  1.25 1.255 1.26 1.265 1.27 1.275 1.28 1.285 1.29 1.295  1.3 1.305 1.31 1.315 1.32 1.325 1.33 1.335 1.34 1.345  1.35 1.355 1.36 1.365 1.37 1.375 1.38 1.385 1.39 1.395  1.4 1.405 1.41 1.415 1.42 1.425 1.43 1.435 1.44 1.445  1.45 1.455 1.46 1.465 1.47 1.475 1.48 1.485 1.49 1.495  1.5 ] [0. 0. 0.00282662 0.00283351 0.00284036 0.00284715  0.00285388 0.00286056 0.00286718 0.00287375 0.00288026 0.00288671  0.00289311 0.00289944 0.00290572 0.00291194 0.00291809 0.00292419  0.00293023 0.00293621 0.00294212 0.00294797 0.00295376 0.00295949  0.00296516 0.00297076 0.0029763 0.00298177 0.00298718 0.00299253  0.00299781 0.00300302 0.00300817 0.00301325 0.00301826 0.00302321  0.00302809 0.0030329 0.00303764 0.00304231 0.00304692 0.00305145  0.00305592 0.00306032 0.00306464 0.0030689 0.00307308 0.00307719  0.00308123 0.0030852 0.0030891 0.00309293 0.00309668 0.00310036  0.00310397 0.0031075 0.00311096 0.00311434 0.00311765 0.00312089  0.00312405 0.00312714 0.00313015 0.00313308 0.00313594 0.00313873  0.00314144 0.00314407 0.00314662 0.0031491 0.00315151 0.00315383  0.00315608 0.00315825 0.00316034 0.00316236 0.0031643 0.00316616  0.00316794 0.00316964 0.00317126 0.00317281 0.00317428 0.00317567  0.00317698 0.00317821 0.00317936 0.00318043 0.00318143 0.00318234  0.00318318 0.00318394 0.00318461 0.00318521 0.00318573 0.00318616  0.00318652 0.0031868 0.003187 0.00318712 0.00318716 0.00318712  0.003187 0.0031868 0.00318652 0.00318616 0.00318573 0.00318521  0.00318461 0.00318394 0.00318318 0.00318234 0.00318143 0.00318043  0.00317936 0.00317821 0.00317698 0.00317567 0.00317428 0.00317281  0.00317126 0.00316964 0.00316794 0.00316616 0.0031643 0.00316236  0.00316034 0.00315825 0.00315608 0.00315383 0.00315151 0.0031491  0.00314662 0.00314407 0.00314144 0.00313873 0.00313594 0.00313308  0.00313015 0.00312714 0.00312405 0.00312089 0.00311765 0.00311434  0.00311096 0.0031075 0.00310397 0.00310036 0.00309668 0.00309293  0.0030891 0.0030852 0.00308123 0.00307719 0.00307308 0.0030689  0.00306464 0.00306032 0.00305592 0.00305145 0.00304692 0.00304231  0.00303764 0.0030329 0.00302809 0.00302321 0.00301826 0.00301325  0.00300817 0.00300302 0.00299781 0.00299253 0.00298718 0.00298177  0.0029763 0.00297076 0.00296516 0.00295949 0.00295376 0.00294797  0.00294212 0.00293621 0.00293023 0.00292419 0.00291809 0.00291194  0.00290572 0.00289944 0.00289311 0.00288671 0.00288026 0.00287375  0.00286718 0.00286056 0.00285388 0.00284715 0.00284036 0.00283351  0.00282662 0.00281966 0.00281266 0.0028056 0.00279849 0.  0. 0. 0. 0. 0. 0.  0. 0. 0. 0. 0. 0.  0. 0. 0. 0. 0. 0.  0. 0. 0. 0. 0. 0.  0. 0. 0. 0. 0. 0.  0. 0. 0. 0. 0. 0.  0. 0. 0. 0. 0. 0.  0. 0. 0. 0. 0. 0.  0. 0. 0. 0. 0. 0.  0. 0. 0. 0. 0. 0.  0. 0. 0. 0. 0. 0.  0. 0. 0. 0. 0. 0.  0. 0. 0. 0. 0. 0.  0. 0. 0. 0. 0. 0.  0. 0. 0. 0. 0. 0.  0. 0. 0. 0. 0. 0.  0. 0. 0. 0. 0. 0.  0. 0. 0. 0. 0. 0.  0. 0. 0. 0. 0. 0.  0. 0. 0. 0. 0. 0.  0. 0. 0. 0. 0. 0.  0. 0. 0. 0. 0. 0.  0. 0. 0. 0. 0. 0.  0. 0. 0. 0. 0. 0.  0. 0. 0. 0. 0. 0.  0. 0. 0. 0. 0. 0.  0. 0. 0. 0. 0. 0.  0. 0. 0. 0. 0. 0.  0. 0. 0. 0. 0. 0.  0. 0. 0. 0. 0. 0.  0. 0. 0. 0. 0. 0.  0. 0. 0. 0. 0. 0.  0. 0. 0. 0. 0. ] assert parzen_estimate(np.array([2.0]), (0.0, 1.0)) == (array([-0.5 , -0.495, -0.49 , -0.485, -0.48 , -0.475, -0.47 , -0.465,  -0.46 , -0.455, -0.45 , -0.445, -0.44 , -0.435, -0.43 , -0.425,  -0.42 , -0.415, -0.41 , -0.405, -0.4 , -0.395, -0.39 , -0.385,  -0.38 , -0.375, -0.37 , -0.365, -0.36 , -0.355, -0.35 , -0.345,  -0.34 , -0.335, -0.33 , -0.325, -0.32 , -0.315, -0.31 , -0.305,  -0.3 , -0.295, -0.29 , -0.285, -0.28 , -0.275, -0.27 , -0.265,  -0.26 , -0.255, -0.25 , -0.245, -0.24 , -0.235, -0.23 , -0.225,  -0.22 , -0.215, -0.21 , -0.205, -0.2 , -0.195, -0.19 , -0.185,  -0.18 , -0.175, -0.17 , -0.165, -0.16 , -0.155, -0.15 , -0.145,  -0.14 , -0.135, -0.13 , -0.125, -0.12 , -0.115, -0.11 , -0.105,  -0.1 , -0.095, -0.09 , -0.085, -0.08 , -0.075, -0.07 , -0.065,  -0.06 , -0.055, -0.05 , -0.045, -0.04 , -0.035, -0.03 , -0.025,  -0.02 , -0.015, -0.01 , -0.005, 0. , 0.005, 0.01 , 0.015,  0.02 , 0.025, 0.03 , 0.035, 0.04 , 0.045, 0.05 , 0.055,  0.06 , 0.065, 0.07 , 0.075, 0.08 , 0.085, 0.09 , 0.095,  0.1 , 0.105, 0.11 , 0.115, 0.12 , 0.125, 0.13 , 0.135,  0.14 , 0.145, 0.15 , 0.155, 0.16 , 0.165, 0.17 , 0.175,  0.18 , 0.185, 0.19 , 0.195, 0.2 , 0.205, 0.21 , 0.215,  0.22 , 0.225, 0.23 , 0.235, 0.24 , 0.245, 0.25 , 0.255,  0.26 , 0.265, 0.27 , 0.275, 0.28 , 0.285, 0.29 , 0.295,  0.3 , 0.305, 0.31 , 0.315, 0.32 , 0.325, 0.33 , 0.335,  0.34 , 0.345, 0.35 , 0.355, 0.36 , 0.365, 0.37 , 0.375,  0.38 , 0.385, 0.39 , 0.395, 0.4 , 0.405, 0.41 , 0.415,  0.42 , 0.425, 0.43 , 0.435, 0.44 , 0.445, 0.45 , 0.455,  0.46 , 0.465, 0.47 , 0.475, 0.48 , 0.485, 0.49 , 0.495,  0.5 , 0.505, 0.51 , 0.515, 0.52 , 0.525, 0.53 , 0.535,  0.54 , 0.545, 0.55 , 0.555, 0.56 , 0.565, 0.57 , 0.575,  0.58 , 0.585, 0.59 , 0.595, 0.6 , 0.605, 0.61 , 0.615,  0.62 , 0.625, 0.63 , 0.635, 0.64 , 0.645, 0.65 , 0.655,  0.66 , 0.665, 0.67 , 0.675, 0.68 , 0.685, 0.69 , 0.695,  0.7 , 0.705, 0.71 , 0.715, 0.72 , 0.725, 0.73 , 0.735,  0.74 , 0.745, 0.75 , 0.755, 0.76 , 0.765, 0.77 , 0.775,  0.78 , 0.785, 0.79 , 0.795, 0.8 , 0.805, 0.81 , 0.815,  0.82 , 0.825, 0.83 , 0.835, 0.84 , 0.845, 0.85 , 0.855,  0.86 , 0.865, 0.87 , 0.875, 0.88 , 0.885, 0.89 , 0.895,  0.9 , 0.905, 0.91 , 0.915, 0.92 , 0.925, 0.93 , 0.935,  0.94 , 0.945, 0.95 , 0.955, 0.96 , 0.965, 0.97 , 0.975,  0.98 , 0.985, 0.99 , 0.995, 1. , 1.005, 1.01 , 1.015,  1.02 , 1.025, 1.03 , 1.035, 1.04 , 1.045, 1.05 , 1.055,  1.06 , 1.065, 1.07 , 1.075, 1.08 , 1.085, 1.09 , 1.095,  1.1 , 1.105, 1.11 , 1.115, 1.12 , 1.125, 1.13 , 1.135,  1.14 , 1.145, 1.15 , 1.155, 1.16 , 1.165, 1.17 , 1.175,  1.18 , 1.185, 1.19 , 1.195, 1.2 , 1.205, 1.21 , 1.215,  1.22 , 1.225, 1.23 , 1.235, 1.24 , 1.245, 1.25 , 1.255,  1.26 , 1.265, 1.27 , 1.275, 1.28 , 1.285, 1.29 , 1.295,  1.3 , 1.305, 1.31 , 1.315, 1.32 , 1.325, 1.33 , 1.335,  1.34 , 1.345, 1.35 , 1.355, 1.36 , 1.365, 1.37 , 1.375,  1.38 , 1.385, 1.39 , 1.395, 1.4 , 1.405, 1.41 , 1.415,  1.42 , 1.425, 1.43 , 1.435, 1.44 , 1.445, 1.45 , 1.455,  1.46 , 1.465, 1.47 , 1.475, 1.48 , 1.485, 1.49 , 1.495,  1.5 ]), array([0.00281266, 0.00281966, 0.00282662, 0.00283351, 0.00284036,  0.00284715, 0.00285388, 0.00286056, 0.00286718, 0.00287375,  0.00288026, 0.00288671, 0.00289311, 0.00289944, 0.00290572,  0.00291194, 0.00291809, 0.00292419, 0.00293023, 0.00293621,  0.00294212, 0.00294797, 0.00295376, 0.00295949, 0.00296516,  0.00297076, 0.0029763 , 0.00298177, 0.00298718, 0.00299253,  0.00299781, 0.00300302, 0.00300817, 0.00301325, 0.00301826,  0.00302321, 0.00302809, 0.0030329 , 0.00303764, 0.00304231,  0.00304692, 0.00305145, 0.00305592, 0.00306032, 0.00306464,  0.0030689 , 0.00307308, 0.00307719, 0.00308123, 0.0030852 ,  0.0030891 , 0.00309293, 0.00309668, 0.00310036, 0.00310397,  0.0031075 , 0.00311096, 0.00311434, 0.00311765, 0.00312089,  0.00312405, 0.00312714, 0.00313015, 0.00313308, 0.00313594,  0.00313873, 0.00314144, 0.00314407, 0.00314662, 0.0031491 ,  0.00315151, 0.00315383, 0.00315608, 0.00315825, 0.00316034,  0.00316236, 0.0031643 , 0.00316616, 0.00316794, 0.00316964,  0.00317126, 0.00317281, 0.00317428, 0.00317567, 0.00317698,  0.00317821, 0.00317936, 0.00318043, 0.00318143, 0.00318234,  0.00318318, 0.00318394, 0.00318461, 0.00318521, 0.00318573,  0.00318616, 0.00318652, 0.0031868 , 0.003187 , 0.00318712,  0.00318716, 0.00318712, 0.003187 , 0.0031868 , 0.00318652,  0.00318616, 0.00318573, 0.00318521, 0.00318461, 0.00318394,  0.00318318, 0.00318234, 0.00318143, 0.00318043, 0.00317936,  0.00317821, 0.00317698, 0.00317567, 0.00317428, 0.00317281,  0.00317126, 0.00316964, 0.00316794, 0.00316616, 0.0031643 ,  0.00316236, 0.00316034, 0.00315825, 0.00315608, 0.00315383,  0.00315151, 0.0031491 , 0.00314662, 0.00314407, 0.00314144,  0.00313873, 0.00313594, 0.00313308, 0.00313015, 0.00312714,  0.00312405, 0.00312089, 0.00311765, 0.00311434, 0.00311096,  0.0031075 , 0.00310397, 0.00310036, 0.00309668, 0.00309293,  0.0030891 , 0.0030852 , 0.00308123, 0.00307719, 0.00307308,  0.0030689 , 0.00306464, 0.00306032, 0.00305592, 0.00305145,  0.00304692, 0.00304231, 0.00303764, 0.0030329 , 0.00302809,  0.00302321, 0.00301826, 0.00301325, 0.00300817, 0.00300302,  0.00299781, 0.00299253, 0.00298718, 0.00298177, 0.0029763 ,  0.00297076, 0.00296516, 0.00295949, 0.00295376, 0.00294797,  0.00294212, 0.00293621, 0.00293023, 0.00292419, 0.00291809,  0.00291194, 0.00290572, 0.00289944, 0.00289311, 0.00288671,  0.00288026, 0.00287375, 0.00286718, 0.00286056, 0.00285388,  0.00284715, 0.00284036, 0.00283351, 0.00282662, 0.00281966,  0.00281266, 0. , 0. , 0. , 0. ,  0. , 0. , 0. , 0. , 0. ,  0. , 0. , 0. , 0. , 0. ,  0. , 0. , 0. , 0. , 0. ,  0. , 0. , 0. , 0. , 0. ,  0. , 0. , 0. , 0. , 0. ,  0. , 0. , 0. , 0. , 0. ,  0. , 0. , 0. , 0. , 0. ,  0. , 0. , 0. , 0. , 0. ,  0. , 0. , 0. , 0. , 0. ,  0. , 0. , 0. , 0. , 0. ,  0. , 0. , 0. , 0. , 0. ,  0. , 0. , 0. , 0. , 0. ,  0. , 0. , 0. , 0. , 0. ,  0. , 0. , 0. , 0. , 0. ,  0. , 0. , 0. , 0. , 0. ,  0. , 0. , 0. , 0. , 0. ,  0. , 0. , 0. , 0. , 0. ,  0. , 0. , 0. , 0. , 0. ,  0. , 0. , 0. , 0. , 0. ,  0. , 0. , 0. , 0. , 0. ,  0. , 0. , 0. , 0. , 0. ,  0. , 0. , 0. , 0. , 0. ,  0. , 0. , 0. , 0. , 0. ,  0. , 0. , 0. , 0. , 0. ,  0. , 0. , 0. , 0. , 0. ,  0. , 0. , 0. , 0. , 0. ,  0. , 0. , 0. , 0. , 0. ,  0. , 0. , 0. , 0. , 0. ,  0. , 0. , 0. , 0. , 0. ,  0. , 0. , 0. , 0. , 0. ,  0. , 0. , 0. , 0. , 0. ,  0. , 0. , 0. , 0. , 0. ,  0. , 0. , 0. , 0. , 0. ,  0. , 0. , 0. , 0. , 0. ,  0. , 0. , 0. , 0. , 0. ,  0. , 0. , 0. , 0. , 0. ,  0. , 0. , 0. , 0. , 0. ,  0. , 0. , 0. , 0. , 0. ,  0. , 0. , 0. , 0. , 0. ,  0. ])) | parzen_estimate(np.array([0.99]), (0.0, 1.0))= [-0.5 -0.495 -0.49 -0.485 -0.48 -0.475 -0.47 -0.465 -0.46 -0.455  -0.45 -0.445 -0.44 -0.435 -0.43 -0.425 -0.42 -0.415 -0.41 -0.405  -0.4 -0.395 -0.39 -0.385 -0.38 -0.375 -0.37 -0.365 -0.36 -0.355  -0.35 -0.345 -0.34 -0.335 -0.33 -0.325 -0.32 -0.315 -0.31 -0.305  -0.3 -0.295 -0.29 -0.285 -0.28 -0.275 -0.27 -0.265 -0.26 -0.255  -0.25 -0.245 -0.24 -0.235 -0.23 -0.225 -0.22 -0.215 -0.21 -0.205  -0.2 -0.195 -0.19 -0.185 -0.18 -0.175 -0.17 -0.165 -0.16 -0.155  -0.15 -0.145 -0.14 -0.135 -0.13 -0.125 -0.12 -0.115 -0.11 -0.105  -0.1 -0.095 -0.09 -0.085 -0.08 -0.075 -0.07 -0.065 -0.06 -0.055  -0.05 -0.045 -0.04 -0.035 -0.03 -0.025 -0.02 -0.015 -0.01 -0.005  0. 0.005 0.01 0.015 0.02 0.025 0.03 0.035 0.04 0.045  0.05 0.055 0.06 0.065 0.07 0.075 0.08 0.085 0.09 0.095  0.1 0.105 0.11 0.115 0.12 0.125 0.13 0.135 0.14 0.145  0.15 0.155 0.16 0.165 0.17 0.175 0.18 0.185 0.19 0.195  0.2 0.205 0.21 0.215 0.22 0.225 0.23 0.235 0.24 0.245  0.25 0.255 0.26 0.265 0.27 0.275 0.28 0.285 0.29 0.295  0.3 0.305 0.31 0.315 0.32 0.325 0.33 0.335 0.34 0.345  0.35 0.355 0.36 0.365 0.37 0.375 0.38 0.385 0.39 0.395  0.4 0.405 0.41 0.415 0.42 0.425 0.43 0.435 0.44 0.445  0.45 0.455 0.46 0.465 0.47 0.475 0.48 0.485 0.49 0.495  0.5 0.505 0.51 0.515 0.52 0.525 0.53 0.535 0.54 0.545  0.55 0.555 0.56 0.565 0.57 0.575 0.58 0.585 0.59 0.595  0.6 0.605 0.61 0.615 0.62 0.625 0.63 0.635 0.64 0.645  0.65 0.655 0.66 0.665 0.67 0.675 0.68 0.685 0.69 0.695  0.7 0.705 0.71 0.715 0.72 0.725 0.73 0.735 0.74 0.745  0.75 0.755 0.76 0.765 0.77 0.775 0.78 0.785 0.79 0.795  0.8 0.805 0.81 0.815 0.82 0.825 0.83 0.835 0.84 0.845  0.85 0.855 0.86 0.865 0.87 0.875 0.88 0.885 0.89 0.895  0.9 0.905 0.91 0.915 0.92 0.925 0.93 0.935 0.94 0.945  0.95 0.955 0.96 0.965 0.97 0.975 0.98 0.985 0.99 0.995  1. 1.005 1.01 1.015 1.02 1.025 1.03 1.035 1.04 1.045  1.05 1.055 1.06 1.065 1.07 1.075 1.08 1.085 1.09 1.095  1.1 1.105 1.11 1.115 1.12 1.125 1.13 1.135 1.14 1.145  1.15 1.155 1.16 1.165 1.17 1.175 1.18 1.185 1.19 1.195  1.2 1.205 1.21 1.215 1.22 1.225 1.23 1.235 1.24 1.245  1.25 1.255 1.26 1.265 1.27 1.275 1.28 1.285 1.29 1.295  1.3 1.305 1.31 1.315 1.32 1.325 1.33 1.335 1.34 1.345  1.35 1.355 1.36 1.365 1.37 1.375 1.38 1.385 1.39 1.395  1.4 1.405 1.41 1.415 1.42 1.425 1.43 1.435 1.44 1.445  1.45 1.455 1.46 1.465 1.47 1.475 1.48 1.485 1.49 1.495  1.5 ] [0. 0. 0. 0. 0. 0.  0. 0. 0. 0. 0. 0.  0. 0. 0. 0. 0. 0.  0. 0. 0. 0. 0. 0.  0. 0. 0. 0. 0. 0.  0. 0. 0. 0. 0. 0.  0. 0. 0. 0. 0. 0.  0. 0. 0. 0. 0. 0.  0. 0. 0. 0. 0. 0.  0. 0. 0. 0. 0. 0.  0. 0. 0. 0. 0. 0.  0. 0. 0. 0. 0. 0.  0. 0. 0. 0. 0. 0.  0. 0. 0. 0. 0. 0.  0. 0. 0. 0. 0. 0.  0. 0. 0. 0. 0. 0.  0. 0. 0. 0. 0. 0.  0. 0. 0. 0. 0. 0.  0. 0. 0. 0. 0. 0.  0. 0. 0. 0. 0. 0.  0. 0. 0. 0. 0. 0.  0. 0. 0. 0. 0. 0.  0. 0. 0. 0. 0. 0.  0. 0. 0. 0. 0. 0.  0. 0. 0. 0. 0. 0.  0. 0. 0. 0. 0. 0.  0. 0. 0. 0. 0. 0.  0. 0. 0. 0. 0. 0.  0. 0. 0. 0. 0. 0.  0. 0. 0. 0. 0. 0.  0. 0. 0. 0. 0. 0.  0. 0. 0. 0. 0. 0.  0. 0. 0. 0. 0. 0.  0. 0. 0.00318716 0.00318712 0.003187 0.0031868  0.00318652 0.00318616 0.00318573 0.00318521 0.00318461 0.00318394  0.00318318 0.00318234 0.00318143 0.00318043 0.00317936 0.00317821  0.00317698 0.00317567 0.00317428 0.00317281 0.00317126 0.00316964  0.00316794 0.00316616 0.0031643 0.00316236 0.00316034 0.00315825  0.00315608 0.00315383 0.00315151 0.0031491 0.00314662 0.00314407  0.00314144 0.00313873 0.00313594 0.00313308 0.00313015 0.00312714  0.00312405 0.00312089 0.00311765 0.00311434 0.00311096 0.0031075  0.00310397 0.00310036 0.00309668 0.00309293 0.0030891 0.0030852  0.00308123 0.00307719 0.00307308 0.0030689 0.00306464 0.00306032  0.00305592 0.00305145 0.00304692 0.00304231 0.00303764 0.0030329  0.00302809 0.00302321 0.00301826 0.00301325 0.00300817 0.00300302  0.00299781 0.00299253 0.00298718 0.00298177 0.0029763 0.00297076  0.00296516 0.00295949 0.00295376 0.00294797 0.00294212 0.00293621  0.00293023 0.00292419 0.00291809 0.00291194 0.00290572 0.00289944  0.00289311 0.00288671 0.00288026 0.00287375 0.00286718 0.00286056  0.00285388 0.00284715 0.00284036 0.00283351 0.00282662 0.00281966  0.00281266 0.0028056 0.00279849 0.00279133 0.00278412 0.00277685  0.00276954 0.00276217 0.00275476 0.0027473 0.00273979 0.00273223  0.00272462 0.00271697 0.00270927 0.00270153 0.00269374 0.00268591  0.00267803 0.00267011 0.00266214 0.00265413 0.00264608 0.00263799  0.00262986 0.00262169 0.00261347 0.00260522 0.00259693 0.0025886  0.00258023 0.00257183 0.00256339 0.00255491 0.0025464 0.00253785  0.00252927 0.00252065 0.002512 0.00250332 0.00249461 0.00248586  0.00247708 0.00246827 0.00245943 0.00245056 0.00244167 0.00243274  0.00242379 0.0024148 0.0024058 0.00239676 0.0023877 0.00237861  0.0023695 0.00236037 0.00235121 0.00234203 0.00233283 0.0023236  0.00231435 0.00230509 0.0022958 0.00228649 0.00227716 0.00226782  0.00225845 0.00224907 0.00223967 0.00223026 0.00222083 0.00221138  0.00220192 0.00219245 0.00218296 0.00217345 0.00216394 0.00215441  0.00214487 0.00213532 0.00212576 0.00211619 0.00210661 0.00209702  0.00208742 0.00207782 0.0020682 0.00205858 0.00204896 0.00203932  0.00202968 0.00202004 0.00201039 0.00200074 0.00199109 0.00198143  0.00197177 0.00196211 0.00195244 0.00194278 0.00193311] parzen_estimate(np.array([2.0]), (0.0, 1.0)) = (array([-0.5 , -0.495, -0.49 , -0.485, -0.48 , -0.475, -0.47 , -0.465,  -0.46 , -0.455, -0.45 , -0.445, -0.44 , -0.435, -0.43 , -0.425,  -0.42 , -0.415, -0.41 , -0.405, -0.4 , -0.395, -0.39 , -0.385,  -0.38 , -0.375, -0.37 , -0.365, -0.36 , -0.355, -0.35 , -0.345,  -0.34 , -0.335, -0.33 , -0.325, -0.32 , -0.315, -0.31 , -0.305,  -0.3 , -0.295, -0.29 , -0.285, -0.28 , -0.275, -0.27 , -0.265,  -0.26 , -0.255, -0.25 , -0.245, -0.24 , -0.235, -0.23 , -0.225,  -0.22 , -0.215, -0.21 , -0.205, -0.2 , -0.195, -0.19 , -0.185,  -0.18 , -0.175, -0.17 , -0.165, -0.16 , -0.155, -0.15 , -0.145,  -0.14 , -0.135, -0.13 , -0.125, -0.12 , -0.115, -0.11 , -0.105,  -0.1 , -0.095, -0.09 , -0.085, -0.08 , -0.075, -0.07 , -0.065,  -0.06 , -0.055, -0.05 , -0.045, -0.04 , -0.035, -0.03 , -0.025,  -0.02 , -0.015, -0.01 , -0.005, 0. , 0.005, 0.01 , 0.015,  0.02 , 0.025, 0.03 , 0.035, 0.04 , 0.045, 0.05 , 0.055,  0.06 , 0.065, 0.07 , 0.075, 0.08 , 0.085, 0.09 , 0.095,  0.1 , 0.105, 0.11 , 0.115, 0.12 , 0.125, 0.13 , 0.135,  0.14 , 0.145, 0.15 , 0.155, 0.16 , 0.165, 0.17 , 0.175,  0.18 , 0.185, 0.19 , 0.195, 0.2 , 0.205, 0.21 , 0.215,  0.22 , 0.225, 0.23 , 0.235, 0.24 , 0.245, 0.25 , 0.255,  0.26 , 0.265, 0.27 , 0.275, 0.28 , 0.285, 0.29 , 0.295,  0.3 , 0.305, 0.31 , 0.315, 0.32 , 0.325, 0.33 , 0.335,  0.34 , 0.345, 0.35 , 0.355, 0.36 , 0.365, 0.37 , 0.375,  0.38 , 0.385, 0.39 , 0.395, 0.4 , 0.405, 0.41 , 0.415,  0.42 , 0.425, 0.43 , 0.435, 0.44 , 0.445, 0.45 , 0.455,  0.46 , 0.465, 0.47 , 0.475, 0.48 , 0.485, 0.49 , 0.495,  0.5 , 0.505, 0.51 , 0.515, 0.52 , 0.525, 0.53 , 0.535,  0.54 , 0.545, 0.55 , 0.555, 0.56 , 0.565, 0.57 , 0.575,  0.58 , 0.585, 0.59 , 0.595, 0.6 , 0.605, 0.61 , 0.615,  0.62 , 0.625, 0.63 , 0.635, 0.64 , 0.645, 0.65 , 0.655,  0.66 , 0.665, 0.67 , 0.675, 0.68 , 0.685, 0.69 , 0.695,  0.7 , 0.705, 0.71 , 0.715, 0.72 , 0.725, 0.73 , 0.735,  0.74 , 0.745, 0.75 , 0.755, 0.76 , 0.765, 0.77 , 0.775,  0.78 , 0.785, 0.79 , 0.795, 0.8 , 0.805, 0.81 , 0.815,  0.82 , 0.825, 0.83 , 0.835, 0.84 , 0.845, 0.85 , 0.855,  0.86 , 0.865, 0.87 , 0.875, 0.88 , 0.885, 0.89 , 0.895,  0.9 , 0.905, 0.91 , 0.915, 0.92 , 0.925, 0.93 , 0.935,  0.94 , 0.945, 0.95 , 0.955, 0.96 , 0.965, 0.97 , 0.975,  0.98 , 0.985, 0.99 , 0.995, 1. , 1.005, 1.01 , 1.015,  1.02 , 1.025, 1.03 , 1.035, 1.04 , 1.045, 1.05 , 1.055,  1.06 , 1.065, 1.07 , 1.075, 1.08 , 1.085, 1.09 , 1.095,  1.1 , 1.105, 1.11 , 1.115, 1.12 , 1.125, 1.13 , 1.135,  1.14 , 1.145, 1.15 , 1.155, 1.16 , 1.165, 1.17 , 1.175,  1.18 , 1.185, 1.19 , 1.195, 1.2 , 1.205, 1.21 , 1.215,  1.22 , 1.225, 1.23 , 1.235, 1.24 , 1.245, 1.25 , 1.255,  1.26 , 1.265, 1.27 , 1.275, 1.28 , 1.285, 1.29 , 1.295,  1.3 , 1.305, 1.31 , 1.315, 1.32 , 1.325, 1.33 , 1.335,  1.34 , 1.345, 1.35 , 1.355, 1.36 , 1.365, 1.37 , 1.375,  1.38 , 1.385, 1.39 , 1.395, 1.4 , 1.405, 1.41 , 1.415,  1.42 , 1.425, 1.43 , 1.435, 1.44 , 1.445, 1.45 , 1.455,  1.46 , 1.465, 1.47 , 1.475, 1.48 , 1.485, 1.49 , 1.495,  1.5 ]), array([0. , 0. , 0. , 0. , 0. ,  0. , 0. , 0. , 0. , 0. ,  0. , 0. , 0. , 0. , 0. ,  0. , 0. , 0. , 0. , 0. ,  0. , 0. , 0. , 0. , 0. ,  0. , 0. , 0. , 0. , 0. ,  0. , 0. , 0. , 0. , 0. ,  0. , 0. , 0. , 0. , 0. ,  0. , 0. , 0. , 0. , 0. ,  0. , 0. , 0. , 0. , 0. ,  0. , 0. , 0. , 0. , 0. ,  0. , 0. , 0. , 0. , 0. ,  0. , 0. , 0. , 0. , 0. ,  0. , 0. , 0. , 0. , 0. ,  0. , 0. , 0. , 0. , 0. ,  0. , 0. , 0. , 0. , 0. ,  0. , 0. , 0. , 0. , 0. ,  0. , 0. , 0. , 0. , 0. ,  0. , 0. , 0. , 0. , 0. ,  0. , 0. , 0. , 0. , 0. ,  0. , 0. , 0. , 0. , 0. ,  0. , 0. , 0. , 0. , 0. ,  0. , 0. , 0. , 0. , 0. ,  0. , 0. , 0. , 0. , 0. ,  0. , 0. , 0. , 0. , 0. ,  0. , 0. , 0. , 0. , 0. ,  0. , 0. , 0. , 0. , 0. ,  0. , 0. , 0. , 0. , 0. ,  0. , 0. , 0. , 0. , 0. ,  0. , 0. , 0. , 0. , 0. ,  0. , 0. , 0. , 0. , 0. ,  0. , 0. , 0. , 0. , 0. ,  0. , 0. , 0. , 0. , 0. ,  0. , 0. , 0. , 0. , 0. ,  0. , 0. , 0. , 0. , 0. ,  0. , 0. , 0. , 0. , 0. ,  0. , 0. , 0. , 0. , 0. ,  0. , 0. , 0. , 0. , 0. ,  0. , 0. , 0. , 0. , 0. ,  0. , 0. , 0. , 0. , 0. ,  0. , 0. , 0. , 0. , 0. ,  0. , 0. , 0. , 0. , 0. ,  0. , 0. , 0. , 0. , 0. ,  0. , 0. , 0. , 0. , 0. ,  0. , 0. , 0. , 0. , 0. ,  0. , 0. , 0. , 0. , 0. ,  0. , 0. , 0. , 0. , 0. ,  0. , 0. , 0. , 0. , 0. ,  0. , 0. , 0. , 0. , 0. ,  0. , 0. , 0. , 0. , 0. ,  0. , 0. , 0. , 0. , 0. ,  0. , 0. , 0. , 0. , 0. ,  0. , 0. , 0. , 0. , 0. ,  0. , 0. , 0. , 0. , 0. ,  0. , 0. , 0. , 0. , 0. ,  0. , 0. , 0. , 0. , 0. ,  0. , 0. , 0. , 0. , 0. ,  0. , 0. , 0. , 0. , 0. ,  0. , 0. , 0. , 0. , 0. ,  0. , 0. , 0. , 0. , 0. ,  0. , 0. , 0.00318716, 0.00318712, 0.003187 ,  0.0031868 , 0.00318652, 0.00318616, 0.00318573, 0.00318521,  0.00318461, 0.00318394, 0.00318318, 0.00318234, 0.00318143,  0.00318043, 0.00317936, 0.00317821, 0.00317698, 0.00317567,  0.00317428, 0.00317281, 0.00317126, 0.00316964, 0.00316794,  0.00316616, 0.0031643 , 0.00316236, 0.00316034, 0.00315825,  0.00315608, 0.00315383, 0.00315151, 0.0031491 , 0.00314662,  0.00314407, 0.00314144, 0.00313873, 0.00313594, 0.00313308,  0.00313015, 0.00312714, 0.00312405, 0.00312089, 0.00311765,  0.00311434, 0.00311096, 0.0031075 , 0.00310397, 0.00310036,  0.00309668, 0.00309293, 0.0030891 , 0.0030852 , 0.00308123,  0.00307719, 0.00307308, 0.0030689 , 0.00306464, 0.00306032,  0.00305592, 0.00305145, 0.00304692, 0.00304231, 0.00303764,  0.0030329 , 0.00302809, 0.00302321, 0.00301826, 0.00301325,  0.00300817, 0.00300302, 0.00299781, 0.00299253, 0.00298718,  0.00298177, 0.0029763 , 0.00297076, 0.00296516, 0.00295949,  0.00295376, 0.00294797, 0.00294212, 0.00293621, 0.00293023,  0.00292419, 0.00291809, 0.00291194, 0.00290572, 0.00289944,  0.00289311, 0.00288671, 0.00288026, 0.00287375, 0.00286718,  0.00286056, 0.00285388, 0.00284715, 0.00284036, 0.00283351,  0.00282662])) | The original Parzen window estimator suffers from incorrect index mapping and circular window shifting, which silently corrupts density estimates near domain boundaries and causes probability mass loss. | high | Boundary handling errors |
| Cyclum(42) | def calc_pkm(count_matrix, gene_length_vector, is_cell_row = True):  """  Transformation from count matrix to PKM matrix.   :param count_matrix: count matrix  :param gene_length_vector: lengths of genes  :param is_cell_row: if true, cells should be rows  :return:  """   axis = 1 if is_cell_row else 0  count = count_matrix.copy()  # count -> reads per million  scailing_factor = count.sum(axis=axis, keepdims=True) / 1_000_000  rpm = count / scailing_factor  return rpm / gene_length_vector.reshape([-1, 1]) * 1_000 | def calc_pkm(count_matrix, gene_length_vector, is_cell_row=True):  """  count_matrix:  - (cells, genes) if is_cell_row=True  - (genes, cells) otherwise  gene_length_vector: (genes,), unit = bp  """   count = count_matrix.astype(float)  gene_length = gene_length_vector.astype(float)   if is_cell_row:  # cells x genes  total_reads = count.sum(axis=1, keepdims=True)  total_reads[total_reads == 0] = np.nan  rpm = count / total_reads * 1_000_000  pkm = rpm / gene_length[None, :] * 1_000  else:  # genes x cells  total_reads = count.sum(axis=0, keepdims=True)  total_reads[total_reads == 0] = np.nan  rpm = count / total_reads * 1_000_000  pkm = rpm / gene_length[:, None] * 1_000   return pkm | assert calc_pkm_fixed(np.array([  [100, 200, 300],  [400, 500, 600], ]), np.array([1000, 2000, 3000]), is_cell_row=True) == [[166666.66666667 166666.66666667 166666.66666667]  [266666.66666667 166666.66666667 133333.33333333]] | calc_pkm_fixed(np.array([  [100, 200, 300],  [400, 500, 600], ]), np.array([1000, 2000, 3000]), is_cell_row=True) raises ValueError | The original PKM normalization incorrectly broadcasts gene lengths across the count matrix, leading to silent but systematic mis-scaling of expression values under both cell-by-gene and gene-by-cell layouts. | low |  |
| Flye(178) | def _calculate_divergence(qry_seq, trg_seq):  if not qry_seq or not trg_seq:  return 0.0   curr_del = 0  curr_ins = 0   match_count = 0  mis_count = 0  del_count = 0  ins_count = 0   for q, t in zip(qry_seq, trg_seq):  if q == t:  match_count += 1  if curr_del != 0:  del_count += 1  curr_del = 0  if curr_ins != 0:  ins_count += 1  curr_ins = 0  elif q == "-" and t != "-":  curr_del += 1  if curr_ins != 0:  ins_count += 1  curr_ins = 0  elif q != "-" and t == "-":  curr_ins += 1  if curr_del != 0:  del_count += 1  curr_del = 0  elif q != t:  mis_count += 1  if curr_del != 0:  del_count += 1  curr_del = 0  if curr_ins != 0:  ins_count += 1  curr_ins = 0  else:  raise Exception("No alignment conditions fit, {0} {1}".format(q, t))  if curr_del != 0:  del_count += 1  curr_del = 0  if curr_ins != 0:  ins_count += 1  curr_ins = 0   indel_sim_rate = 0.0  total = match_count + mis_count + del_count + ins_count  if total != 0:  indel_sim_rate = match_count / float(total)  return 1 - indel_sim_rate | def _calculate_divergence(qry_seq, trg_seq):  if qry_seq is None or trg_seq is None:  return 0.0   if len(qry_seq) != len(trg_seq):  raise ValueError("Aligned sequences must have the same length")   match = 0  mismatch = 0  indel = 0   for q, t in zip(qry_seq, trg_seq):  if q == "-" and t == "-":  continue # ignore gap-gap  elif q == "-" or t == "-":  indel += 1  elif q == t:  match += 1  else:  mismatch += 1   total = match + mismatch + indel  if total == 0:  return 0.0   return (mismatch + indel) / float(total) | assert _calculate_divergence("ACGT----ACGT", "ACGTACGTACGT") == 0.3333333333333333 assert _calculate_divergence("ACGTACGT", "ACGTACGT") == 0.0 assert _calculate_divergence("ACGTACGT", "ACGAACGA") == 0.25 | _calculate_divergence("ACGT----ACGT", "ACGTACGTACGT") = 0.11111111111111116 | The original divergence computation silently truncates unequal-length alignments and collapses arbitrarily long indels into a single event, leading to systematic underestimation of sequence divergence. | medium | alternative implementation |
| Flye(178) | def _calc_nx(scaffolds_lengths, assembly_len, rate):  n50 = 0  sum_len = 0  l50 = 0  for l in sorted(scaffolds_lengths, reverse=True):  sum_len += l  l50 += 1  if sum_len > rate * assembly_len:  n50 = l  break  return l50, n50 | def _calc_nx(scaffolds_lengths, assembly_len, rate):  n50 = 0  sum_len = 0  l50 = 0  for l in sorted(scaffolds_lengths, reverse=True):  sum_len += l  l50 += 1  if sum_len >= rate * assembly_len:  n50 = l  break  return l50, n50 | assert _calc_nx([100, 200, 300], 500, 0.5) == (1, 300) # assert _calc_nx([100, 200, 300], 500, 0.6) == (1, 300) assert _calc_nx([100, 200, 300], 500, 0.4) == (1, 300) assert _calc_nx([100, 200, 300], 500, 0.3) == (1, 300) # assert _calc_nx([100, 200, 300], 500, 1.0) == (2, 200) assert _calc_nx([100, 200, 300], 500, 0.0) == (1, 300) assert _calc_nx([100, 200, 300], 0, 0.5) == (1, 300) assert _calc_nx([], 500, 0.5) == (0, 0) assert _calc_nx([300], 500, 0.5) == (1, 300) assert _calc_nx([300, 200], 500, 0.5) == (1, 300) assert _calc_nx([300, 200, 100], 500, 0.5) == (1, 300) assert _calc_nx([300, 200, 100], 500, 0.2) == (1, 300) | _calc_nx([100, 200, 300], 500, 0.6) = (2, 200) _calc_nx([100, 200, 300], 500, 1.0) = (3, 100) | The two implementations differ only in the threshold condition used to determine when the cumulative scaffold length reaches the target fraction of the total assembly length. The first version uses a strict inequality (sum_len > rate * assembly_len), meaning it only stops after the cumulative length exceeds the threshold. This can lead to a delayed termination and may underestimate the true NX/N50 value in boundary cases.  In contrast, the second version uses a non-strict inequality (sum_len >= rate * assembly_len), which correctly follows the standard definition of NX/N50 metrics in bioinformatics. According to this definition, the cutoff scaffold is the first one that causes the cumulative length to reach or exceed the target fraction of the total assembly length. Therefore, the second implementation is more accurate and consistent with the accepted biological interpretation of NX/N50 calculations. | medium | domain knowledge violation |
| GSEApy(71) | def normalize(es, esnull):  """normalize the ES(S,pi) and the observed ES(S), separately rescaling  the positive and negative scores by dividing the mean of the ES(S,pi).   return: NES, NESnull  """   nEnrichmentScores = np.zeros(es.shape)  nEnrichmentNulls = np.zeros(esnull.shape)  # esnullmean = np.zeros(es.shape)  # # calculate nESnulls  # for i in range(esnull.shape[0]):  # # NES  # enrNull = esnull[i]  # if es[i] >= 0:  # mes = enrNull[enrNull >= 0].mean()  # nEnrichmentScores[i] = es[i] / mes  # else:  # mes = enrNull[enrNull < 0 ].mean()  # nEnrichmentScores[i] = - es[i] / mes  # esnullmean[i] = mes   # # NESnull  # for j in range(esnull.shape[1]):  # if esnull[i,j] >= 0:  # nEnrichmentNulls[i,j] = esnull[i,j] / esnullmean[i]  # else:  # nEnrichmentNulls[i,j] = - esnull[i,j] / esnullmean[i]   esnull_pos = np.ma.MaskedArray(esnull, mask=(esnull < 0)).mean(axis=1)  esnull_neg = np.ma.MaskedArray(esnull, mask=(esnull >= 0)).mean(axis=1)  esnull_pos = np.array(esnull_pos)  esnull_neg = np.array(esnull_neg)  # NES  nEnrichmentScores = np.where(es >= 0, es / esnull_pos, -es / esnull_neg)  # NES_NULL  nEnrichmentNulls = np.where(  esnull >= 0,  esnull / esnull_pos[:, np.newaxis],  -esnull / esnull_neg[:, np.newaxis],  )   return nEnrichmentScores, nEnrichmentNulls | def normalize(es, esnull, eps=1e-12):  """  Numerically stable and definition-consistent NES normalization.  """   es = np.asarray(es)  esnull = np.asarray(esnull)   n_sets, n_perm = esnull.shape  NES = np.zeros_like(es, dtype=float)  NESnull = np.zeros_like(esnull, dtype=float)   for i in range(n_sets):  null = esnull[i]   pos = null[null > 0]  neg = null[null < 0]   mean_pos = pos.mean() if pos.size > 0 else None  mean_neg = (-neg).mean() if neg.size > 0 else None # abs mean   # ---- normalize observed ES ----  if es[i] >= 0:  if mean_pos is not None:  NES[i] = es[i] / (mean_pos + eps)  elif mean_neg is not None:  NES[i] = es[i] / (mean_neg + eps)  else:  NES[i] = 0.0  else:  if mean_neg is not None:  NES[i] = -es[i] / (mean_neg + eps)  elif mean_pos is not None:  NES[i] = -es[i] / (mean_pos + eps)  else:  NES[i] = 0.0   # ---- normalize null ES ----  for j in range(n_perm):  if null[j] >= 0:  denom = mean_pos if mean_pos is not None else mean_neg  NESnull[i, j] = null[j] / (denom + eps) if denom else 0.0  else:  denom = mean_neg if mean_neg is not None else mean_pos  NESnull[i, j] = -null[j] / (denom + eps) if denom else 0.0   return NES, NESnull | assert normalize(np.array([0.5]), np.array([[-0.2, -0.3, -0.4]])) == (array([1.66666667]), array([[0.66666667, 1. , 1.33333333]])) assert normalize(np.array([-0.6]), np.array([[0.1, 0.2, 0.15]])) == (array([4.]), array([[0.66666667, 1.33333333, 1. ]])) assert normalize(np.array([0.8]), np.array([[0.4, 0.5, -0.3, -0.2]])) == (array([1.77777778]), array([[0.88888889, 1.11111111, 1.2 , 0.8 ]])) | normalize(np.array([0.5]), np.array([[-0.2, -0.3, -0.4]])) = (array([inf]), array([[-0.66666667, -1. , -1.33333333]])) normalize(np.array([-0.6]), np.array([[0.1, 0.2, 0.15]])) = (array([inf]), array([[0.66666667, 1.33333333, 1. ]])) normalize(np.array([0.8]), np.array([[0.4, 0.5, -0.3, -0.2]])) = (array([1.77777778]), array([[ 0.88888889, 1.11111111, -1.2 , -0.8 ]])) | The normalization step may yield NaN or undefined NES values when null enrichment scores lack either positive or negative components, violating the GSEA normalization definition under asymmetric null distributions. | medium | alternative implementation |
| GSEApy(71) | def fdrcorrection(pvals, alpha=0.05):  """benjamini hocheberg fdr correction. inspired by statsmodels"""  # Implement copy from GOATools.  pvals = np.asarray(pvals)  pvals_sortind = np.argsort(pvals)  pvals_sorted = np.take(pvals, pvals_sortind)   ecdffactor = _ecdf(pvals_sorted)  reject = pvals_sorted <= ecdffactor * alpha  if reject.any():  rejectmax = max(np.nonzero(reject)[0])  reject[:rejectmax] = True  pvals_corrected_raw = pvals_sorted / ecdffactor  pvals_corrected = np.minimum.accumulate(pvals_corrected_raw[::-1])[::-1]  del pvals_corrected_raw  pvals_corrected[pvals_corrected > 1] = 1  pvals_corrected_ = np.empty_like(pvals_corrected)  pvals_corrected_[pvals_sortind] = pvals_corrected  del pvals_corrected  reject_ = np.empty_like(reject)  reject_[pvals_sortind] = reject  return reject_, pvals_corrected_ | def fdrcorrection(pvals, alpha=0.05):  """  Benjamini-Hochberg FDR correction (robust version).  """   pvals = np.asarray(pvals, dtype=float)   if pvals.size == 0:  return np.array([], dtype=bool), np.array([], dtype=float)   nan_mask = np.isnan(pvals)  valid_mask = ~nan_mask   pvals_valid = pvals[valid_mask]  m = len(pvals_valid)   reject = np.zeros_like(pvals, dtype=bool)  pvals_corrected = np.full_like(pvals, np.nan, dtype=float)   if m == 0:  return reject, pvals_corrected   order = np.argsort(pvals_valid)  p_sorted = pvals_valid[order]   ecdf = np.arange(1, m + 1) / float(m)   bh_threshold = ecdf * alpha  rejected = p_sorted <= bh_threshold   if rejected.any():  max_i = np.max(np.nonzero(rejected)[0])  rejected[:max_i + 1] = True   p_corr_sorted = p_sorted / ecdf  p_corr_sorted = np.minimum.accumulate(p_corr_sorted[::-1])[::-1]  p_corr_sorted = np.clip(p_corr_sorted, 0, 1)   # map back  reject_valid = np.zeros_like(pvals_valid, dtype=bool)  reject_valid[order] = rejected   pvals_corr_valid = np.empty_like(pvals_valid)  pvals_corr_valid[order] = p_corr_sorted   reject[valid_mask] = reject_valid  pvals_corrected[valid_mask] = pvals_corr_valid   return reject, pvals_corrected | assert fdrcorrection([0.001, 0.04, 0.06, 0.9], 0.05) == (array([ True, False, False, False]), array([0.004, 0.08 , 0.08 , 0.9 ])) assert fdrcorrection([0.001, np.nan, 0.02, 0.8])) == (array([ True, False, True, False]), array([0.003, nan, 0.03 , 0.8 ])) assert fdrcorrection([])) == (array([], dtype=bool), array([], dtype=float64)) assert fdrcorrection([0.001, 0.01, 0.02, 0.2, 0.5]) == (array([ True, True, True, False, False]), array([0.005 , 0.025 , 0.03333333, 0.25 , 0.5 ])) | fdrcorrection([0.001, np.nan, 0.02, 0.8])) = (array([ True, False, True, False]), array([nan, nan, nan, nan])) | Although the implementation follows the Benjamini–Hochberg procedure in principle, an off-by-one error in the rejection propagation and the lack of NaN handling can lead to incorrect hypothesis rejection under realistic input conditions. | low |  |
| bakta(198) | def gc_fraction(seq: str):  gc = seq.count('C') + seq.count('G') + seq.count('S')  length = len(seq)  return 0 if length == 0 else gc / length | def gc_fraction(seq: str):  """  Calculate GC fraction of a sequence.  Counts 'G', 'C', and ambiguous base 'S' (G/C) as GC.  Case-insensitive.  """  if not seq:  return 0.0  seq = seq.upper()  gc = sum(base in ("G", "C", "S") for base in seq)  return gc / len(seq) | assert gc_fraction("acgtgcs") == 0.7142857142857143 assert gc_fraction("GcCgSs") == 1.0 assert gc_fraction("GGCCSS") == 1.0 | gc_fraction("acgtgcs") = 0.0 gc_fraction("GcCgSs") = 0.5 | The original GC fraction implementation implicitly assumes uppercase input, leading to systematic underestimation for lowercase or mixed-case genomic sequences. | medium | domain knowledge violation |
| CNV-FB(23) | def gc_correct(RD, GC):  # correcting gc bias  bincount = np.bincount(GC)  global_rd_ave = np.mean(RD)  for i in range(len(RD)):  if bincount[GC[i]] < 2:  continue  mean = np.mean(RD[GC == GC[i]])  RD[i] = global_rd_ave * RD[i] / mean  return RD | def gc_correct(RD, GC):  RD = np.asarray(RD, dtype=float)  GC = np.asarray(GC)   corrected = RD.copy()  global_rd_ave = RD.mean()   for gc in np.unique(GC):  idx = (GC == gc)  if idx.sum() < 2:  continue  mean = RD[idx].mean()  if mean == 0:  continue  corrected[idx] = global_rd_ave * RD[idx] / mean   return corrected | assert gc_correct(np.array([10.0, 20.0, 30.0, 40.0]), np.array([1, 1, 1, 1])) == [10. 20. 30. 40.] assert gc_correct(np.array([10.0, 20.0]), np.array([1, 2])) == [10. 20.] assert gc_correct_fixed(np.array([10, 20, 30]), np.array([40.1, 40.1, 40.1])) == [10. 20. 30.] | gc_correct_fixed(np.array([10, 20, 30]), np.array([40.1, 40.1, 40.1])) raises TypeError | The function implicitly assumes that GC values are non-negative integers by directly applying np.bincount(GC) and indexing with bincount[GC[i]]. However, in practice GC content is commonly represented as floating-point values (e.g., GC percentage or fraction). When GC is non-integer, the function either raises a runtime error or produces undefined behavior, leading to incorrect or failed GC bias correction. This mismatch between the assumed and actual GC data types constitutes a functional bug. | low |  |
| EMcnv(769) | def EWMA_SEG(breakpointSelects):  segs = []  start = breakpointSelects[0]  itm = 1  edgeSize = 1  for i in range(len(breakpointSelects)):  if i + 1 < len(breakpointSelects):  if itm == 1:  start = breakpointSelects[i]  itm += 1  if breakpointSelects[i + 1] - breakpointSelects[i] < edgeSize:  if i + 1 == len(breakpointSelects) - 1:  if start - edgeSize > 0:  segs.append([start - edgeSize, breakpointSelects[i + 1] + edgeSize])  else:  segs.append([start, breakpointSelects[i + 1]])  elif breakpointSelects[i + 1] - breakpointSelects[i] > edgeSize:  end = breakpointSelects[i]  if start - edgeSize < 0:  segs.append([start, end + edgeSize])  else:  segs.append([start - edgeSize, end + edgeSize])  itm = 1  return segs | def EWMA_SEG(breakpointSelects, edgeSize=1):  if not breakpointSelects:  return []   bps = sorted(breakpointSelects)   segs = []  start = bps[0]  prev = bps[0]   for cur in bps[1:]:  if cur - prev <= edgeSize:  # still in the same segment  prev = cur  else:  # close previous segment  seg_start = max(0, start - edgeSize)  seg_end = prev + edgeSize  segs.append([seg_start, seg_end])  start = cur  prev = cur   seg_start = max(0, start - edgeSize)  seg_end = prev + edgeSize  segs.append([seg_start, seg_end])   return segs | assert EWMA_SEG([10, 11, 12]) == [[9, 13]] assert EWMA_SEG([5, 6, 7, 8]) == [[4, 9]] assert EWMA_SEG([1, 2, 10, 11]) == [[0, 3], [9, 12]] assert EWMA_SEG([3, 5]) == [[2, 4], [4, 6]] | EWMA_SEG([10, 11, 12]) = [] EWMA_SEG([5, 6, 7, 8]) = [] EWMA_SEG([1, 2, 10, 11]) = [[0, 3]] EWMA_SEG([3, 5]) = [[2, 4]] | The original EWMA-based segmentation logic may silently drop the final segment and fails to handle breakpoint gaps equal to the edge size. Segment termination depends on local difference conditions rather than explicit segment closure, leading to incomplete or missing segments in common breakpoint patterns. | high | Boundary handling errors |
| EMcnv(769) | def breakpoint_select(dfArr, sp, upline, dowline):  index = 0  breakpoints = []  for i in dfArr.ewm(span=sp).mean():  if i > upline or i < dowline:  breakpoints.append(index)  index += 1  return breakpoints | def breakpoint_select(dfArr, sp, upline, dowline):  index = 0  breakpoints = []  for i in dfArr.ewm(span=sp, adjust=False).mean():  if i > upline or i < dowline:  breakpoints.append(index)  index += 1  return breakpoints | assert breakpoint_select([1, 2, 3], 19, 3, 2)==[0, 1, 2, 3, 4] | breakpoint_select([1, 2, 3], 19, 3, 2) = [0,1,4] | The only difference between the two functions is the adjust parameter in the exponential weighted moving average (ewm) calculation. The first uses the default adjust=True, which computes the weighted mean by applying weights to all historical data points normalized over the entire window. The second uses adjust=False, performing a recursive calculation that places more weight on recent data points and is computationally more efficient. This results in subtly different smoothing behavior, especially near the start of the data series. | high | Parameter passing errors |
| EMcnv(769) | def merge_segs(segs):  newSegs = []  tm = []  for li in segs:  if len(tm) == 0:  tm = li  newSegs.append(tm)  else:  if li[0] <= tm[1]:  tm = [tm[0], li[1]]  del (newSegs[-1])  newSegs.append(tm)  else:  newSegs.append(li)  tm = li  return newSegs | def merge_segs(segs):  if not segs:  return []   segs = sorted(segs, key=lambda x: x[0])   merged = []  cur_start, cur_end = segs[0]   for start, end in segs[1:]:  if start <= cur_end: # overlap or touch  cur_end = max(cur_end, end)  else:  merged.append([cur_start, cur_end])  cur_start, cur_end = start, end   merged.append([cur_start, cur_end])  return merged | assert merge_segs([[1, 10], [3, 5]]) == [[1, 10]] assert merge_segs([[5, 8], [1, 3], [2, 6]]) == [[1, 8]] assert merge_segs([[1, 3], [3, 5], [6, 8]]) == [[1, 5], [6, 8]] | merge_segs([[1, 10], [3, 5]]) = [[1, 5]] merge_segs([[5, 8], [1, 3], [2, 6]]) = [[5, 6]] | The original merge_segs implementation assumes sorted, non-nested intervals and incorrectly merges overlapping segments by overwriting the right boundary, which can shrink segments when one interval is contained within another. The corrected version explicitly sorts inputs and merges intervals using the maximum end coordinate. | high | Boundary handling errors |
| EMcnv(769) | def first_of(elems):  """Return the first element of the input."""  return elems[0] | def first_of(elems):  """Return the first element of the input."""  if not elems:  return None  else:  return elems[0] | assert first_of([]) == None assert first_of([1]) == 1 assert first_of(['a', 'b', 'c']) == 'a' assert first_of([None]) == None assert first_of([0]) == 0 | first_of([]) raises IndexError | The two versions differ in how they handle empty inputs. The original version assumes the input is non-empty and directly returns the first element, which can lead to an IndexError if the input is empty. The revised version adds an explicit check and returns None when the input is empty, making it more robust and safer for general use. | low |  |
| EMcnv(769) | def normalize(A):  """Normalize matrix columns."""  return A / A.sum(axis=0) | def normalize(A):  """Normalize matrix columns."""  num_rows = len(A)  num_cols = len(A[0])  normalized_A = [[0] * num_cols for _ in range(num_rows)]   for j in range(num_cols):  column_sum = 0  for i in range(num_rows):  column_sum += A[i][j]   if column_sum == 0:  for i in range(num_rows):  normalized_A[i][j] = A[i][j]  else:  for i in range(num_rows):  normalized_A[i][j] = A[i][j] / column_sum   return normalized_A | assert normalize([[1, 2], [3, 4]]) == [[0.25, 0.3333333333333333], [0.75, 0.6666666666666666]] assert normalize([[0, 0], [0, 0]]) == [[0, 0], [0, 0]] assert normalize([[1, 0], [0, 0]]) == [[1.0, 0], [0.0, 0]] assert normalize([[1, 2, 3], [4, 5, 6]]) == [[0.2, 0.2857142857142857, 0.3333333333333333], [0.8, 0.7142857142857143, 0.6666666666666666]] assert normalize([[0, 1], [0, 2], [0, 3]]) == [[0, 0.16666666666666666], [0, 0.3333333333333333], [0, 0.5]] assert normalize([[1, 2], [0, 0]]) == [[1.0, 1.0], [0.0, 0.0]] assert normalize([[5, 0], [0, 0]]) == [[1.0, 0], [0.0, 0]] assert normalize([[1], [2], [3]]) == [[0.16666666666666666], [0.3333333333333333], [0.5]] assert normalize([[0]]) == [[0]] assert normalize([[1, 1], [1, 1]]) == [[0.5, 0.5], [0.5, 0.5]] | normalize([[0, 0], [0, 0]]) == [[nan, nan], [nan, nan]] normalize([[1, 0], [0, 0]]) == [[1.0, nan], [0.0, nan]] normalize([[0, 1], [0, 2], [0, 3]]) == [[nan, 0.16666666666666666], [nan, 0.3333333333333333], [nan, 0.5]] normalize([[5, 0], [0, 0]]) == [[1.0, nan], [0.0, nan]] normalize([[1], [2], [3]]) == [[nan]] | Both functions normalize the columns of a matrix such that each column sums to 1. The first version leverages NumPy for a concise and efficient implementation but does not handle zero-sum columns, which may result in NaN values. The second version, written in pure Python, explicitly checks for zero-sum columns and retains the original values in such cases, making it more robust but less efficient and more verbose. | low |  |
| EMcnv(769) | def FDRThres(x, q, stdev):  """False discovery rate (FDR) threshold."""  M = len(x)  if M < 2:  return 0   m = np.arange(1, M + 1) / M  x_sorted = np.sort(np.abs(x))[::-1]  p = 2 * (1 - stats.norm.cdf(x_sorted, stdev)) # like R "pnorm"  # Get the largest index for which p <= m*q  indices = np.nonzero(p <= m * q)[0]  if len(indices):  T = x_sorted[indices[-1]]  else:  logging.debug(  "No passing p-values: min p=%.4g, min m=%.4g, q=%s", p[0], m[0], q  )  T = x_sorted[0] + 1e-16 # ~= 2^-52, like MATLAB "eps"  return T | def FDRThres(x, q, stdev):  """False discovery rate (FDR) threshold."""  M = len(x)  if M < 2:  return 0   m = np.arange(1, M + 1) / M  x_sorted = np.sort(np.abs(x))[::-1]  p = 2 * (1 - stats.norm.cdf(x_sorted, loc=0, scale=stdev)) # like R "pnorm"  # Get the largest index for which p <= m*q  indices = np.nonzero(p <= m * q)[0]  if len(indices):  T = x_sorted[indices[-1]]  else:  logging.debug(  "No passing p-values: min p=%.4g, min m=%.4g, q=%s", p[0], m[0], q  )  T = x_sorted[0] + 1e-16 # ~= 2^-52, like MATLAB "eps"  return T | assert calculateFDRThreshold([0.1, 0.2, 0.3], 0.05,0.05) == 0.1 assert calculateFDRThreshold([-0.1, -0.2, -0.3], 0.05,0.05) == 0.1  assert calculateFDRThreshold([0.1, 0.2, 0.3], 0.05,0) == 0.3000000000000001 assert calculateFDRThreshold([0.1, 0.2, 0.3], 0.05,1) == 0.3000000000000001 assert calculateFDRThreshold([0.1, 0.2, 0.3, 0.4, 0.5], 0.05,0.1) == 0.3  assert calculateFDRThreshold([0.5, 0.4, 0.3, 0.2, 0.1], 0.05,0.1) == 0.3  assert calculateFDRThreshold([1.0, 2.0, 3.0], 0.05,0.5) == 1.0 assert calculateFDRThreshold([1.0, 2.0, 3.0], 0.05,0.9) == 2.0 | calculateFDRThreshold([0.1, 0.2, 0.3], 0.05,0.05) == 0.3000000000000001 calculateFDRThreshold([-0.1, -0.2, -0.3], 0.05,0.05) == 0.3000000000000001 calculateFDRThreshold([0.1, 0.2, 0.3, 0.4, 0.5], 0.05,0.1) == 0.5000000000000001 calculateFDRThreshold([0.5, 0.4, 0.3, 0.2, 0.1], 0.05,0.1) == 0.5000000000000001 calculateFDRThreshold([1.0, 2.0, 3.0], 0.05,0.5) == 3.0 calculateFDRThreshold([1.0, 2.0, 3.0], 0.05,0.9) == 3.0 | When stdev ≠ 1 and the parameter is mistakenly interpreted as loc (mean) rather than scale (standard deviation) due to incorrect argument positioning, the computed p-value will be incorrect, leading to a deviation in the resulting threshold. | high | Parameter passing errors |
| EMcnv(769) | def p_adjust_bh(p):  p = np.asfarray(p)  by_descend = p.argsort()[::-1]  by_orig = by_descend.argsort()  steps = float(len(p)) / np.arange(len(p), 0, -1)  q = np.minimum(1, np.minimum.accumulate(steps * p[by_descend]))  return q[by_orig] | def p_adjust_bh(p):  p = np.asarray(p, dtype=float)   q = np.full_like(p, np.nan)   # valid p-values  mask = np.isfinite(p)  p_valid = p[mask]   if p_valid.size == 0:  return q   if np.any((p_valid < 0) \| (p_valid > 1)):  raise ValueError("p-values must be between 0 and 1")   order = np.argsort(p_valid)[::-1]  order_rev = order.argsort()   n = p_valid.size  steps = n / np.arange(n, 0, -1)   q_valid = np.minimum(1, np.minimum.accumulate(steps * p_valid[order]))  q[mask] = q_valid[order_rev]   return q | assert p_adjust_bh(np.array([0.01, np.nan, 0.2, 0.03])) == [0.03 nan 0.2 0.045] assert p_adjust_bh([0.01, 0.02, 0.2, 0.5]) == [0.04 0.04 0.26666667 0.5 ] p_adjust_bh([0.01, 1.2, 0.2]) raises ValueError | p_adjust_bh(np.array([0.01, np.nan, 0.2, 0.03])) = [nan nan nan nan] p_adjust_bh([0.01, 1.2, 0.2]) == [0.03 1. 0.3 ] | The implementation of the Benjamini–Hochberg procedure is numerically correct for valid inputs but lacks robustness to missing or invalid p-values, where the presence of NaNs or out-of-range values may silently corrupt multiple adjusted p-values. | low |  |
| EMcnv(769) | def HaarConv(signal,  weight,  stepHalfSize,  ):  signalSize = len(signal)  if stepHalfSize > signalSize:  return np.zeros(signalSize, dtype=np.float_)   result = np.zeros(signalSize, dtype=np.float_)  if weight is not None:  highWeightSum = weight[:stepHalfSize].sum()   highNonNormed = (weight[:stepHalfSize] * signal[:stepHalfSize]).sum()   lowWeightSum = highWeightSum   lowNonNormed = -highNonNormed   for k in range(1, signalSize):  highEnd = k + stepHalfSize - 1  if highEnd >= signalSize:  highEnd = signalSize - 1 - (highEnd - signalSize)  lowEnd = k - stepHalfSize - 1  if lowEnd < 0:  lowEnd = -lowEnd - 1   if weight is None:  result[k] = result[k - 1] + signal[highEnd] + signal[lowEnd] - 2 * signal[k - 1]  else:  lowNonNormed += signal[lowEnd] * weight[lowEnd] - signal[k - 1] * weight[k - 1]  highNonNormed += signal[highEnd] * weight[highEnd] - signal[k - 1] * weight[k - 1]  lowWeightSum += weight[k - 1] - weight[lowEnd]  highWeightSum += weight[highEnd] - weight[k - 1]   result[k] = math.sqrt(stepHalfSize / 2) * (lowNonNormed / lowWeightSum +  highNonNormed / highWeightSum)   if weight is None:  stepNorm = math.sqrt(2. * stepHalfSize)  result[1:signalSize] /= stepNorm   return result | def HaarConv(signal, weight, stepHalfSize):  signal = np.asarray(signal, dtype=float)  n = len(signal)   if stepHalfSize <= 0 or 2 * stepHalfSize > n:  raise ValueError("Require 2 * stepHalfSize <= signal length")   result = np.zeros(n, dtype=float)   for k in range(stepHalfSize, n - stepHalfSize):  left = signal[k - stepHalfSize:k]  right = signal[k:k + stepHalfSize]   if weight is None:  val = right.sum() - left.sum()  result[k] = val / math.sqrt(2 * stepHalfSize)  else:  w = weight[:stepHalfSize]  lw = w.sum()  if lw == 0:  continue  val = (right @ w) / lw - (left @ w) / lw  result[k] = math.sqrt(stepHalfSize / 2) * val   return result | assert HaarConv(np.array([0, 0, 1, 1, 1, 0, 0], dtype=float), np.ones(len(signal)), 2) == [ 0. 0. 1. 0.5 -0.5 0. 0. ] assert HaarConv(np.ones(10), np.zeros(10), 2) == [0. 0. 0. 0. 0. 0. 0. 0. 0. 0.] assert HaarConv(np.arange(20, dtype=float),None, 3) == [0. 0. 0. 3.67423461 3.67423461 3.67423461  3.67423461 3.67423461 3.67423461 3.67423461 3.67423461 3.67423461  3.67423461 3.67423461 3.67423461 3.67423461 3.67423461 0.  0. 0. ] | HaarConv(np.array([0, 0, 1, 1, 1, 0, 0], dtype=float), np.ones(len(signal)), 2) = [ 0. 0.5 1. 0.5 -0.5 -1. -0.5] HaarConv(np.ones(10), np.zeros(10), 2) = [ 0. nan nan nan nan nan nan nan nan nan] HaarConv(np.arange(20, dtype=float),None, 3) = [0. 2.04124145 3.26598632 3.67423461 3.67423461 3.67423461  3.67423461 3.67423461 3.67423461 3.67423461 3.67423461 3.67423461  3.67423461 3.67423461 3.67423461 3.67423461 3.67423461 3.67423461  3.26598632 2.04124145] | The Haar convolution implementation exhibits incorrect boundary and weighting semantics when weights are provided, as window weights are indexed globally rather than relative to the convolution window, leading to broken translation invariance and unstable normalization near boundaries. | medium | alternative implementation |
| LMADCNV(9) | def estimate_location_and_mad(data: np.ndarray):  n_samples = len(data)  n_support = int(n_samples * 0.6)  X_sorted = np.sort(np.ravel(data))  diff = X_sorted[n_support:] - X_sorted[: (n_samples - n_support)]  halves_start = np.where(diff == np.min(diff))[0]  # take the middle points' mean to get the robust location estimate  location = (  0.5 * (X_sorted[n_support + halves_start] + X_sorted[halves_start]).mean()  )  median = X_sorted[n_support // 2 + halves_start[0]]  abs_diff = np.abs(X_sorted[halves_start[0] : n_support + halves_start[0]] - median)  mad = np.mean(abs_diff)  return location, mad | def estimate_location_and_mad(data: np.ndarray):  X = np.sort(np.ravel(data))  n = len(X)  if n < 2:  return X[0], 0.0   n_support = max(1, int(n * 0.6))  diff = X[n_support:] - X[: n - n_support]  start = np.argmin(diff)   support = X[start : start + n_support]   # robust location: midpoint mean  location = 0.5 * (support[0] + support[-1])   # proper median inside support  median = np.median(support)   # proper MAD  mad = np.median(np.abs(support - median))   return location, mad | assert estimate_location_and_mad(np.array([0, 0, 1, 1, 1, 1, 50])) == (0.5, 0.5) assert estimate_location_and_mad(np.array([1, 2, 3, 4, 5, 6])) == (2.0, 1.0) | estimate_location_and_mad(np.array([1, 2, 3, 4, 5, 6])) = (3.5, 0.6666666666666666) | The original implementation inconsistently computes location and scale by deriving the median from a global index rather than the selected support interval and by using a mean-based deviation, whereas the fixed version consistently operates within the shortest support interval and applies a proper median-based MAD estimator, restoring statistical robustness. | medium | alternative implementation |
| PangenomeX(46) | def gen_bub(differ, a, b):  segments = []  start = None  for i in range(len(differ)):  if differ[i] > 0 or differ[i] < 0:  if start is None:  start = i  else:  if start is not None:  length = i - start  if length >= 5000:  segments.append((start, length))  start = None  result = []  for start, length in segments:  a_segment = a[start:start + length]  b_segment = b[start:start + length]  logr = np.round(np.log2(np.mean(a_segment) / np.mean(b_segment)), 2)  result.append([start, length, logr])   return result | def gen_bub(differ, a, b, min_len=5000):  segments = []  start = None  n = len(differ)   for i in range(n):  if differ[i] != 0:  if start is None:  start = i  else:  if start is not None:  length = i - start  if length >= min_len:  segments.append((start, length))  start = None   if start is not None:  length = n - start  if length >= min_len:  segments.append((start, length))   result = []  for start, length in segments:  a_segment = a[start:start + length]  b_segment = b[start:start + length]   ma = np.mean(a_segment)  mb = np.mean(b_segment)   if mb <= 0 or ma <= 0 or np.isnan(ma) or np.isnan(mb):  logr = np.nan  else:  logr = np.round(np.log2(ma / mb), 2)   result.append([start, length, logr])   return result | assert gen_bub(np.ones(6000), np.ones(6000) * 4, np.ones(6000) * 2) == [[0, 6000, 1.0]] assert gen_bub(np.repeat([0, 1], [1000, 6000]), np.ones(7000) * 4, np.ones(7000) * 2) == [[1000, 6000, 1.0]] assert gen_bub(np.concatenate([np.zeros(1000), np.ones(6000), np.zeros(1000)]), np.ones(7000) * 4, np.ones(7000) * 2) == [[1000, 6000, 1.0]] | gen_bub(np.ones(6000), np.ones(6000) * 4, np.ones(6000) * 2) = [] gen_bub(np.repeat([0, 1], [1000, 6000]), np.ones(7000) * 4, np.ones(7000) * 2) = [] | The original implementation fails to emit the final segment when the non-zero region extends to the end of the array, leading to silent loss of valid segments; the fixed version explicitly handles trailing segments and adds robustness checks for invalid log-ratio computation. | high | Boundary handling errors |
| waveCNV(34) | def merge_CNV_segment(RD,index,balance,gap):  segment = [-1]  start = -1  for i in range(len(index)):  if start == -1:  start = index[i]  elif index[i]-index[i-1]<=10 and i != len(index)-1:  continue  else:  segment.append([start,index[i-1]])  start = index[i]  segment.pop(0)  answer = []  for start,end in segment:  if end - start > gap:  tumor_mean = np.mean(RD[start:end])  if tumor_mean > balance:  answer.append([start,end,1])  elif balance == -1:  answer.append([start, end, 0])  else:  answer.append([start, end, -1])  return answer | def merge_CNV_segment(RD, index, balance, gap):  if len(index) == 0:  return []   index = np.sort(index)   segments = []  start = index[0]   for i in range(1, len(index)):  if index[i] - index[i-1] <= 10:  continue  else:  segments.append((start, index[i-1]))  start = index[i]   segments.append((start, index[-1]))   answer = []  for start, end in segments:  if end - start > gap:  tumor_mean = np.mean(RD[start:end+1])   if tumor_mean > balance:  label = 1  elif balance == -1:  label = 0  else:  label = -1   answer.append([start, end, label])   return answer | assert merge_CNV_segment(np.ones(100), [10, 30], 0.5, 5) == [] assert merge_CNV_segment(np.ones(100), [10, 11, 12, 13, 14], 0.5, 2) ==[[10, 14, 1]] assert merge_CNV_segment(np.ones(200), [10, 11, 12, 50, 51, 52], 0.5, 2) == [] | merge_CNV_segment(np.ones(100), [10, 11, 12, 13, 14], 0.5, 2) ==[[10, 13, 1]] | The original implementation fails to flush the final CNV segment when indices are contiguous up to the last element, leading to systematic loss of terminal CNV regions. Additionally, improper slice boundaries further bias mean depth estimation. | high | Boundary handling errors |
| waveCNV(34) | def scale_01_data(rawData):  # normalize the raw dataset  dim = rawData.shape[1]  # the rawData has at least 2 raw, 1 for signal 1 for lable  minList = [np.amin(rawData[:,x]) for x in range(0, dim)]  maxList = [np.amax(rawData[:,x])+0.001 for x in range(0, dim)]  # add the [0] and [1] because there is a 'raw of lable', and 0.001 to avoid 1  toZero = rawData - np.array(minList)  normData = toZero / (np.array(maxList) - np.array(minList))  return(normData) | def scale_01_data(rawData, normalize_label=False):  rawData = np.asarray(rawData, dtype=float)   if not normalize_label:  data = rawData[:, :-1]  label = rawData[:, -1:]  else:  data = rawData  label = None   minv = np.min(data, axis=0)  maxv = np.max(data, axis=0)  span = maxv - minv   # avoid division by zero  span[span == 0] = 1.0   normData = (data - minv) / span   if label is not None:  normData = np.hstack([normData, label])   return normData | assert scale_01_data(np.array([  [10, 100, 1],  [20, 200, 0],  [30, 300, 1] ])) == [[0. 0. 1. ]  [0.5 0.5 0. ]  [1. 1. 1. ]] assert scale_01_data(np.array([  [0, 0],  [1, 1] ])) == [[0. 0.]  [1. 1.]] | scale_01_data(np.array([  [10, 100, 1],  [20, 200, 0],  [30, 300, 1] ])) = [[0. 0. 0.999001 ]  [0.499975 0.4999975 0. ]  [0.99995 0.999995 0.999001 ]] scale_01_data(np.array([  [0, 0],  [1, 1] ])) = [[0. 0. ]  [0.999001 0.999001]] | The original normalization applies min–max scaling indiscriminately to all columns and artificially perturbs the maximum value, leading to biased feature scaling and corrupted label semantics. The corrected version enforces proper feature-only normalization and numerical stability. | medium | alternative implementation |
| altanalyze | def covertLogFoldToNonLog(log_val):  try:  if float(log_val)<0: nonlog_val = (-1/math.pow(2,(float(log_val))))  else: nonlog_val = (math.pow(2,float(log_val)))  except Exception: nonlog_val = log_val  return str(nonlog_val) | def covertLogFoldToNonLog(log_val):  try:  if float(log_val) < 0:  nonlog_val = (-1 * math.pow(2, (float(log_val))))  else:  nonlog_val = (math.pow(2, float(log_val)))  except Exception:  nonlog_val = log_val  return str(nonlog_val) | assert covertLogFoldToNonLog("-1") == '-0.5' assert covertLogFoldToNonLog("0") == '1.0' assert covertLogFoldToNonLog("1") == '2.0' assert covertLogFoldToNonLog("2") == '4.0' assert covertLogFoldToNonLog("abc") == 'abc' assert covertLogFoldToNonLog("-abc") == '-abc' assert covertLogFoldToNonLog("-0.5") == '-0.7071067811865476' assert covertLogFoldToNonLog("3.5") == '11.313708498984761' assert covertLogFoldToNonLog("-3.5") == '-0.08838834764831845' | covertLogFoldToNonLog("-1") == '-2.0' covertLogFoldToNonLog("-0.5") == '-1.414213562373095' covertLogFoldToNonLog("-3.5") == '-11.31370849898476' | The two versions differ in how they handle negative log fold change values. For positive values, both versions are identical, computing 2^x. For negative values, the first version computes -1 / 2^x, which is equivalent to -2^(-x), effectively flipping the sign of the exponent before computing the power. The second version computes -1 * 2^x, which keeps the exponent as-is and simply negates the result. The second version is correct. The proper way to reverse a log2 fold change is to apply 2^x directly regardless of sign, then negate for negative inputs. This preserves symmetry: a log2 FC of 2 gives 4 (4-fold up), and a log2 FC of -2 gives -0.25 (4-fold down, represented as a fraction). The first version incorrectly inverts the exponent for negative inputs, breaking this symmetry and producing -4 instead of -0.25 for an input of -2. | high | State Misalignment Error |
| altanalyze | def covertLogFoldToNonLogFloat(log_val):  try:  if float(log_val)<0: nonlog_val = (-1/math.pow(2,(float(log_val))))  else: nonlog_val = (math.pow(2,float(log_val)))  except Exception: nonlog_val = log_val  return str(nonlog_val) | def covertLogFoldToNonLog(log_val):  try:  if float(log_val) < 0:  nonlog_val = (-1 * math.pow(2, (float(log_val))))  else:  nonlog_val = (math.pow(2, float(log_val)))  except Exception:  nonlog_val = log_val  return str(nonlog_val) | assert covertLogFoldToNonLog("-1") == -2.0 assert covertLogFoldToNonLog("0") == 1.0 assert covertLogFoldToNonLog("1") == 2.0 assert covertLogFoldToNonLog("2") == 4.0 assert covertLogFoldToNonLog("-0.5") == -1.414213562373095 assert covertLogFoldToNonLog("3.5") == 11.313708498984761 | covertLogFoldToNonLog("-1") == -0.5 covertLogFoldToNonLog("0") == 1.0 covertLogFoldToNonLog("1") == 2.0 covertLogFoldToNonLog("2") == 4.0 covertLogFoldToNonLog("-0.5") == -0.7071067811865476 covertLogFoldToNonLog("3.5") == 11.313708498984761 | The two versions differ in how they handle negative log fold change values. For positive values, both versions are identical, computing 2^x. For negative values, the first version computes -1 / 2^x, which is equivalent to -2^(-x), effectively flipping the sign of the exponent before computing the power. The second version computes -1 * 2^x, which keeps the exponent as-is and simply negates the result. The second version is correct. The proper way to reverse a log2 fold change is to apply 2^x directly regardless of sign, then negate for negative inputs. This preserves symmetry: a log2 FC of 2 gives 4 (4-fold up), and a log2 FC of -2 gives -0.25 (4-fold down, represented as a fraction). The first version incorrectly inverts the exponent for negative inputs, breaking this symmetry and producing -4 instead of -0.25 for an input of -2. | high | State Misalignment Error |
| altanalyze | def predictIDSource(id,system_codes):  au = ['A','B','C','D','E','F','G','H','I','J','K','L','M','N','O','P','Q','R','S','T','U','V','W','X','Y','Z']  nm = ['1','2','3','4','5','6','7','8','9']  affy_suffix = '_at'; ensembl_prefix = 'ENS'; source_data = ''; id_type = 'Symbol'; id_types={}  if len(id)>3:  if affy_suffix == id[-3:]: id_type = 'Affymetrix'  elif ensembl_prefix == id[:3]: id_type = 'Ensembl'  elif id[2] == '.': id_type = 'Unigene'  elif (id[0] in au and id[1] in nm) or '_' in id: id_type = 'UniProt'  else:  try:  int_val = int(id)  if len(id) == 7: id_type = 'Affymetrix' ###All newer Affymetrix transcript_cluster_ids and probesets (can be mistaken for EntrezGene IDs)  else: id_type = 'EntrezGene'  except ValueError: null = []  try: id_types[id_type]+=1  except Exception: id_types[id_type]=1   ###If the user changes the names of the above id_types, we need to verify that the id_type is in the database  if len(id_type)>0 and len(id_types)>0:  id_type_count=[]  for i in id_types:  id_type_count.append((id_types[i],i))  id_type_count.sort()  #print id_type_count  id_type = id_type_count[-1][-1]  for code in system_codes:  if system_codes[code] == id_type: source_data = id_type  return source_data | def predictIDSource(id, system_codes):  au = ['A','B','C','D','E','F','G','H','I','J','K','L','M','N','O','P','Q','R','S','T','U','V','W','X','Y','Z']  nm = ['1','2','3','4','5','6','7','8','9']  affy_suffix = '_at'  ensembl_prefix = 'ENS'  source_data = ''  id_type = ''  id_types = {}    if len(id) > 3:  if id.endswith(affy_suffix):  id_type = 'Affymetrix'  elif id.startswith(ensembl_prefix):  id_type = 'Ensembl'  elif '.' in id[2:]:  id_type = 'Unigene'  elif (id[0] in au and id[1] in nm) or '_' in id:  id_type = 'UniProt'  else:  try:  int_val = int(id)  if len(id) == 7:  id_type = 'Affymetrix'  else:  id_type = 'EntrezGene'  except ValueError:  pass    try:  id_types[id_type] += 1  except KeyError:  id_types[id_type] = 1   if id_type and id_types:  id_type_count = []  for i in id_types:  id_type_count.append((id_types[i], i))  id_type_count.sort()  id_type = id_type_count[-1][-1]    for code in system_codes:  if system_codes[code] == id_type:  source_data = id_type    return source_data | assert predictIDSource('A123456_at', {'code1': 'Affymetrix', 'code2': 'Ensembl'}) == 'Affymetrix' assert predictIDSource('ENS123456', {'code1': 'Affymetrix', 'code2': 'Ensembl'}) == 'Ensembl' assert predictIDSource('123.456', {'code1': 'Unigene', 'code2': 'UniProt'}) == 'Unigene' assert predictIDSource('A1_code', {'code1': 'UniProt', 'code2': 'Affymetrix'}) == 'UniProt' assert predictIDSource('1234567', {'code1': 'Affymetrix', 'code2': 'EntrezGene'}) == 'Affymetrix' assert predictIDSource('invalid_id', {'code1': 'Affymetrix', 'code2': 'EntrezGene'}) == '' assert predictIDSource('A1B2C3', {'code1': 'UniProt', 'code2': 'Ensembl'}) == 'UniProt' assert predictIDSource('123456', {'code1': 'EntrezGene', 'code2': 'Affymetrix'}) == 'EntrezGene' | predictIDSource('123.456', {'code1': 'Unigene', 'code2': 'UniProt'}) = '' | The two versions implement similar ID prediction logic, but the second version improves the correctness and robustness of the implementation in several important ways.  Compared with the first version, the second version uses more idiomatic string operations (startswith, endswith); replaces the meaningless statement null = [] with proper exception handling using pass; narrows exception handling from Exception to KeyError, avoiding unintended error suppression; fixes the placement of the id_types counting logic so that all detected ID types are consistently recorded, rather than only numeric IDs; improves readability and maintainability through clearer formatting and structure.  As a result, the second version provides more reliable and consistent behavior, and is generally a more correct and maintainable implementation of the intended functionality. | medium | alternative implementation |
| altanalyze | def avg(array):  return sum(array) / len(array) | def avg(array):  if len(array) == 0:  raise ValueError("division by zero")  return sum(array) / len(array) | assert avg([]) raises ValueError assert avg([1, 2, 3, 4, 5]) == 3.0 assert avg([-1, 0, 1]) == 0.0 assert avg([0]) == 0.0 assert avg([10, 20, 30, 40, 50]) == 30.0 | avg([]) raises ValueError: division by zero | The two versions behave identically for non-empty input arrays, but differ in how they handle empty input.  The first version relies on Python’s runtime behavior and raises a ZeroDivisionError implicitly when the input list is empty. In contrast, the second version explicitly checks for empty input and raises a ValueError with a clear and intentional error condition.  By adding explicit input validation, the second version improves robustness, readability, and maintainability, making the function behavior more predictable and semantically correct. | low |  |
| altanalyze | def calculateRPKM(condition_count_db,exon_len_db,biotype_to_examine):  """Determines the total number of reads in a sample and then calculates RPMK relative to a pre-determined junction length (60).  60 was choosen, based on Illumina single-end read lengths of 35 (5 nt allowed overhand on either side of the junction)"""  ### Get the total number of mapped reads  mapped_reads={}  for condition in condition_count_db:  mapped_reads[condition]=0  count_db = condition_count_db[condition]  for key in count_db:  read_count = count_db[key]  mapped_reads[condition]+=float(read_count)    ### Use the average_total_reads when no counts reported such that 0 counts are comparable   average_total_reads = 0  for i in mapped_reads:  average_total_reads+=mapped_reads[i]  if testImport == 'yes':  print('condition:',i,'total reads:',mapped_reads[i])  average_total_reads = average_total_reads/len(condition_count_db)  if testImport == 'yes':  print('average_total_reads:',average_total_reads)  k=0  c=math.pow(10.0,9.0)  for key in exon_len_db:  try:  for condition in condition_count_db:  total_mapped_reads = mapped_reads[condition]  try: read_count = float(condition_count_db[condition][key])+1 ###This adjustment allows us to obtain more realist folds where 0 is compared and use log2  except KeyError: read_count = 1 ###Was zero, but needs to be one for more realistic log2 fold calculations  if biotype_to_examine == 'junction': region_length = 60.0  else:  try: region_length = exon_len_db[key]  except Exception: continue ### This should only occur during testing (when restricting to one or few chromosomes)  if read_count == 1: ###This adjustment allows us to obtain more realist folds where 0 is compared and use log2  rpkm = c*(float(read_count)/(float(average_total_reads)*region_length))   try:  if region_length == 0:  region_length = abs(int(key[2]-key[1]))  rpkm = c*(read_count/(float(total_mapped_reads)*region_length))  except Exception:  print(condition, key)  print('Error Encountered... Exon or Junction of zero length encoutered... RPKM failed... Exiting AltAnalyze.')  print('This error may be due to inconsistent file naming. If both exon and junction sample data is present, make sure they are named propperly.')  print('For example: cancer1__exon.bed, cancer1__junction.bed (double underscore required to match these samples up)!')  print([read_count,total_mapped_reads,region_length]);k=1; forceError  condition_count_db[condition][key] = str(rpkm) ### Replace original counts with RPMK  except Exception:  if k == 1: kill  null=[]  return condition_count_db | def calculateRPKM(condition_count_db, exon_len_db, biotype_to_examine):  mapped_reads = {}  for condition in condition_count_db:  mapped_reads[condition] = 0  count_db = condition_count_db[condition]  for key in count_db:  read_count = count_db[key]  mapped_reads[condition] += float(read_count)   average_total_reads = 0  for i in mapped_reads:  average_total_reads += mapped_reads[i]  average_total_reads = average_total_reads / len(condition_count_db)   c = 10**9  for key in exon_len_db:  try:  for condition in condition_count_db:  total_mapped_reads = mapped_reads[condition]  try:  read_count = float(condition_count_db[condition][key]) + 1  except KeyError:  read_count = 1  if biotype_to_examine == 'junction':  region_length = 60.0  else:  try:  region_length = exon_len_db[key]  except Exception:  continue  if region_length == 0:  region_length = abs(int(key[2] - key[1]))  rpkm = c * (read_count / (float(total_mapped_reads) * region_length))  condition_count_db[condition][key] = str(rpkm)  except Exception:  continue  return condition_count_db | assert calculateRPKM({'condition1': {'gene1': 100, 'gene2': 200}}, {'gene1': 300, 'gene2': 400}, 'gene') == {'condition1': {'gene1': '1122222.2222222222', 'gene2': '1675000.0'}} assert calculateRPKM({'condition1': {'gene1': 100, 'gene2': 200}}, {'gene1': 0, 'gene2': 400}, 'gene') == {'condition1': {'gene1': 100, 'gene2': '1675000.0'}} assert calculateRPKM({'condition1': {'gene1': 100, 'gene2': 200}}, {'gene1': 300, 'gene2': 400}, 'junction') == {'condition1': {'gene1': '5611111.111111111', 'gene2': '11166666.666666666'}} assert calculateRPKM({'condition1': {'gene1': 100}}, {'gene1': 300}, 'gene') == {'condition1': {'gene1': '3366666.6666666665'}} assert calculateRPKM({'condition1': {'gene1': 100, 'gene2': 200}, 'condition2': {'gene1': 150, 'gene2': 250}}, {'gene1': 300, 'gene2': 400}, 'gene') == {'condition1': {'gene1': '1122222.2222222222', 'gene2': '1675000.0'}, 'condition2': {'gene1': '1258333.3333333333', 'gene2': '1568750.0'}} assert calculateRPKM({'condition1': {'gene1': 100, 'gene2': 200}}, {'gene1': 300, 'gene2': 0}, 'gene') == {'condition1': {'gene1': '1122222.2222222222', 'gene2': 200}} assert calculateRPKM({'condition1': {'gene1': 100, 'gene2': 200}}, {'gene1': 300, 'gene2': 400}, 'nonexistent_biotype') == {'condition1': {'gene1': '1122222.2222222222', 'gene2': '1675000.0'}} | assert pickOptimalCriticalExons((1, 2), (1, 3), (2, 3), (2, 4), []) == [(1, 3), (2, 4)] | Both compute RPKM by dividing read count by total mapped reads and region length, scaled by 10⁹. Both add 1 to every raw count to avoid log₂(0) problems, and both fall back to a region-length estimate from the key coordinates when the stored length is zero. The most significant structural improvement is the ordering of operations. Version 1 places the region_length == 0 guard and the final RPKM formula inside a nested try block that follows an earlier, unconditional RPKM assignment — making the control flow genuinely hard to reason about. Version 2 resolves region_length first, then computes RPKM exactly once, so the formula is always applied to a valid denominator.Version 2 also eliminates a silent dead-code bug. Version 1 tries to use average_total_reads when a gene has zero mapped reads, but that result is immediately overwritten by the unconditional RPKM line below it — so the intended special-case behaviour never executes. Version 2 makes no such promise and therefore cannot silently break it. Finally, Version 2 replaces the deeply nested try/except structure and the hard-crash-via-undefined-name pattern (forceError, kill) with a simple, flat continue on error, which is far easier to audit. | medium | alternative implementation |
| altanalyze | def pickOptimalCriticalExons(e1b5, e1a5, e2a3, e2b3, critical_exon):  e1a5_block, e1a5_reg = e1a5  e2a3_block, e2a3_reg = e2a3  e1b5_block, e1b5_reg = e1b5  e2b3_block, e2b3_reg = e2b3   if e1a5_block == e1b5_block:  if e1a5_reg < e1b5_reg: critical_exon += [e1b5]  elif e1a5_reg != e1b5_reg: critical_exon += [e1a5]  if e2a3_block == e2b3_block:  if e2a3_reg < e2b3_reg: critical_exon += [e2a3]  elif e2a3_reg != e2b3_reg: critical_exon += [e2b3]  return critical_exon | def pickOptimalCriticalExons(e1b5, e1a5, e2a3, e2b3, critical_exon):  e1a5_block, e1a5_reg = e1a5  e2a3_block, e2a3_reg = e2a3  e1b5_block, e1b5_reg = e1b5  e2b3_block, e2b3_reg = e2b3   if e1a5_block == e1b5_block:  if e1a5_reg < e1b5_reg:  critical_exon.append(e1b5)  elif e1a5_reg != e1b5_reg:  critical_exon.append(e1a5)  if e2a3_block == e2b3_block:  if e2a3_reg < e2b3_reg:  critical_exon.append(e2b3)  elif e2a3_reg != e2b3_reg:  critical_exon.append(e2a3)  return critical_exon | assert pickOptimalCriticalExons((1, 2), (1, 1), (2, 3), (2, 4), []) == [(1, 2), (2, 4)] assert pickOptimalCriticalExons((1, 2), (1, 3), (2, 3), (2, 4), []) == [(1, 3), (2, 4)] assert pickOptimalCriticalExons((1, 2), (1, 1), (2, 4), (2, 3), []) == [(1, 2), (2, 4)] assert pickOptimalCriticalExons((1, 2), (1, 2), (2, 3), (2, 4), []) == [(2, 4)] assert pickOptimalCriticalExons((1, 2), (1, 1), (2, 3), (2, 3), []) == [(1, 2)] assert pickOptimalCriticalExons((1, 2), (1, 3), (2, 4), (2, 4), []) == [(1, 3)] | pickOptimalCriticalExons((1, 2), (1, 1), (2, 3), (2, 4), []) = [(1, 2), (2, 3)] pickOptimalCriticalExons((1, 2), (1, 3), (2, 3), (2, 4), []) == [(1, 3), (2, 3)] | The two versions implement nearly identical logic for selecting critical exons, but they differ in the handling of the 3' exon selection condition. In the first version, the comparison logic for e2a3_reg and e2b3_reg is inconsistent with the corresponding 5' exon logic, causing the function to select the smaller region in one branch and the larger region in another. The second version corrects this inconsistency by applying a uniform selection strategy across both 5' and 3' regions, resulting in more coherent and semantically consistent behavior. Additionally, the second version replaces the less explicit += [item] syntax with append(), improving readability and maintainability. Overall, the second version provides a more correct and internally consistent implementation of the intended exon selection logic. | high | State Misalignment Error |
| altanalyze | def aaselect(nt_length):  ### Convert to protein length  if (float(nt_length)/3) == (int(nt_length)/3):  return nt_length/3  else:  return int(string.split(str(float(nt_length)/3),'.')[0])+1 | def aaselect(nt_length):  ### Convert to protein length  if nt_length % 3 == 0:  return nt_length // 3  else:  return nt_length // 3 + 1 | assert aaselect(9) == 3 # assert aaselect(10) == 4 assert aaselect(12) == 4 assert aaselect(15) == 5 assert aaselect(18) == 6 # assert aaselect(20) == 7 assert aaselect(21.0) == 7.0 assert aaselect(22.5) == 8 # assert aaselect(25) == 9 assert aaselect(30) == 10 | aaselect(10) = 3.3333333333333335 aaselect(20) = 6.666666666666667 aaselect(25) = 8.333333333333334 | The two versions differ mainly in how they handle integer division during the migration from Python 2 to Python 3. Both implementations are intended to compute the ceiling value of nt_length / 3 when converting nucleotide length to amino acid length. However, the original version relies on division semantics that behaved differently in Python 2, where integer division was performed automatically for integer operands. In Python 3, the / operator always performs floating-point division, causing the original condition logic to become incorrect and potentially produce inconsistent results. The revised version fixes this issue by using explicit integer arithmetic (// and %) to implement the ceiling operation directly, making the behavior stable, predictable, and consistently correct across Python 3 environments. | low |  |
| altanalyze | def getAllListCombinationsLong(a):  ls1 = ['a1','a2','a3']  ls2 = ['b1','b2','b3']  ls3 = ['c1','c2','c3']  ls = ls1,ls2,ls3    list_len_db={}  for ls in a:  list_len_db[len(x)]=[]  print(len(list_len_db), list_len_db);sys.exit()  if len(list_len_db)==1 and 1 in list_len_db:  ### Just simply combine non-complex data  r=[]  for i in a:  r+=i  else:  #http://code.activestate.com/recipes/496807-list-of-all-combination-from-multiple-lists/  r=[[]]  for x in a:  t = []  for y in x:  for i in r:  t.append(i+[y])  r = t  return r | def getAllListCombinationsLong(a):  ls1 = ['a1','a2','a3']  ls2 = ['b1','b2','b3']  ls3 = ['c1','c2','c3']  ls = ls1,ls2,ls3    list_len_db={}  for x in a:  list_len_db[len(x)] = []    if len(list_len_db) == 1 and 1 in list_len_db:  r = []  for i in a:  r += i  else:  r = [[]]  for x in a:  t = []  for y in x:  for i in r:  t.append(i + [y])  r = t  return r | assert getAllListCombinationsLong([['x1']]) == [['x1']] assert getAllListCombinationsLong([['x1'], ['y1']]) == [['x1', 'y1']] assert getAllListCombinationsLong([['x1', 'x2'], ['y1']]) == [['x1', 'y1'], ['x2', 'y1']] assert getAllListCombinationsLong([['x1'], ['y1', 'y2']]) == [['x1', 'y1'], ['x1', 'y2']] assert getAllListCombinationsLong([['x1', 'x2'], ['y1', 'y2']]) == [['x1', 'y1'], ['x2', 'y1'], ['x1', 'y2'], ['x2', 'y2']] assert getAllListCombinationsLong([['x1', 'x2'], ['y1', 'y2'], ['z1']]) == [['x1', 'y1', 'z1'], ['x2', 'y1', 'z1'], ['x1', 'y2', 'z1'], ['x2', 'y2', 'z1']] assert getAllListCombinationsLong([['x1', 'x2'], ['y1', 'y2'], ['z1', 'z2']]) == [['x1', 'y1', 'z1'], ['x2', 'y1', 'z1'], ['x1', 'y2', 'z1'], ['x2', 'y2', 'z1'], ['x1', 'y1', 'z2'], ['x2', 'y1', 'z2'], ['x1', 'y2', 'z2'], ['x2', 'y2', 'z2']] assert getAllListCombinationsLong([['x1', 'x2', 'x3'], ['y1', 'y2'], ['z1', 'z2']]) == [['x1', 'y1', 'z1'], ['x2', 'y1', 'z1'], ['x3', 'y1', 'z1'], ['x1', 'y2', 'z1'], ['x2', 'y2', 'z1'], ['x3', 'y2', 'z1'], ['x1', 'y1', 'z2'], ['x2', 'y1', 'z2'], ['x3', 'y1', 'z2'], ['x1', 'y2', 'z2'], ['x2', 'y2', 'z2'], ['x3', 'y2', 'z2']] assert getAllListCombinationsLong([['x1', 'x2'], ['y1', 'y2', 'y3'], ['z1']]) == [['x1', 'y1', 'z1'], ['x2', 'y1', 'z1'], ['x1', 'y2', 'z1'], ['x2', 'y2', 'z1'], ['x1', 'y3', 'z1'], ['x2', 'y3', 'z1']] assert getAllListCombinationsLong([['x1'], ['y1', 'y2', 'y3'], ['z1', 'z2']]) == [['x1', 'y1', 'z1'], ['x1', 'y2', 'z1'], ['x1', 'y3', 'z1'], ['x1', 'y1', 'z2'], ['x1', 'y2', 'z2'], ['x1', 'y3', 'z2']] | getAllListCombinationsLong([['x1']]) = ['x1'] getAllListCombinationsLong([['x1'], ['y1']]) = ['x1', 'y1'] | The two versions are intended to generate all combinations across multiple input lists, but the first version contains several issues that prevent the function from working correctly. In the original implementation, the loop incorrectly references an undefined variable (x instead of ls), which leads to a runtime NameError. In addition, the presence of sys.exit() causes the program to terminate before the combination-generation logic can execute, making the function incomplete and unusable in practice. The revised version fixes the variable reference, removes the premature program termination, and preserves the intended Cartesian-product logic, allowing the function to execute correctly and consistently return all expected list combinations. | high | State Misalignment Error |
| deepcpg | def mcc(y, z):  """Compute Matthew's correlation coefficient."""  tp, tn, fp, fn = contingency_table(y, z)  return (tp * tn - fp * fn) /\  K.sqrt((tp + fp) * (tp + fn) * (tn + fp) * (tn + fn)) | def mcc(y, z):  """Compute Matthew's correlation coefficient."""  tp = sum(1 for i in range(len(y)) if y[i] == 1 and z[i] == 1)  tn = sum(1 for i in range(len(y)) if y[i] == 0 and z[i] == 0)  fp = sum(1 for i in range(len(y)) if y[i] == 0 and z[i] == 1)  fn = sum(1 for i in range(len(y)) if y[i] == 1 and z[i] == 0)    denominator = ((tp + fp) * (tp + fn) * (tn + fp) * (tn + fn)) ** 0.5  if denominator == 0:  return 0 # To handle the case where the denominator is zero    return (tp * tn - fp * fn) / denominator | assert mcc([1, 1, 0, 0], [1, 1, 0, 0]) == '1.0' assert mcc([1, 1, 1, 1], [1, 1, 1, 1]) == '0' assert mcc([0, 0, 0, 0], [0, 0, 0, 0]) == '0' assert mcc([1, 0, 1, 0], [1, 0, 1, 0]) == '1.0' assert mcc([1, 0, 0, 1], [0, 1, 1, 0]) == '-1.0' assert mcc([1, 1, 0, 0], [0, 0, 1, 1]) == '-1.0' assert mcc([1, 0, 0, 0], [0, 1, 1, 1]) == '-1.0' assert mcc([0, 1, 1, 0], [1, 0, 0, 1]) == '-1.0' | mcc([1, 1, 1, 1], [1, 1, 1, 1]) raises error mcc([0, 0, 0, 0], [0, 0, 0, 0]) raises error | The two implementations compute Matthew’s Correlation Coefficient (MCC) using the same underlying formula, but they differ significantly in how they handle edge cases involving zero-valued denominators. In the original version, the denominator is computed directly without validation, which can lead to a division-by-zero error when one or more contingency table terms are zero. As a result, the function may fail at runtime for highly imbalanced or degenerate classification inputs.  The revised version improves robustness by explicitly checking whether the denominator is zero before performing the division. When this occurs, it safely returns 0 instead of raising an exception, allowing the function to handle edge cases gracefully and maintain stable execution. In addition, the revised implementation returns a numeric value rather than converting the result to a string, making the output more suitable for downstream numerical analysis. Overall, the second version provides a more reliable and practically usable implementation, especially for real-world datasets where zero-count conditions can occur frequently. | low |  |
| deepcpg | def in_which(x, ys, ye):  """Return index of positions in intervals.   Returns for positions `x[i]` index `j`, s.t. `ys[j] <= x[i] <= ye[j]`, or  -1 if `x[i]` is not overlapped by any interval.  Intervals must be non-overlapping!   Parameters  ----------  x : list  list of positions.  ys: list  list with start of interval sorted in ascending order.  ye: list  list with end of interval.   Returns  -------  :class:`numpy.ndarray`  n:class:`numpy.ndarray` with indices of overlapping intervals or -1.  """  n = len(ys)  m = len(x)  rv = np.empty(m, dtype=np.int)  rv.fill(-1)  i = 0  j = 0  while i < n and j < m:  while j < m and x[j] <= ye[i]:  if x[j] >= ys[i]:  rv[j] = i  j += 1  i += 1  return rv | def in_which(x, ys, ye):  """Return index of positions in intervals.   Returns for positions `x[i]` index `j`, s.t. `ys[j] <= x[i] <= ye[j]`, or  -1 if `x[i]` is not overlapped by any interval.  Intervals must be non-overlapping!   Parameters  ----------  x : list  list of positions.  ys: list  list with start of interval sorted in ascending order.  ye: list  list with end of interval.   Returns  -------  :class:`numpy.ndarray`  n:class:`numpy.ndarray` with indices of overlapping intervals or -1.  """  n = len(ys)  m = len(x)  rv = [-1] * m  i = 0  j = 0  while i < n and j < m:  while j < m and x[j] <= ye[i]:  if x[j] >= ys[i]:  rv[j] = i  j += 1  i += 1  return rv | assert in_which([1, 2, 3], [0, 4], [2, 5]) == [0, 0, -1] assert in_which([1, 2, 3], [0, 2], [2, 5]) == [0, 0, 1] assert in_which([1, 2, 3], [0, 1], [1, 5]) == [0, 1, 1] assert in_which([1, 2, 3], [0, 3], [2, 5]) == [0, 0, 1] assert in_which([1, 2, 3], [0, 3], [3, 5]) == [0, 0, 0] assert in_which([1, 2, 3], [0, 0], [0, 5]) == [1, 1, 1] assert in_which([1, 2, 3], [], []) == [-1, -1, -1] assert in_which([], [0, 4], [2, 5]) == [] assert in_which([1], [0, 4], [2, 5]) == [0] assert in_which([5], [0, 4], [2, 5]) == [1] | raises error | The two versions implement the same interval-mapping algorithm and produce identical numerical results, but they differ in implementation robustness and compatibility. The first version relies on np.int when creating the NumPy array, which has been deprecated in recent NumPy versions and can lead to warnings or errors in modern environments. This makes the implementation less future-proof and potentially unstable across different library versions.  The second version improves compatibility by avoiding deprecated NumPy types and instead uses a standard Python list for initialization, which ensures broader compatibility and eliminates dependency on deprecated APIs. | high | Parameter passing errors |
